# Programmable Light-Driven Color Tuning of Perovskite Quantum Dots

**DOI:** 10.1021/acscentsci.5c01651

**Published:** 2025-11-13

**Authors:** Pragyan Jha, Nikolai Mukhin, Jinge Xu, Christopher H.J. Moran, Arup Ghorai, Felix N. Castellano, Milad Abolhasani

**Affiliations:** † Dept. of Chemical and Biomolecular Engineering, 6798 North Carolina State University, Raleigh, North Carolina 27606, United States; ‡ Dept. of Chemistry, 6798 North Carolina State University, Raleigh, North Carolina 27695, United States

## Abstract

Precise, sustainable, and scalable bandgap tuning of metal halide perovskite (MHP) nanocrystals (NCs) is critical for their integration into advanced optoelectronic and photocatalytic systems. Photoinduced anion exchange reactions (PIAERs) enable uniform halide delivery with spatiotemporal control, yet their complex parameter space has limited mechanistic understanding and rational optimization. Here, we introduce a material-efficient fluidic self-driving laboratory (FSDL) that integrates a single-droplet microfluidic photoreactor, multimodal *in situ* spectroscopy, and a multiobjective Bayesian optimization framework to navigate the ∼10^6^-dimensional design space of PIAERs autonomously. Through machine learning-guided exploration of this coupled parameter landscape, the FSDL rapidly identifies synthesis conditions that simultaneously maximize photoluminescence quantum yield and minimize emission line width for any target emission wavelength across the UV–visible spectrum. Mechanistic trends derived from surrogate modeling revealed distinct kinetic regimes for Br^–^→Cl^–^ and Br^–^→I^–^ exchanges, governed respectively by reaction time and photon flux, enabling reaction-specific tuning strategies. Critically, synthesis protocols discovered at the droplet scale (∼10 μL) were directly translated to continuous-flow operation (∼50–250 mL·day^–1^) without reoptimization, maintaining optical performance and establishing knowledge scalability across 4 orders of magnitude in throughput, with low energy demand. This study demonstrates a reproducible, mechanistically informed, and industrially relevant route for programmable light-directed bandgap tuning in MHP NCs.

## Graphical abstract

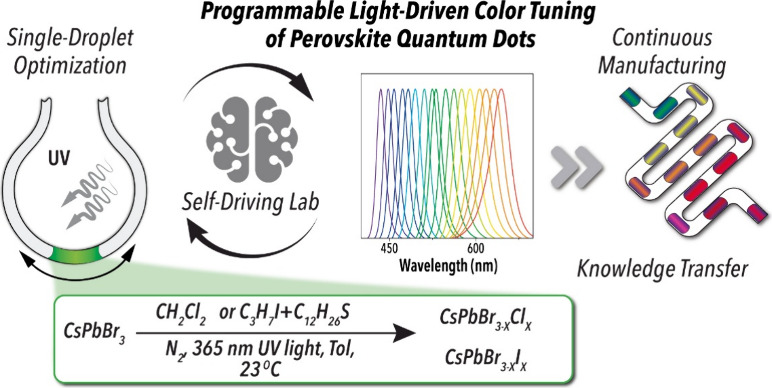

## Introduction

Metal halide perovskite (MHP) nanocrystals (NCs) have emerged as versatile light-harvesting and light-emitting materials that address critical needs in sustainable energy conversion and photocatalysis.^1,2^ Their defect-tolerant electronic structure, high absorption coefficients, and composition-tunable bandgap enable efficient charge generation and transport at wavelengths spanning the entire solar spectrum, positioning MHP NCs as promising candidates for low-temperature processed photovoltaics, displays, light-emitting diodes, and photoredox catalysis.^3–6^ Practical deployment of these technologies, however, requires NCs with application-specific bandgaps that can be reproduced at scale with minimal environmental footprint.^5–7^ Precise, sustainable, and scalable bandgap tuning is therefore central to unlocking the full potential of MHP NCs. Bandgap of MHP NCs can be modulated by (i) varying the halide or A-site cation composition of ABX_3_ (X = Cl, Br, I) NCs, (ii) controlling quantum confinement through particle size, or (iii) introducing lattice strain via heterostructuring.^1,8–11^ Among these strategies, compositional tuning through halide exchange is particularly attractive because it decouples optical design from demanding nucleation kinetics, allowing for continuous wavelength adjustment after colloidal synthesis. Conventional salt-driven halide exchange, however, proceeds at diffusion-limited rates, often producing abrupt reaction fronts with additional impurities, inhomogeneous anion distributions, and broadened emission profiles.^12–14^ Photoinduced anion exchange reactions (PIAERs) circumvent these drawbacks by photolyzing haloalkanes *in situ*, releasing halide ions in a temporally programmed manner that matches the reaction kinetics of NCs.^11,15^ This photochemical route offers uniform ion delivery and tight spatiotemporal control.^16^ Yet the performance of PIAERs depends on a high-dimensional set of coupled variables, including photon flux, precursor concentration, illumination reaction time, solvent polarity, ligand density, and mass transfer rates which interact nonlinearly to govern the final photoluminescence quantum yield (PLQY), peak emission wavelength (λ_em_), and emission line width (*i.e*., full width at half-maximum, fwhm) of MHP NCs.^8,17–22^

Searching such a high-dimensional parameter space with multiple competing output properties by intuition or one-factor-at-a-time studies is inefficient and prone to bias.^23,24^ Fluidic self-driving laboratories (FSDLs) which are closed-loop platforms that integrate programmable flow reactors, real-time material diagnostics, and machine learning (ML)-guided experiment selection–provide a data-driven route to navigate these complex material synthesis landscapes.^7,25–27^ FSDLs can learn from each experiment, propose the next most-promising reaction condition, and converge on globally optimal recipes. However, most self-driving laboratories, including FSDLs, remain confined to discovery throughput; translating their materials chemistry and synthetic routes to large scales is hampered by changes in photon transport, mixing, and heat transfer that alter reaction trajectories.^25,28^

In this work, we present a material-efficient and scalable FSDL for light-directed bandgap tuning of MHP NCs via programmable PIAER. The developed FSDL integrates a single-droplet microfluidic reactor, multimodal *in situ* spectroscopy, and a multiobjective Bayesian optimization (BO) framework to efficiently explore the multioutput space of PIAERs in a scalable manner. Specifically, we close the scalability gap of the existing SDLs by directly transferring knowledge from a material-efficient discovery mode that processes droplets of ∼10 μL per experiment to a continuous manufacturing mode that outputs ∼50–250 mL day^–1^ of product.^7^ The ability to preserve optical performance and material quality across 4 orders of magnitude in volumetric throughput establishes knowledge scalability as one of the central engineering contributions of the developed FSDL.^29,30^

The data-driven FSDL rapidly learns the relationships between reaction conditions and key optical outputs (Pareto-front mapping), enabling the discovery of optimal synthesis protocols that produce high-performing MHP NCs with target emission wavelengths, high PLQYs, and narrow emission line widths. We demonstrate the ability of the FSDL to converge on optimal reaction conditions across the UV–vis spectrum within 24 h and show that the discovered MHP NCs’ synthetic routes can be seamlessly transferred to continuous-flow synthesis for scalable production. This work addresses key scientific and engineering gaps in light-directed NC synthesis by providing a quantitative understanding of PIAER, resolving trade-offs across competing optical metrics, and establishing a reproducible and scalable workflow for compositionally tunable MHP NCs.

## Results and Discussion

To realize programmable, scalable, and compositionally tunable MHP NCs via PIAERs, we developed an FSDL, shown in Figure 1A, B, that integrates microscale droplet reactors with multimodal *in situ* diagnostics and multiobjective BO. This microfluidic platform enables precise exploration of the high-dimensional PIAER parameter space and addresses both the material efficiency and scalability gaps that have limited prior SDLs.^21,31^

**Figure 1:**
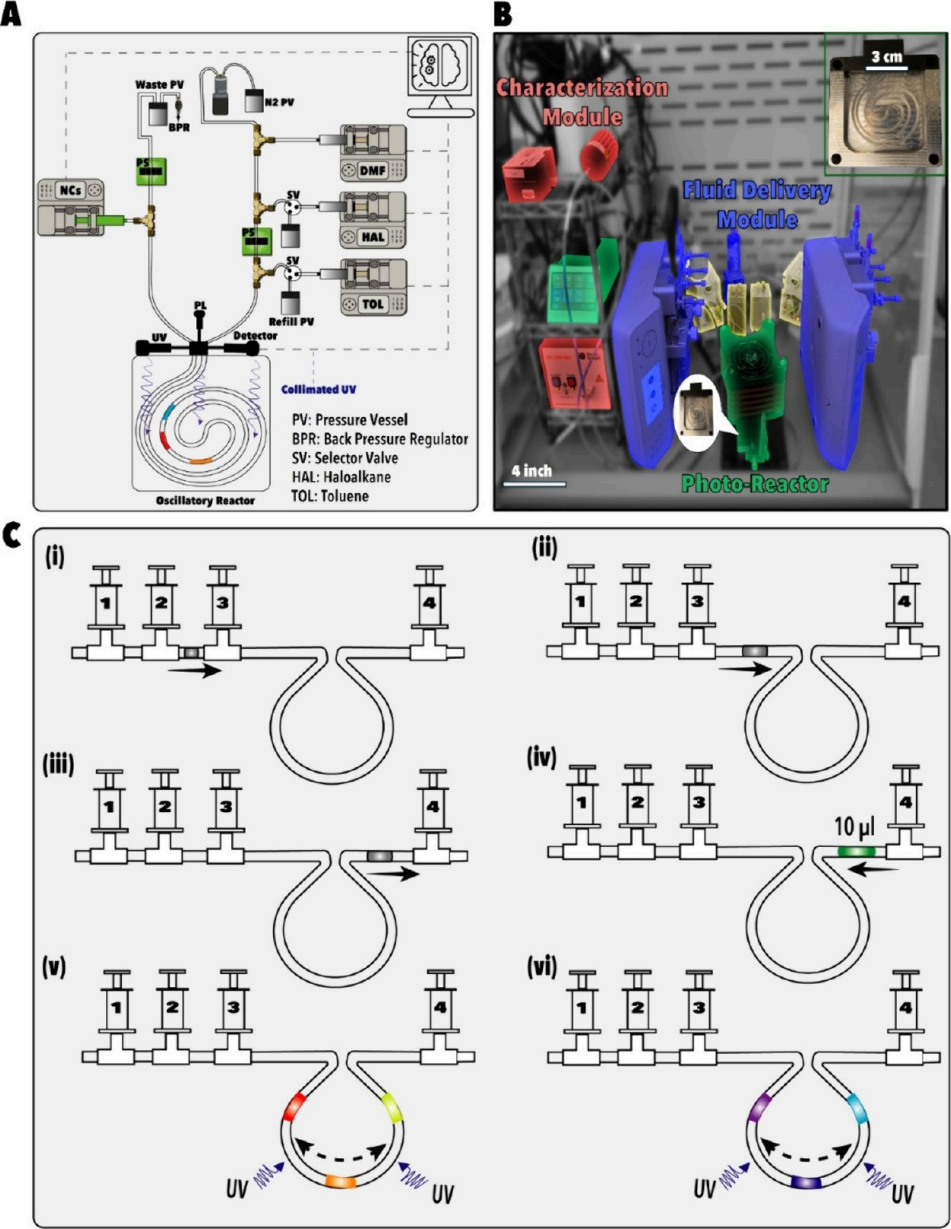
Developed autonomous microfluidic platform for photoinduced anion exchange reactions of MHP NCs. (A) Schematic of the modular microfluidic system comprising: (i) programmable syringe pumps (dimethylformamide (DMF), haloalkane (HAL), toluene (TOL)) precursor and pressure vessel (PV); (ii) phase sensor (PS); (iii) a 3-way selector valve (SV) for reagent switching and fluidic line priming; (iv) a single-droplet microfluidic photoreactor integrated with a collimated UV light and optical detection for real-time UV/vis absorption and photoluminescence (PL) monitoring of PIAER. Automated process control is managed via a computer interface. (B) Photograph of the SDFL hardware, color-coded with its different functional modules. (C) Stepwise droplet formation and reaction protocol: (i–iv) Three precursors are aspirated sequentially from syringes 1–3 (1-DMF, 2-haloalkane, 3-toluene), followed by NC injection from syringe 4; (iv) a 10 μL reactive droplet is dispensed into the microfluidic photoreactor loop; (v–vi) the reactive droplet is oscillated within the UV-illuminated photoreactor for a specific reaction time, where color changes represent evolving PL due to PIAER (Video S1).

### FSDL Hardware

The core of the FSDL consists of a microfluidic photoreactor (Figure 1 **)** that operates in a single-droplet mode for material-efficient PIAER. The microfluidic photoreactor utilizes a UV-transparent Teflon tubing coupled with a tunable 365 nm collimated UV illumination (0–120 pmol.s^–1^), (Figure S1), and an oscillatory mixing to ensure uniform photon exposure (Figure 1C). The microfluidic photoreactor consumes ∼100 times less material than batch reactors while providing continuous, high-quality data streams for predictive ML model training.^7,8,32^ A custom-designed optical flow cell enables real-time acquisition of UV–vis absorption and PL spectra at ∼60 s intervals (Figures 2A-D), enabling comprehensive *in situ* PIAER monitoring (Figure S1B). The resulting high-resolution data set enables detailed exploration of the PIAER’s parameter space. Key optical features, including absorbance at 365 nm (*A*_365_), λ_em_, fwhm, integrated PL area (*PL*_A_), PL intensity (*PL*_I_), and a proxy for PL quantum yield (*P*_PLQY_), are extracted automatically using a Python-based script (Equations S1–S9). *P*_PLQY_ is defined as the integrated *PL*_A_ normalized by *A*_365_. This normalization corrects for variations in NCs concentration and excitation efficiency, ensuring that the metric reflects the fraction of absorbed photons re-emitted rather than raw *PL*_I_.^2,8,33^

**Figure 2:**
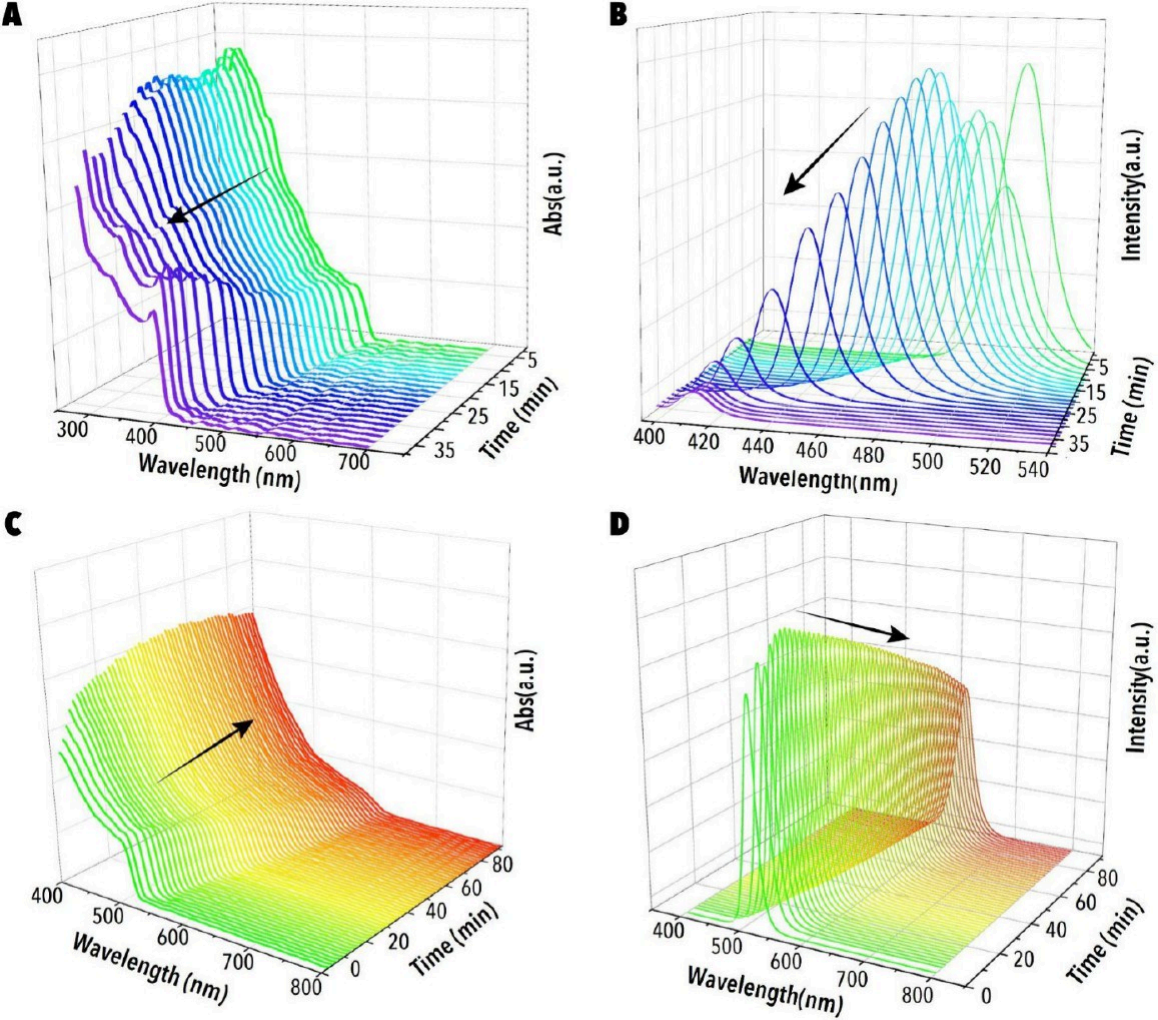
In situ spectral evolution of CsPbBr_3_ NCs undergoing photoinduced anion exchange reactions (PIAER) inside a single-droplet microfluidic photoreactor. Time-resolved (A) UV–vis and (B) PL spectra during chloride-side PIAER, where CsPbBr_3_ NCs react with dichloromethane (DCM) under a photon flux of 8 pmol·s^–1^. A consistent blue-shift in both spectra indicates progressive chloride incorporation and widening of the bandgap. Time-resolved (C) UV–vis and (D) PL spectral progression during iodide-side PIAER with 2-iodopropane and 10 mM of 1-dodecanethiol (DSH) under a photon flux of 20 pmol·s^–1^, showing a red-shift indicative of iodide incorporation and reduced bandgap. Experimental conditions: [CsPbBr_3_] = 4.2 μm; [DCM] = 3.2 M; [2-iodopropane] = 0.11 M; *T* = 23 °C.

We validated the FSDL’s *in situ* characterization module by benchmarking it against conventional benchtop spectroscopy (FS5, Edinburgh Instruments). The proxy *P*_PLQY_ exhibited strong correlations (*R* ^2^ > 0.97) with both *ex-situ*-measured integrated *PL*_A_ and the absolute PLQY values, confirming the analytical reliability of the FSDL (Figure S2). To quantitatively calibrate the UV illumination conditions inside the microfluidic photoreactor, we determined the incident photon flux by chemical actinometry using 4,4′-dimethylazobenzene (DMAB) as the actinometer (Figure S3).^34,35^ The E–Z photoisomerization kinetics of DMAB under 365 nm irradiation provided a direct correlation between light-emitting diode (LED) input current and photon flux, established by numerical integration of the absorbance decay profiles and comparison with measured LED output power.

Next, to evaluate PIAER’s temporal resolution and photon-dose dependence, we conducted a systematic study under alternating periods of on and off UV illumination to assess the extent to which PIAER’s progression depends on photon exposure. A 10 μL reactive droplet containing CsPbBr_3_ NCs and either dichloromethane (DCM) or 2-iodopropane was introduced into the microfluidic photoreactor and the evolution of the emission peak was tracked in real time. A stepwise spectral shift was observed *only* during periods of UV illumination, shifting toward shorter wavelengths (blue shift) in the presence of DCM and toward longer wavelengths (red shift) with 2-iodopropane, consistent with the progressive incorporation of chloride and iodide, respectively (Figures 3A-B). No change in the MHP NC emission was observed during the dark intervals (UV illumination was turned off). As shown in Figures 3A-B, λ_em_ evolves in discrete steps during UV-on intervals and remains constant during UV-off periods (gray-shaded). These results confirm that halide exchange is strictly light-activated and can be precisely modulated by adjusting the duration and intensity of the photoexcitation. Quantitative kinetic analysis of the UV-on segments confirmed photon-flux-dependent reaction rates, establishing a linear correlation between the intrinsic rate constant and the actinometry-calibrated photon flux (Figure S16). Control experiments performed under identical conditions but without UV illumination showed no measurable spectral change, verifying that the halide exchange is inactive in the dark. Continuous UV irradiation tests further confirmed negligible photothermal heating of the microfluidic reactor, with temperature maintained at 23 ± 0.02 °C during 3 h of operation at the highest photon flux (120 pmol s^–1^), indicating that PIAER is governed purely by photochemical processes rather than thermal effects (Figure S18).

**Figure 3:**
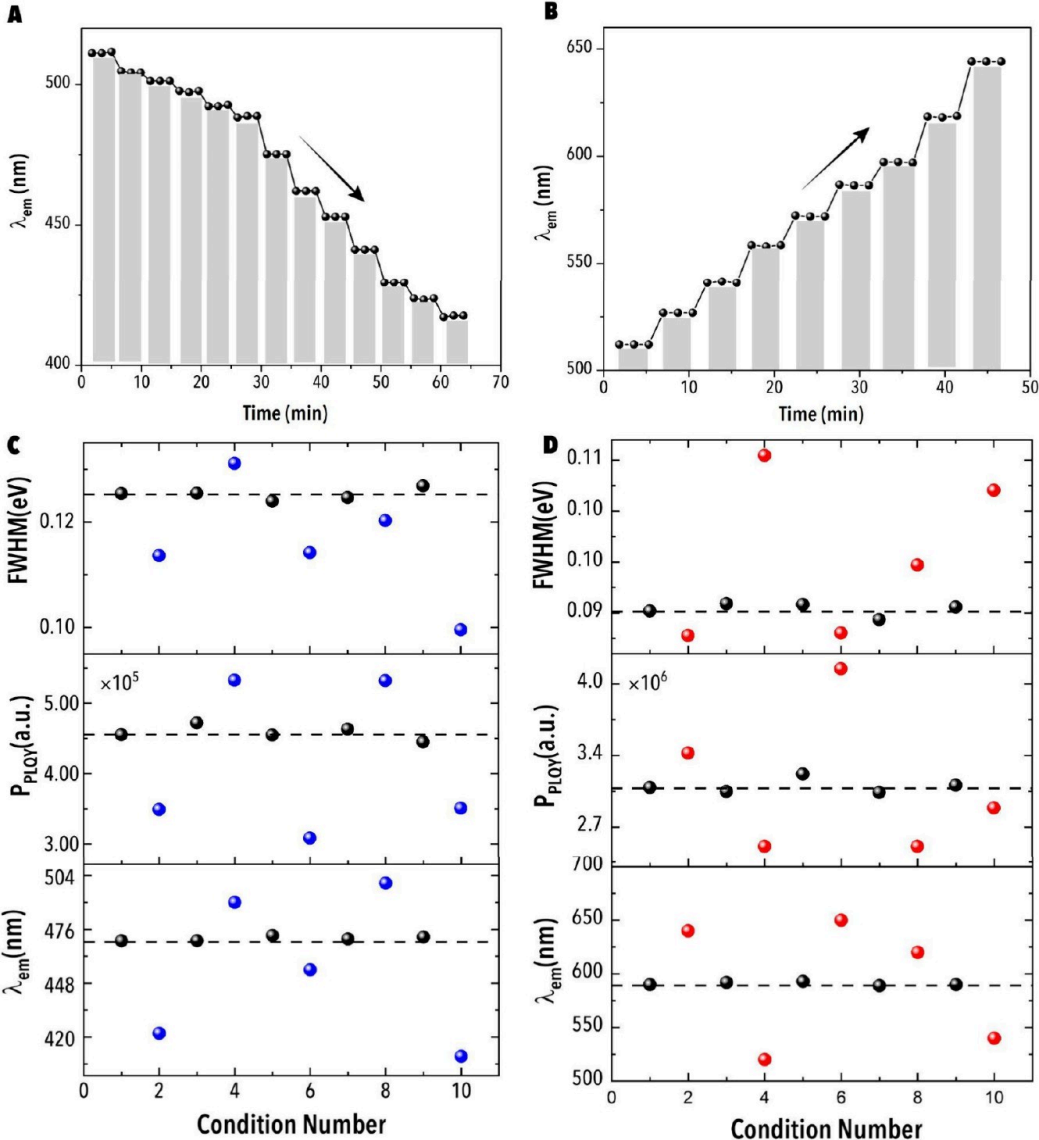
(A,B) Time-resolved evolution of λ_em_ during UV-on and UV-off experiments for blue- and red-shifts, respectively. The gray-shaded regions represent UV-off intervals. FSDL’s PIAER reproducibility study with random sampling experiments for (C, blue) chloride- (D, red) and iodide-exchange reactions. Each plot shows fwhm, *P*_PLQY_, and λ_em_ across 10 experiments with 5 repeats of the same baseline (black data points) and randomly selected (blue and red data points) PIAER conditions. Black dashed lines indicate the mean values across baseline condition replicates. Experimental conditions (baseline): [CsPbBr_3_] = 4.2 μM; [DCM] = 4.1 M; [1-iodopropane] = 0.24 M; photon flux = 15 pmol s^–1^ (DCM) and 55 pmol s^–1^ (1-iodopropane); residence time = 2.0 min; *T* = 23 °C.

To evaluate the reproducibility of the autonomous synthesis platform, we implemented a structured sequence of ten experiments consisting of five interleaved repetitions of a fixed synthesis condition and five randomized conditions. The fixed (baseline) condition, designed to produce either blue- or red-emitting CsPb­(Br/Cl/I)_3_ NCs, served as a temporal benchmark to assess experimental consistency throughout the testing sequence. Following each experiment, the microfluidic platform and all fluidic lines underwent a standardized cleaning protocol involving sequential flushing with dimethylformamide (DMF), toluene, and the relevant haloalkane, ensure the elimination of residual reagents and preserve baseline stability. Reproducibility was quantified using three *in situ* optical metrics, λ_em_, *P*_PLQY_, and fwhm. Across the five fixed-condition repetitions, the coefficients of variation remained below 1.3% for λ_em_, 1.8% for *P*_PLQY_, and 1.5% for fwhm for both emission targets (Figures 3C–D), demonstrating excellent run-to-run consistency. Importantly, no evidence of systematic drift or cross-contamination was detected, as the intervening randomized experiments exhibited no measurable impact on the fixed-condition outputs. These results confirm the FSDL’s capability to perform high-fidelity and reproducible NC synthesis under interleaved operational conditions which is an essential prerequisite for robust data acquisition in closed-loop autonomous workflows.

### Autonomous Optimization Framework and Digital Twin Modeling

To systematically explore PIAERs’ high-dimensional design space and identify synthesis conditions that yield MHP NCs with targeted λ_em_, narrow fwhm, and maximum *P*_PLQY_, a bespoke multiobjective BO framework was implemented within the FSDL. This framework utilizes Gaussian Process regression (GPR) models as surrogate predictors to construct a probabilistic map between four experimental input variables (photon flux, CsPbBr_3_ NC concentration, haloalkane volume, and reaction time), and the three corresponding optical outputs.

The GPR surrogate provides both the mean prediction and its associated uncertainty at each point in the input space, facilitating efficient and data-conscious navigation of the multiobjective landscape. Optimization is governed by the noisy expected hypervolume improvement (NEHVI) acquisition function, which is tailored to maximize *P*_PLQY_, minimize fwhm, and converge on a user-defined target λ_em_. To ensure that the ML-guided optimization remains localized to the desired spectral regime, a spectral truncation criterion is introduced. Predicted emission values falling within ± 2 nm of the target λ_em_ are retained and prioritized, while those outside this window are assigned zero acquisition gain. This approach enforces spectral specificity and ensures convergence toward emission-targeted NC synthesis conditions.

Each iteration of the BO loop involves two optimization stages. First, Sobol sequences are used to generate a quasi-random, low-discrepancy sampling of 1024 candidate experimental conditions across the bounded, multidimensional parameter space. These candidate points are then evaluated using the NEHVI acquisition function, and the optimal condition is selected via gradient-based refinement using the Limited-memory Broyden–Fletcher–Goldfarb–Shanno algorithm with Box constraints, which is well-suited for high-dimensional optimization under explicit variable bounds. The condition yielding the highest NEHVI score is selected for execution in the following NCs synthesis iteration.

A schematic of the autonomous optimization workflow is presented in Figure 4. The workflow initiates with Latin Hypercube Sampling (LHS), which provides a uniform initial coverage of the input space and supplies a diverse data set for first-round surrogate model training. Each experiment is carried out in a single-droplet microfluidic photoreactor, where reaction mixtures containing CsPbBr_3_ NCs and haloalkanes are irradiated with UV light. In-line optical characterization enables simultaneous extraction of λ_em_, *P*_PLQY_, and fwhm, which are computed through automated spectral data processing (Equations S1–S9). The new experimental data are then used to retrain the GPR surrogate models, which are then queried via NEHVI to propose the next optimal experiment to be conducted automatically by the FSDL’s hardware. This closed-loop cycle, spanning predictive data-driven modeling, acquisition-driven sampling, and experimental validation, enables rapid convergence toward Pareto-optimal NC synthesis protocols in a data-efficient manner.

**Figure 4:**
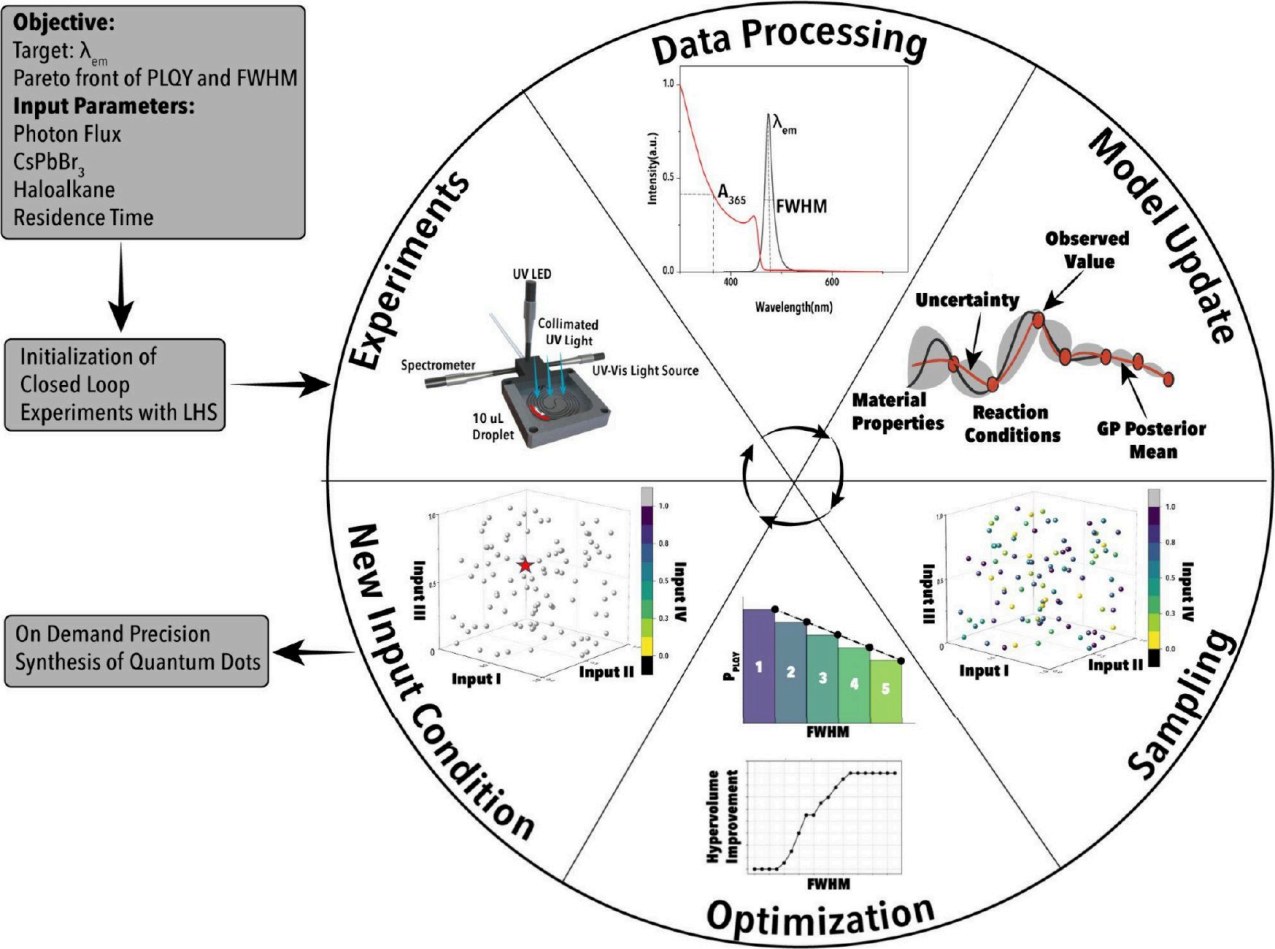
Schematic overview of the closed-loop autonomous experimentation framework for programmable synthesis of high-performing MHP NCs via PIAERs. The multiobjective BO loop is initiated by an LHS strategy to generate a diverse and unbiased set of initial experiments for training the surrogate ML model. These experiments are executed using a single-droplet microfluidic photoreactor. In situ optical characterization captures the resulting UV/vis absorption and PL spectra, from which key photophysical metrics are extracted via automated data processing. The surrogate ML model, based on Gaussian Process regression (GPR), is then retrained with the newly acquired data, updating its predictions and uncertainty estimates over the high-dimensional parameter space. Sampling strategies guided by acquisition functions (e.g., Expected Hypervolume Improvement) are used to explore the updated surrogate landscape and identify promising regions near the Pareto front. A decision policy selects the next experiment by balancing exploration and exploitation, leading to the generation of new input conditions that are passed to the FSDL’s hardware for execution. This iterative loop continues until convergence on optimal PIAER conditions for targeted optical outputs, enabling on-demand, material-efficient synthesis of compositionally tuned MHP NCs.

To support surrogate model construction and interpretability, a data-driven digital twin was developed using independently trained GPR models for each output variable. Prior to training, input and output variables were normalized and outlier data points were excluded based on a *z*-score threshold of |*z*| > 3 to improve model stability. The digital twin architecture comprises a model list, with each model independently predicting one of the three output variables, thereby preserving both interpretability and statistical independence across outputs. The digital twin architecture comprises a model list, with each model independently predicting one of the three output variables, thereby preserving both interpretability and statistical independence across outputs. The digital twin model performance was assessed via train-test splits, and prediction accuracy was quantified using the coefficient of determination (*R* ^2^), with values consistently exceeding 0.95 across all outputs (Figure 5A–C, J–L). *In silico* digital-twin simulations demonstrated rapid BO convergence, with hypervolume growth plateauing within ∼35 iterations at λ_em_ = 405 and 570 nm, confirming the robustness of the digital-twin-guided optimization framework (Figure S15).

**Figure 5:**
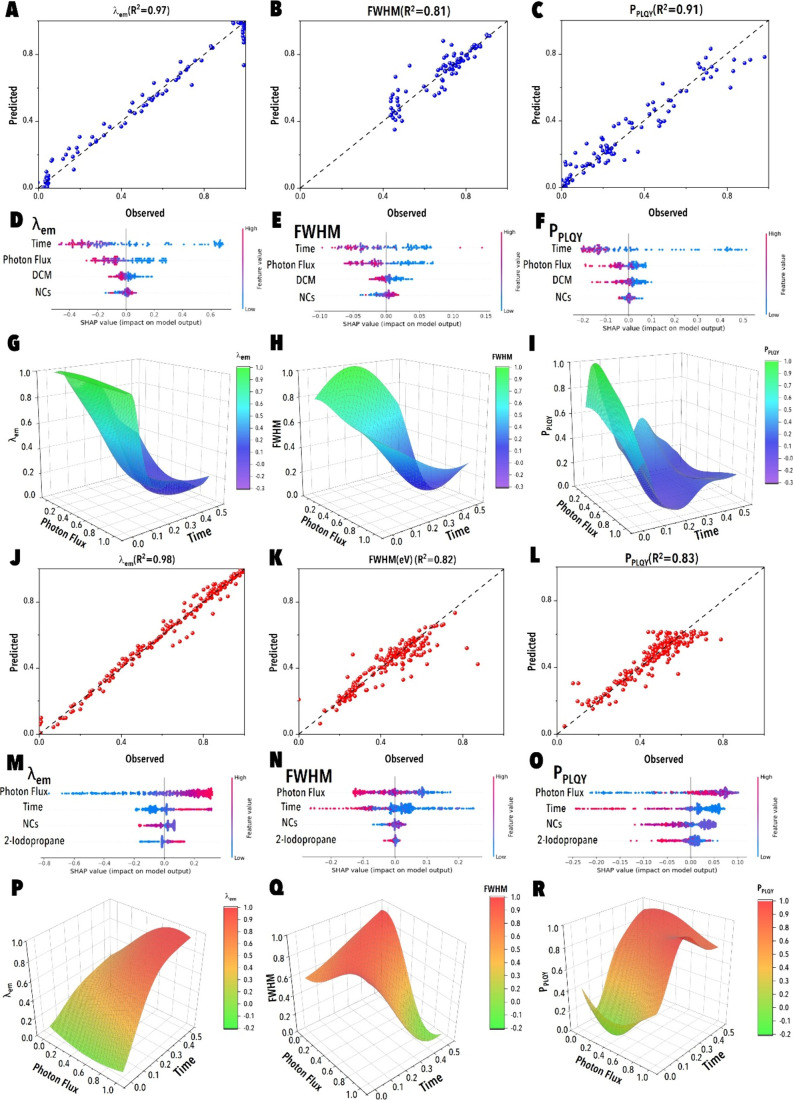
Model parity, SHAP analysis, and data-driven digital twin-predicted surface maps for PIAER systems. (A–C) The digital twin model parity plot for Br→Cl exchange using DCM as the haloalkane. (J–L) Corresponding digital twin parity plots for the 2-iodopropane-mediated Br→I exchange. SHAP analysis for input parameters’ contribution to the model prediction of (D) λ_em_, (E) fwhm, and (F) *P*_PLQY_ for Br→Cl exchange. SHAP analysis for input parameters’ contribution to the model prediction of (M) λ_em_, (N) fwhm, and (O) *P*_PLQY_ for Br→I exchange. (G–I) Digital twin-predicted λ_em_, fwhm, and *P*_PLQY_ surfaces for Br→Cl as functions of photon flux and reaction time. (P–R) Digital twin-predicted λ_em_, fwhm, and *P*_PLQY_ surfaces for Br→I as functions of photon flux and reaction time at a fixed normalized halide precursor concentration of 0.5 and NC concentration of 0.8.

Upon training with the initial 17 LHS experiments, the digital twin was deployed to conduct *in silico* evaluations over a dense input grid spanning the entire design space. The resulting response surfaces enabled detailed visualization of input–output relationships and identification of regions corresponding to optimal trade-offs among competing objectives. To further quantify the relative influence of each input parameter on the model predictions, a Shapley Additive Explanations (SHAP) analysis was conducted. This interpretability analysis revealed that photon flux and NC concentration exerted the highest influence on λ_em_ and *P*_PLQY_. In contrast haloalkane volume and reaction time played more prominent roles in modulating fwhm. These insights, derived from the data-driven digital twin, enhance mechanistic understanding of PIAERs and contribute to explainable and reproducible synthesis within the FSDL.

### Mechanistic Insights from SHAP Analysis and Response Surface Modeling

#### Br^–^→Cl^–^ Exchange Regime

SHAP analysis of the Br^–^→Cl^–^ system (Figures 5D–F) identified reaction time as the principal governing factor for both emission wavelength and emission line width, while photon flux played a secondary yet complementary role. For λ_em_, the highest SHAP values were associated with extended reaction times, consistent with more complete substitution of bromide by chloride and corresponding blue-shifts in emission (Figure 5D). Photon flux modulated the exchange kinetics at intermediate times, accelerating halide incorporation; however, its influence diminished near reaction completion, as the system approached compositional saturation.

In the case of fwhm, reaction time again emerged as the dominant feature, showing a strong negative correlation, where longer irradiation durations promoted halide homogeneity within the NC lattice, reducing the presence of mixed-halide domains and yielding spectrally narrower emission profiles (Figure 5E). *P*_PLQY_, in contrast, was most sensitive to photon flux, with SHAP values peaking at moderate to high intensities. This behavior reflects the dual role of photon flux where at optimal levels, UV irradiation promotes halide release and passivation of surface trap states, enhancing radiative efficiency; however, excessively high flux leads to a modest *P*_PLQY_ decline, attributed to photodegradation and potential ligand desorption at elevated carrier densities (Figure 5F).^36–38^

These trends are corroborated by the corresponding response surface maps (Figures 5G–I). The emission wavelength decreased monotonically with increasing reaction time, with the effect plateauing at high photon flux, indicating saturation in chloride incorporation (Figure 5G). fwhm displayed a distinct valley at long reaction times and moderate photon flux (Figure 5H), pinpointing conditions that yield optimal spectral purity through uniform halide distribution. The *P*_PLQY_ surface exhibited a broad maximum across midto-long reaction times and intermediate-to-high photon flux (Figure 5I), delineating a region of balanced reaction kinetics and minimal photodegradation.

Experiments conducted at lower NC concentrations (0.2 mM and 0.5 mM) revealed similar dependencies (Figure S4), albeit with modified kinetics. At 0.2 mM NC concentration, slower halide exchange and narrower emission line widths were observed due to reduced interparticle interactions and lower halide demand. At 0.5 mM NC concentration, faster exchange kinetics were achieved, albeit at the cost of increased emission line width variability, indicating a trade-off between throughput and compositional uniformity. Collectively, these results define a reaction-time–dominated exchange regime, in which extended reaction duration governs the extent of substitution and emission purity, while photon flux primarily modulates the photophysical parameters.

#### Br^–^→I^–^ Exchange Regime

In contrast to the Br^–^→Cl^–^ exchange reactions, SHAP analysis of the Br^–^→I^–^ exchange (Figures 5M–O) revealed a photon-flux–dominated regime, with flux exerting the greatest influence on both λ_em_ and *P*_PLQY_, while reaction time had the strongest effect on fwhm. For λ_em_, increasing photon flux produced large positive SHAP contributions corresponding to red-shifted emission, driven by rapid iodide incorporation and bandgap narrowing. These effects were amplified at longer reaction times, which allowed more extensive halide substitution and lattice integration of iodide ions (Figure 5M).

The fwhm exhibited a distinct dependence on reaction time, which emerged as the top-ranked feature for spectral broadening (Figure 5N). Extended irradiation durations correlated positively with emission line width, indicating increased compositional heterogeneity and potential phase segregation in iodide-rich domains. Photon flux also contributed to the emission line width broadening at high levels, likely due to oversubstitution and the emergence of multiphase or defect-prone NCs.

*P*_PLQY_ displayed a nonmonotonic dependence on photon flux, with SHAP values peaking at intermediate-to-high intensities and declining sharply at the upper flux range (Figure 5O). This behavior reflects the kinetic competition between beneficial iodide incorporation, which is enhanced under moderate flux, and photoinstability effects at high flux. Specifically, at moderate flux, efficient halide exchange coupled with thiol-mediated surface passivation improved radiative efficiency.^8,16,39,40^ However, excessive photon flux induced the desorption of passivating ligands and promoted the formation of iodide-rich domains prone to static disorder and nonradiative recombination, thereby diminishing the *P*_PLQY_.^41,42^ These mechanistic trade-offs are visualized in the *P*_PLQY_ response surface (Figure 5R), which features a sharp ridge along the moderate–high flux and short-to-intermediate reaction time region, representing the optimal balance between rapid exchange and photostability.^8,43^

The response surface plots further validate the above-mentioned trends: λ_em_ increased steeply with photon flux across all reaction times, with the largest gradients observed at long reaction times where substitution was most complete (Figure 5P). fwhm broadened significantly with increasing reaction time, with narrow line widths confined to the regime of moderate flux and shorter irradiation times (Figure 5Q). *P*_PLQY_ exhibited a ridge-like maximum under moderate flux and controlled reaction time, consistent with conditions that maximize iodide incorporation while minimizing NCs degradation (Figure 5R).

At lower NC concentrations (0.2 mM and 0.5 mM), the same flux-dependent trends were observed, albeit with concentration-modulated kinetics (Figure S5). At NC concertation of 0.2 mM, the progression of the red shift was more gradual, and *P*_PLQY_ peaked at intermediate conditions, with fwhm displaying a broad maximum indicative of temporal instability. Increasing the NC concentration to 0.5 mM accelerated iodide incorporation but also led to broader emission line widths and a more pronounced decline in *P*_PLQY_ under high flux and prolonged irradiation.

These findings contrast sharply with the Br^–^→Cl^–^ regime, emphasizing the mechanistic distinctions between chloride and iodide exchange. Chloride substitution is favored by extended reaction times, benefiting from slow but uniform incorporation, whereas iodide exchange is predominantly governed by photon flux and limited by the reduced photostability of iodide-rich NCs.^8,19,44,45^ This mechanistic divergence establishes a foundation for reaction-specific design strategies, where kinetic and optical outcomes are tailored through precise control of reaction time and photon flux, depending on the target halide composition.

#### Autonomous PIAER Campaigns

Autonomous experimental campaigns were conducted to systematically explore photoinduced halide exchange in CsPbBr_3_ NCs, targeting both Br^–^→Cl^–^ substitution for blue-shifted emission and Br^–^→I^–^ substitution for red-shifted emission. DCM and 2-iodopropane served as photochemically activated halide donors, releasing Cl• and I• radicals under UV irradiation. Each campaign began with 5 LHS experiments to provide unbiased initial data for the surrogate model training, followed by fewer than 30 BO iterations to converge on the NC synthesis conditions resulting in high *P*_PLQY_ and narrow fwhm for a targeted λ_em_ within ± 4 nm (Supporting Information S6 **)**. The optimization variables included haloalkane concentration, photon flux, and NC concentration, with reaction time adjusted in the microfluidic photoreactor. Optical metrics were monitored *in situ* and processed in real time, enabling rapid closed-loop decision-making.

In the Br^–^→Cl^–^ system, BO trajectories consistently revealed a reaction-time–dominated regime. Initial LHS experiments produced broad line widths (fwhm ≥ 0.12 eV) and low *P*_PLQY_ (Figures 6A-C), consistent with incomplete substitutions. As BO iterations advanced, the optimization narrowed to a regime of moderate DCM fractions (≈0.4–0.6), intermediate NC loadings (≈0.3–0.5), elevated photon flux (≥0.6), and short-to-intermediate reaction times (∼1–2 min). These conditions reproducibly yielded narrower emission line widths (≈0.10–0.11 eV) and higher *P*_PLQY_, reflected in the sharp hypervolume gain observed around experiment 15 (Figure 6B). The Pareto-front (Figure 6C) shows clustered high-performing solutions at fwhm ≈ 0.120–0.125 eV near λ_em_ = 480 nm, precisely where the Br^–^→Cl^–^ digital twin predicted overlapping maxima in *P*_PLQY_ and minima in fwhm. At deeper blue emission targets, such as λ_em_ = 460 and 405 nm, BO trajectories (Figures 6D-F and 6G-I) funneled into progressively narrower regions of parameter space. The hypervolume traces (Figures 6E and 6H) exhibit stepwise increases followed by early plateaus, reflecting the increasingly stringent kinetic and structural constraints imposed by near-complete substitution. The resulting Pareto-fronts (Figures 6F, 6I) highlight the trade-offs between NC emission purity and radiative efficiency; while narrower emission line widths were consistently obtained at longer reaction times, *P*_PLQY_ could only be maintained within confined flux windows. These results confirm the time-led substitution mechanism predicted by SHAP analysis (Figures 5D–F, 5G–I), where reaction time dictates spectral homogeneity, and photon flux fine-tunes efficiency by modulating Cl• generation.

**Figure 6:**
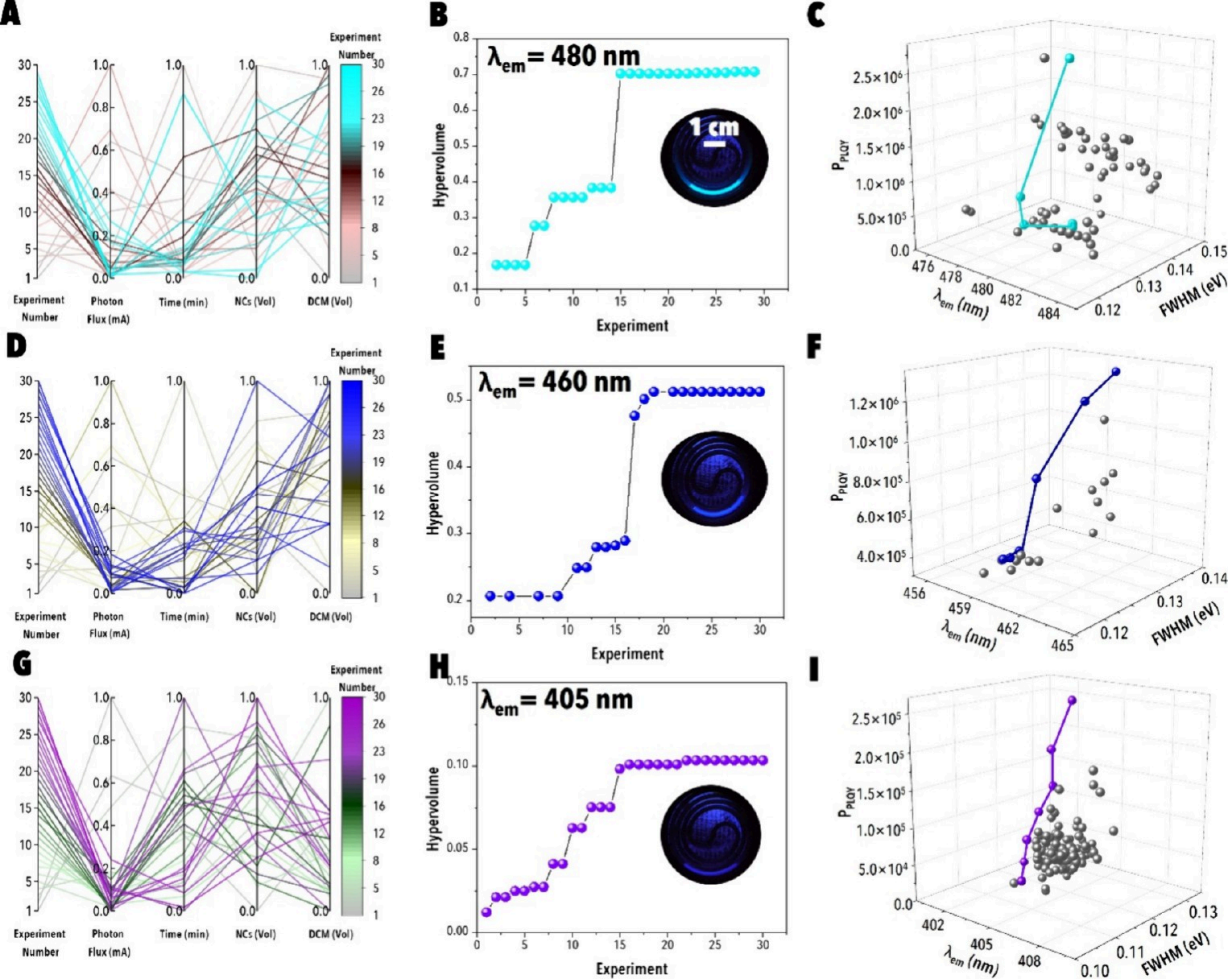
Autonomous PIAER for blue-emitting CsPb­(Br/Cl)_3_ NCs at varying target emission wavelengths. Autonomous PIAER route discovery for target λ_em_ of (A–C) 480 nm, (D–F) 460 nm, (G–I) 405 nm. (A, D, G) Parallel coordinate plots of normalized input variables (photon flux, reaction time, NCs concentration, and DCM fraction) across experimental campaigns. (B, E, H) Evolution of the hypervolume metric as a function of experiment number, with insets showing representative emission of optimized NCs under UV illumination. (C, F, I) Pareto-front mapping of *P*_PLQY_ vs emission wavelength and fwhm, highlighting optimal trade-offs achieved during each campaign.

In contrast, the Br^–^→I^–^ campaigns demonstrated a photon flux-dominated regime consistent with SHAP analysis (Figures 5M–O). Early LHS experiments (Figures 7A, 7D, and 7G) yielded broad emission (fwhm ≥ 0.20 eV) and poor *P*_PLQY_, reflecting unstable mixed-halide states. BO progressively concentrated on regimes of intermediate-to-high photon flux (≥0.5–0.6), moderate iodopropane fractions (≈0.4–0.6), and NC loadings of 0.3–0.5. For λ_em_ = 570 nm, the hypervolume trace (Figure 7B) showed a pronounced increase near experiment 12, marking the discovery of conditions that balanced rapid iodide incorporation with surface stabilization. The corresponding Pareto-front (Figure 7C) revealed high *P*_PLQY_ values and emission line widths of ∼0.12–0.15 eV. At λ_em_ = 600 nm, convergence shifted to high photon flux and extended reaction times (Figure 7D), with hypervolume growth (Figure 7E) and Pareto distributions (Figure 7F) indicating broader emission line widths (∼0.14–0.16 eV), consistent with digital twin predictions that reaction time positively correlates with fwhm through compositional heterogeneity. At λ_em_ = 650 nm, BO identified a narrow stability window defined by long irradiation (>3 min) and high flux (>0.6), with the hypervolume trace plateauing after ∼18 experiments (Figure 7H). The Pareto-front (Figure 7I) demonstrates narrowed emission line widths (0.10–0.12 eV) and high *P*_PLQY_, confirming that flux-driven iodide incorporation can be stabilized when reaction time and precursor loading are finely balanced. Together, these results validate the mechanistic divergence between chloride and iodide exchange: Cl^–^ substitution benefits from extended time-dependent exchange, while I^–^ incorporation is flux-dominated and limited by the photoinstability of iodide-rich domains.

**Figure 7:**
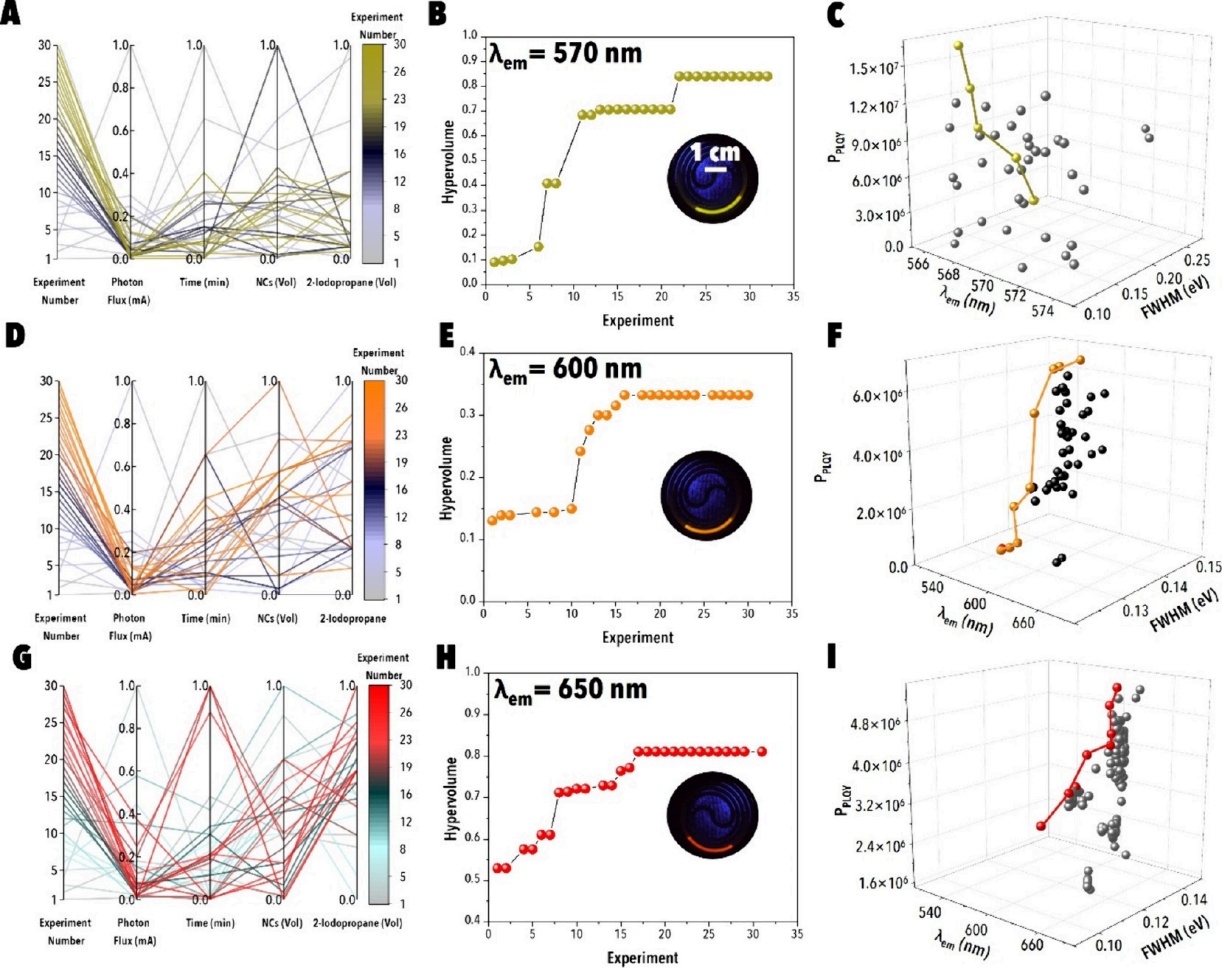
Autonomous optimization of PIAER for red-emitting CsPb­(Br/I)_3_ NCs at varying target emission wavelengths. Autonomous PIAER route discovery for target λ_em_ of (A–C) 570 nm, (D–F) 600 nm, (G–I) 650 nm. (A, D, G) Parallel coordinate plots of normalized input variables (photon flux, reaction time, NCs concentration, and 2-iodopropane fraction; all scaled 0–1) across experimental campaigns. (B, E, H) Evolution of the hypervolume metric as a function of experiment number, with insets showing representative droplet images of optimized NCs under UV illumination. (C, F, I) Pareto-front mapping of *P*_PLQY_ vs emission wavelength and fwhm, highlighting optimal trade-offs achieved during each campaign.

Across all campaigns, photon flux emerged as the critical kinetic parameter, directly controlling the rate of haloalkane photolysis and radical generation. Optimal precursor stoichiometry (Tables S2–S8) consistently improved NC stability by mitigating halide vacancy formation, while NC concentration balanced surface passivation with reaction kinetics. The BO trajectories, hypervolume trends, and Pareto-fronts (Figures 6 and 7) collectively demonstrate that closed-loop optimization can efficiently map synthesis–property landscapes, converging on optimal emission targets within 30 experiments. Comparative concentration-dependent studies (Figures S4–S5) further confirmed that lower NC concentrations slowed exchange and narrowed emission line widths, whereas higher concentrations accelerated substitution at the expense of spectral uniformity.

#### Knowledge Scalability

To test whether synthesis knowledge from the single droplet-scale FSDL could be transferred directly to larger scales, the optimal reaction conditions identified during BO campaigns were implemented in a continuous-flow photoreactor operating at throughputs of 50–250 mL·day^–1^. Without further optimization, the continuous flow system reproduced the emission wavelength, line width, and *P*_PLQY_ values discovered at the single droplet scale (Figure 8A). Each continuous flow run was maintained for ∼60 min, and absolute *P*_PLQY_ was quantified using a benchtop spectrofluorometer equipped with an integrating sphere (Figure S8). Replicate experiments confirmed reproducibility and eliminated reactor-dependent artifacts (Figures S6–S8, Tables S1,9–10).

**Figure 8:**
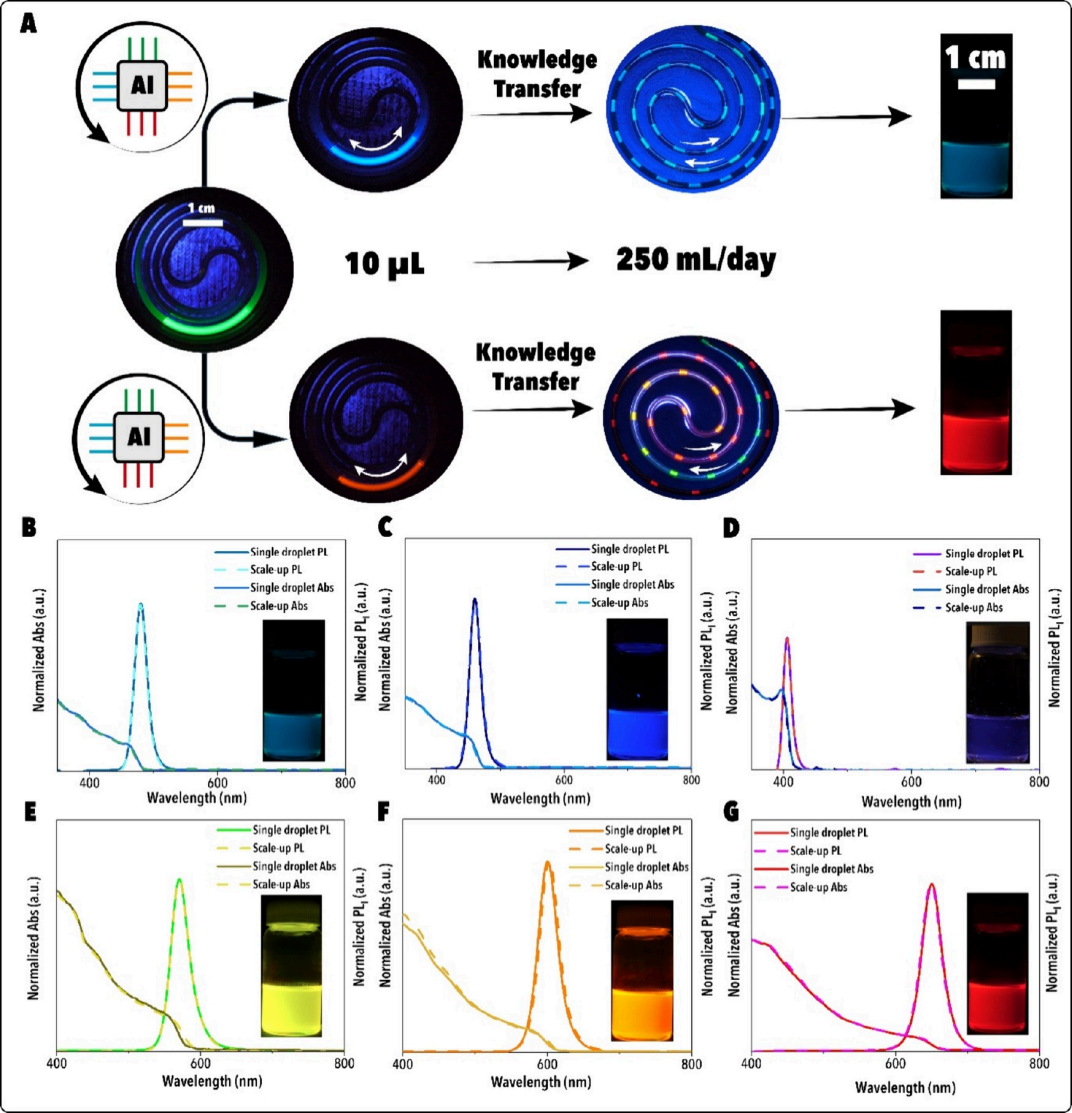
(A) Knowledge transfer from single-droplet optimization (10 μL scale) to continuous flow synthesis (250 mL/day) under UV illumination. Representative microfluidic reactor images display droplet-scale experiments and corresponding scale-up flow reactors, featuring final MHP NC products under UV illumination. Corresponding UV–vis absorption and PL spectra for champion MHP NCs at (B) λ_em_ = 480 nm, (C) λ_em_ = 460 nm, (D) λ_em_ = 405 nm, (E) λ_em_ = 570 nm, (F) λ_em_ = 600 nm, and (G) λ_em_ = 650 nm. Insets present scale-up NC products under UV illumination (Video S2).

*Ex situ* characterization corroborated the optical properties with structural and compositional evidence. X-ray diffraction (XRD) confirmed retention of the cubic perovskite structure across all compositions, with no secondary phases detectable above the 2 wt % threshold (Figures S9–S10). High-resolution transmission electron microscopy (HR-TEM) of the (200) planes revealed systematic lattice expansion with increasing halide ionic radius: *a* = 5.60 Å for CsPb­(Br/Cl)_3_ NCs (λ_em_ = 405 nm, *d*_200_ = 0.28 nm), *a* = 5.80 Å for CsPbBr_3_ NCs (λ_em_ = 510 nm, *d*_200_ = 0.29 nm), and *a* = 6.40 Å for CsPb­(Br/I)_3_ NCs (λ_em_ = 650 nm, *d*_200_ = 0.32 nm) (Figure S11). These values agree with literature reports for CsPbCl_3_, CsPbBr_3_, and CsPbI_3_.^8,36,46,47^ Statistical TEM size-distribution analyses (Figure S17) reveal a modest, monotonic increase in mean edge length from Cl- to I-rich compositions, which is consistent with the progressive lattice expansion evidenced by XRD peak shifts (Figures S9–S10) and HRTEM (200) plane imaging (Figure S11), driven by the increasing halide ionic radius (Cl^–^ < Br^–^ < I^–^). These small variations (<10%) confirm that the observed spectral shifts arise primarily from composition-driven bandgap tuning rather than size-dependent quantum confinement. Energy-dispersive X-ray spectroscopy (Tables S2–S8) independently verified halide compositions, with Cl fractions increasing from *x*_Cl_ = 0.12 at λ_em_ = 480 nm to 0.89 at 405 nm, and iodide fractions increasing from *x*_I_ = 0.38 at 570 nm to 0.87 at 650 nm.

Together, Figures S9–S11 confirm that the structure–composition–property relationships uncovered during single droplet-scale optimization were preserved across a four-order-of-magnitude scale-up. These results establish knowledge scalability as a central principle of autonomous NC synthesis, where reaction conditions discovered in microliter volumes can be directly applied to continuous-flow production of compositionally tunable CsPbX_3_ (X=Cl, Br, I) NCs without reoptimization.

## Conclusions

This study presents the first programmable and scalable platform for light-directed bandgap engineering of MHP NCs via PIAERs. By integrating a droplet-based microfluidic photoreactor with real-time optical diagnostics and multiobjective BO, the developed FSDL autonomously identified optimal synthesis conditions across a multidimensional parameter space. The FSDL consistently delivered high-performance NCs with target emission wavelengths, elevated photoluminescence quantum yields, and narrow emission line widths using fewer than 30 experiments per emission target.

Surrogate model–guided analysis delineated distinct mechanistic regimes for halide exchange; Br^–^→Cl^–^ substitution was primarily governed by reaction time, enabling compositional homogeneity and spectral narrowing, whereas Br^–^→I^–^ incorporation was driven by photon flux, with reaction time acting as a secondary control over stability and phase purity. These mechanistic fingerprints enabled reaction-specific optimization, underscoring the tunability of PIAER kinetics as a rational design tool.

Crucially, optimal MHP NC synthetic routes discovered in a 10 μL droplet format were directly scaled to a continuous-flow operation producing 50–250 mL·day^–1^ of NC dispersion without loss of optical performance. This direct transfer of synthesis knowledge across discovery and manufacturing scales, without empirical retuning, validates knowledge scalability as a core engineering principle for autonomous materials synthesis.

Together, these findings establish a generalizable and sustainable approach for scalable photochemical synthesis of compositionally tunable perovskite NCs. By resolving complex trade-offs between competing optical properties and uncovering regime-specific PIAER behavior, this work provides both a mechanistic and operational foundation for the deployment of light-driven, autonomous synthesis platforms in nanomaterials manufacturing.

## Method and Materials

### Chemicals

Cesium carbonate (Cs_2_CO_3_, 99%, ReagentPlus), lead­(II) bromide (PbBr_2_, ≥98%), oleic acid (OA, 90%, technical grade), oleylamine (OAm, 70%, technical grade), 1-octadecene (ODE, 90%, technical grade), dichloromethane (DCM, ≥99.8%, anhydrous), N,N-dimethylformamide (DMF, 99.8%, anhydrous), toluene (99.8%, anhydrous), 2-iodopropane, and 1-dodecanethiol were purchased from Sigma–Aldrich. Methyl acetate (99%, extra pure) was obtained from Acros Organics. All chemicals were used without further purification.

### Preparation of Cesium Oleate (Cs-OA) Stock Solution

Cesium oleate precursor was prepared by combining cesium carbonate (Cs_2_CO_3_, 101.7 mg) with 5 mL of 1-octadecene (ODE) and 325 μL of oleic acid (OA) in a 20 mL septum-sealed glass vial equipped with a magnetic stir bar. The mixture was placed under vacuum and stirred at 120 °C for 30 min to remove volatile impurities and promote precursor dissolution. Following degassing, the temperature was increased to 150 °C under a continuous nitrogen (N_2_) atmosphere for an additional 10 min. The cesium oleate stock solution was maintained at this temperature until required for CsPbBr_3_ NC synthesis (Figure S12).

### Lead Bromide (PbBr_2_) Precursor Solution

For the lead halide precursor, lead­(II) bromide (PbBr_2_, 138 mg) was mixed with 1 mL each of OAm and OA, along with 10 mL of ODE in a separate 20 mL septum vial. This mixture was degassed under vacuum at 120 °C for 30 min to eliminate residual moisture and oxygen. The solution was then heated to 180 °C under N_2_ purge for 10 min to fully dissolve the PbBr_2_ and activate the complexation with ligands. The lead bromide precursor solution was held at this elevated temperature in preparation for immediate injection of the cesium source.

### Synthesis of CsPbBr_3_ NCs

When both Cs-OA and PbBr_2_ precursors reached their target temperatures—150 °C for the Cs-OA and 180 °C for the PbBr_2_ solution—an aliquot of 1.2 mL Cs-OA was swiftly injected into the lead precursor vial to initiate the NC formation. The reaction proceeded for 5 s before being quenched by immersing the entire vial in an ice–water bath to halt NC growth and preserve a narrow size distribution.

### Purification of CsPbBr_3_ NCs

The crude product (2.5 mL) was transferred into four 14 mL centrifuge tubes, each receiving an equal volume of methyl acetate (2.5 mL), resulting in a 1:1 solvent-to-crude volume ratio. After thorough mixing, samples were centrifuged at 8,500 rpm for 6 min. The supernatant was discarded, and the resulting precipitate in each tube was redispersed in 3 mL of toluene. A second purification step was performed by centrifuging the redispersed solution at 8,000 rpm for 5 min. The absorbance of the final product (purified CsPbBr_3_ NCs) was adjusted via dilution with toluene to 1 *a.u*. at 356 nm for subsequent PIAER experiments.

### FSDL Hardware

#### Discovery Mode Hardware (Single-Droplet Microfluidic Photoreactor)

The self-driving microfluidic system developed and utilized for the data-driven PIAER exploration and optimization consists of a custom-built UV-transparent microfluidic photoflow reactor. The microreactor was fabricated using fluorinated ethylene propylene (FEP) tubing (internal diameter, ID: 0.762 mm; outer diameter, OD: 1.5875 mm; total reactor volume: 180 μL), illuminated with a high-intensity 365 nm UV LED source (Thorlabs SOLIS-365C) with tunable output controlled by a DC2200 driver. The tubing was housed within a CNC-machined aluminum enclosure that ensures consistent light distribution and thermal stability. A secondary FEP flow path (ID: 0.508 mm, OD: 1.5875 mm) was used for reagent mixing and transport.

The microreactor’s inlet and outlet interfaces were secured in a custom-designed, 3D-printed black PLA flow cell that allowed multimodal *in situ* optical monitoring. This flow cell housed a fiber-coupled spectrometer (HDX, Ocean Optics), a broadband light source (DH-2000-BAL, Ocean Optics), and a UV excitation module (Thorlabs M365LP1), connected via fiber optic patch cords (QP-600–1-SR, Ocean Optics). All reagent injections were controlled via computer-operated syringe pumps (Fusion 200, Chemyx) connected to 180 μL and 5 mL precision glass syringes (SGE) and routed through a T-junction (IDEX PEEK) for droplet generation.

The single-droplet microfluidic platform was operated under positive N_2_ pressure (138 kPa) supplied via two pressure vessels (Airgas NI UHP300), which facilitated stable flow and droplet motion. Each reaction droplet was actuated at a steady volumetric flow rate of 300 μL·min^–1^ and oscillated within the illuminated region at 1.1 cm·s^–1^ using a syringe-free pump (VICI M-6 series). Following the PIAER, a multistep automated microreactor rinse cycle was initiated, alternating between DMF, toluene, and DCM to clean the fluidic path. Each wash droplet was driven by N_2_ as the carrier gas to prevent residual accumulation between PIAER runs.

The entire PIAER process was controlled by a custom LabVIEW interface that coordinated pump actuation, light modulation, and spectroscopic data acquisition (Figures S13–14). The UV–vis absorption and PL spectra were processed using a Python script that automatically extracted peak positions, *P*_PLQY_, and fwhm. Input parameters—photon flux (mA), haloalkane content (Vol%), and NC volume fraction (Vol%) as well as reaction time—were loaded from structured Excel files to facilitate seamless coordination with the multiobjective BO algorithm. An auxiliary refill module ensured uninterrupted replenishment of toluene and haloalkane syringes, supporting long-term autonomous operation.

#### Continuous Manufacturing Mode Hardware (Continuous Droplet-Flow Microreactor)

The continuous-manufacturing configuration of the FSDL utilizes exactly the same UV-transparent FEP microreactor, optical enclosure, and 365 nm LED illumination module described for the discovery mode; no dimensional or photonic modifications were introduced. Instead of a single oscillating droplet,, the microreactor operates as a continuous gas–liquid segmented flow, in which discrete reaction slugs of precursor solution (≈ approximately 4 μL each) are separated by equally sized N_2_ segments produced at 138 kPa from the existing pressure manifold. The slug train is generated at a PEEK T-junction, where the mixed PIAER precursor streams merge immediately upstream of the junction before meeting the continuous N_2_ phase. The same fiber-coupled UV–vis absorption and PL probes used in the discovery mode are positioned 15 cm downstream of the light zone of the microreactor. Spectra are processed in real-time by the existing LabVIEW/Python supervisory loop; deviations of the PL peak position by > ± 2 nm automatically trigger closed-loop adjustments to precursor pump rates or LED current, maintaining product specifications during campaign-scale operation.

The developed continuous flow format yields in ∼50–250 mL/day of high-performing CsPbX_3_ NC dispersion with a targeted peak emission wavelength, demonstrating seamless scale-up from single-droplet discovery to continuous manufacturing without altering the fundamental reaction conditions. Quantitative throughput, yield, reagent consumption, and energy metrics derived from these runs are reported in Table S11. Compared to other postsynthetic band gap-tuning methods, the present photon-driven microfluidic strategy offers broader tunability, higher PLQY retention, and greener continuous-flow scalability (Table S12).

#### Ex Situ Characterization

Transmission electron microscopy (TEM) and energy-dispersive X-ray spectroscopy (EDS) were performed using an FEI Talos F200X operated at 200 kV. Samples were drop-cast onto carbon-coated copper grids. UV–vis and photoluminescence measurements were carried out using an Edinburgh Instruments FS5 Spectrofluorometer. Absolute PLQY was measured using an integrating sphere. Grazing incidence X-ray diffraction (GIXRD) measurements were carried out at room temperature (298 K) using a Rigaku SmartLab diffractometer equipped with a Cu Kα radiation source (λ = 1.54 Å, 44 mA, 40 kV) and operated in a parallel beam configuration. For analysis, 1 mL of each sample was drop-cast onto a microscope glass slide and dried for 24 h under open air.

## Supplementary Material









## Data Availability

All data and custom code supporting the findings of this study are available via GitHub at https://github.com/AbolhasaniLab under the repository name ‘PIAER_Closed_Loop’.

## Notes

Design and operation of the microfluidic photoreactor for accelerated studies of photoinduced anion exchange reactions; parameter-space discretization and automated spectral data processing (eq S1–S9); proxy PLQY definition and cross-instrument calibration; photon-flux quantification; UV-on/off controls, intrinsic kinetics vs. photon flux and thermal stability of the microreactor; digital-twin visualizations and *in-silico* BO convergence with hypervolume analysis; architecture and implementation of the autonomous BO workflow; continuous microfluidic photoreactor setup; cross-scale reproducibility metrics and photon-normalized yield/energy consumption; EDS compositional mapping; TEM/HRTEM/XRD characterization; optical properties of starting CsPbBr_3_ nanocrystals; process automation interface for closed-loop operation and reagent delivery control (PDF)
Video S1: Demonstration of droplet formulation and oscillation within the UV-illuminated photoreactor for single-droplet optimization (MP4)
Video S2: Scale-up NC products under UV illumination (MP4)
Transparent Peer Review report available (PDF)
