# Supplementary material for: Programmable Light-Driven Color Tuning of Perovskite Quantum Dots
Source: ACS Cent Sci. 2025 Nov 13;11(12):2433–47. doi: 10.1021/acscentsci.5c01651 (PMC12746153; doi:10.1021/acscentsci.5c01651)
Supplement: Supplementary file 1 [file oc5c01651_si_001.pdf]

Supplementary Information

Programmable Light-Driven Color Tuning of Perovskite Quantum Dots

Pragyan Jha,[1] Nikolai Mukhin,[1] Jinge Xu,[1] Christopher H.J. Moran,[1] Arup Ghorai, [1] Felix N. Castellano,[2] and Milad Abolhasani[1]*

[1]Dept. of Chemical and Biomolecular Engineering, North Carolina State University, Raleigh, NC 27606, USA

[2]Dept. of Chemistry, North Carolina State University, Raleigh, NC 27695, USA

**Corresponding author, abolhasani@ncsu.edu*

Table of contents

S1. Microfluidic Photoreactor 3

S2. Parameter Space and Data Processing 4

S3. P_{PLQY} Validation 5

S4. Photon Flux Measurements 7

S5. PIAER Visualization 8

S6. Autonomous Decision-Making Algorithm 10

S7. Continuous Microfluidic Photoreactor 13

Table S1. *Continuous-flow PIAER operating conditions for targeted emission wavelengths (250 µL photoreactor).* 15

S8. EDS Mapping 16

Table S2. *EDS analysis of $CsPb(Br/Cl)_3$ NCs with λ_{em} = 510 nm* 16

Table S3. *EDS analysis of $CsPb(Br/Cl)_3$ NCs with λ_{em} = 480 nm* 17

Table S4. *EDS analysis of $CsPb(Br/Cl)_3$ NCs with λ_{em} = 460 nm* 17

Table S5. *EDS analysis of $CsPb(Br/Cl)_3$ NCs with λ_{em} = 405 nm* 18

Table S6. *EDS analysis of $CsPb(Br/I)_3$ NCs with λ_{em} = 570 nm* 18

Table S7. *EDS analysis of $CsPb(Br/I)_3$ NCs with λ_{em} = 600 nm* 19

Table S8. *EDS analysis of $CsPb(Br/I)_3$ NCs with λ_{em} = 650 nm* 19

S9. TEM and XRD Analysis 20

S10. Starting $CsPbBr_3$ NCs 22

S11. LabVIEW Interface for Closed-loop PIAER 23

S12. Reproducibility and Cross-Scale Consistency of $CsPbX_3$ Nanocrystals 25

Table S9. *Percent Error between Single-Droplet and Continuous-Flow $CsPbX_3$ Nanocrystals* 25

Table S10. *Coefficient of Variation (CV%) in Continuous-Flow for 30 minutes* 26

S13. In silico digital-twin convergence of Bayesian optimization. 26

S14. Kinetics and Control Experiment 27

S15. TEM Size Distribution 28

S16. Yield and Energy Consumption 29

Table S11. *Throughput and Resource Metrics of the Continuous-Flow Photoreactor* 29

S17. Post Band-gap Tuning Comparison 30

Table S12. *Comparison of post-synthetic bandgap-tuning strategies for $CsPbX_3$ nanocrystals* 30

S18. Thermal Stability of the Microfluidic Photoreactor 31

Supplementary References 32

S1. Microfluidic Photoreactor

The designed microfluidic photoreactor, shown in **Figure S1** integrates illumination, reactive droplet manipulation, and *in situ* optical monitoring. At the top, a collimated UV light source provides uniform, high-intensity irradiation across the microchannel, ensuring consistent photon flux delivery. An optical adapter aligns the light path with the flow cell housed in the CNC-machined reactor plate, minimizing losses and enabling efficient light–matter interaction. The reactor body contains a custom-designed 3D-printed flow cell connected to inlet and outlet ports, allowing precise delivery and mixing of reagents under continuous flow conditions. Three optical fibers are coupled directly to the flow cell for real-time spectroscopic measurements, enabling simultaneous monitoring of UV-Vis-NIR absorption and photoluminescence (PL) spectra of metal halide perovskite (MHP) nanocrystals (NCs) during the photo induced anion exchange reaction (PIAER) process. This integrated setup allows for accurate control over reaction conditions, efficient mixing, and reproducible exposure to UV illumination, providing a versatile platform for accelerated fundamental and applied studies of PIAER in flow.

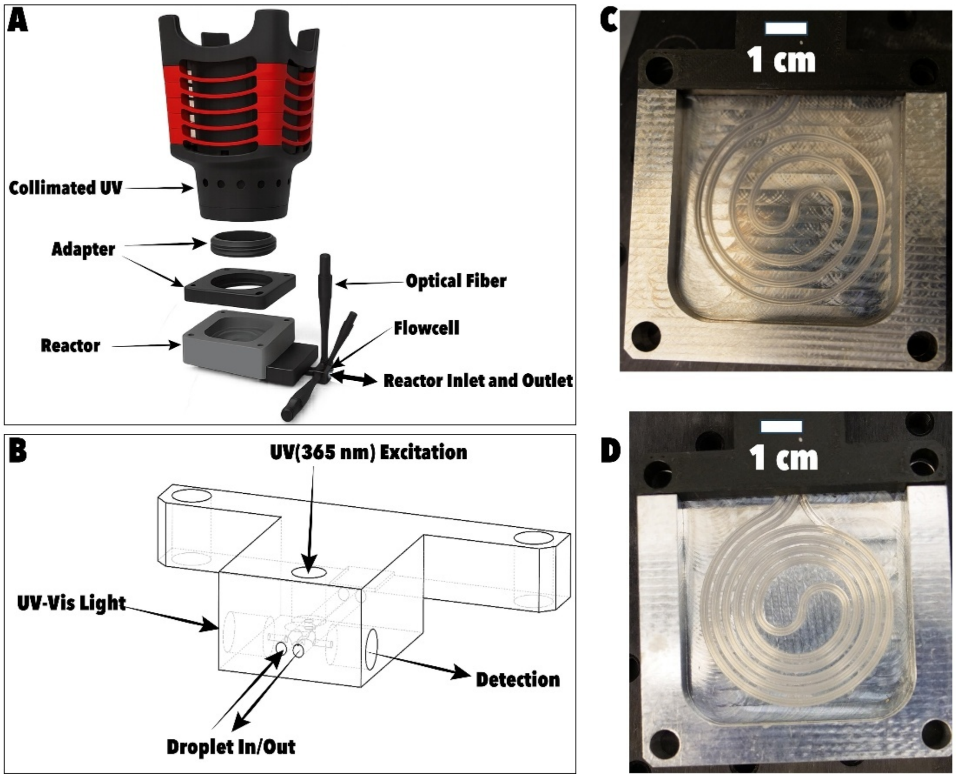

Figure S1. (A) *Exploded view of the custom-designed microfluidic photoreactor with its key components, including a collimated UV light source (365 nm), an optical adapter, and a multimodal flow cell integrated with an optical fiber for in situ spectroscopic monitoring. The reactor body accommodates the flowcell and*

connects to the reagent delivery lines via reactor inlet and outlet ports. (B) Flow cell schematic. Droplets enter/exit at the front ports; 365 nm UV excites from the top; UV–Vis light passes across the cell for absorbance; emission is collected at the side detection port. Photographs of (C) the single-droplet microfluidic photoreactor with a fluoroethylene propylene (FEP) tubing (0.03" inner diameter×1/16" outer diameter, total volume=180 μL) used for the autonomous PIAER optimization, and (D) the microfluidic photoreactor (0.03" inner diameter×1/16" outer diameter, total volume=250 μL), used for continuous flow MHP NC synthesis (knowledge transfer).

S2. Parameter Space and Data Processing

Parameter space. PIAER's parameter space studied in this work was quantified by discretizing each experimental input according to the precision of the developed microfluidic platform. With a volumetric increment of 0.12 μL per pump step, a 10 μL droplet composed of three input streams ($CsPbBr_3$ NCs, toluene diluent, and haloalkane) yields approximately 3.6×10^3 admissible compositions on the discretized simplex. Independent variation of photon flux between 0 and 10 pmol s^{-1} further enlarges the design space; even at a conservative step size of 0.1 pmol s^{-1}, the combined composition–flux space encompasses ~10^5 distinct experimental conditions. Reaction time was fixed at 120 min and excluded as a design variable. This corrected accounting captures the true dimensionality of the system, demonstrating that even within constrained operating windows, the parameter space expands combinatorially to a scale that precludes exhaustive search and necessitates closed-loop, data-driven exploration.

Automated Data Processing. Following automated *in-situ* acquisition of the UV-Vis-NIR absorption and PL spectra, raw wavelength axes for PL and absorption (λ_{PL}, λ_{Abs}), dark references for PL and absorption, and a light reference for absorption were retrieved from calibration files. The processing pipeline operated in live mode, continuously monitoring the experiment directory for newly generated datasets. Spectral files were paired by matching experiment and iteration identifiers in the filename, for spectral measurements, files containing the 'UVON' label. Each valid triplet, comprising a PL spectra (`P_SAMP*UVON.csv`), absorption spectra (`A_SAMP*UVON.csv`), and corresponding reaction time file (`Parameters_Time.csv`) was processed as follows.

PL baseline correction:

$$I_{PL,corr}(\lambda) = I_{PL,raw}(\lambda) - I_{PL,dark}(\lambda) \quad \textbf{(Equation. S1)}$$

Absorbance calculation and baseline correction:

$A_{,\text{raw}}(\lambda) = -\log_{10} [(I_{\text{avg}}(\lambda) - I_{\text{dark}}(\lambda)) / (I_{\text{light}}(\lambda) - I_{\text{dark}}(\lambda))]$ **(Equation. S2)**

$A(\lambda) = A_{,\text{raw}}(\lambda) - \text{mean} [A_{,\text{raw}}(\lambda)]_{750–770}$ **(Equation. S3)**

Absorbance at specific wavelengths:

$A_{365} = A(\lambda_{i365}), \; i_{365} = \text{argmin} \mid \lambda - 365\mid$ **(Equation. S4)**

$A_{300} = A\ (\lambda_{i300}), \; i_{300} = \text{argmin} \mid \lambda - 300\mid$ **(Equation. S5)**

Peak emission wavelength and intensity:

$\lambda_{EP} = \lambda_j, \; j = \text{argmax } I(\lambda)$ **(Equation. S6)**

Integrated PL area:

$PL_{area} = \int I_{mean}(\lambda)\, d\lambda$ **(Equation. S7)**

Emission linewidth (FWHM) in nm and eV:

$FWHM_\lambda = \lambda_{k2} - \lambda_{k1}$ **(Equation. S8)**

$FWHM_E = E\ (\lambda_{k1}) - E\ (\lambda_{k2})$ **(Equation. S9)**

Time stamping and feature logging:

The reaction time was extracted from the corresponding time file. All extracted parameters, including λ_{em}, PL_I, FWHM, P_{PLQY}, A_{365}, and reaction time along with file identifiers (experiment and iteration numbers) were appended to a cumulative 'extracted_features.csv' file, with headers generated upon first write.

S3. P_{PLQY} validation

To enable autonomous, high-throughput optimization of MHP NCs' photoluminescence quantum yield (PLQY) during PIAER, we defined an *in situ*-measured proxy PLQY defined as the integrated PL emission area (background/dark corrected with an HDX spectrometer, Ocean Optics) normalized by the sample's absorbance at the excitation wavelength. We validated this metric separately for the two PIAER chemistries with a two-step calibration. First, cross-instrument transferability was established by comparing *in situ* proxy measurements with benchtop *ex situ* measurements (FS5, Edinburgh Instruments). **Figure S2A** (Br→Cl) and **Figure S2** (Br→I) present a linear correlation of the *in situ* P_{PLQY} *vs*. *ex situ* measurements

across the full signal range. Second, the P_{PLQY} proxy was mapped to absolute PLQY of the same samples measured with an integrating sphere (FS5 spectrometer). **Figure S2B** (Br→Cl) presents a linear correlation across the PLQY values ranging from 0%–70%, and **Figure S2D** (Br→I) confirms linear correlation up to ~PLQY= 100%. Each point is an average of four replicates and lines are unconstrained least-squares fits. During each in-flow PIAER experiment, P_{PLQY} values were converted to *ex situ*-equivalent values and then to absolute PLQY values using the linear correlations shown in **Figure S2**. Periodic checks showed no measurable drift, establishing the normalized PL-area proxy as an instrument-consistent, quantitatively reliable surrogate for the absolute PLQY across the operational ranges of both Br→Cl and Br→I PIAER, suitable for closed-loop control and model training.

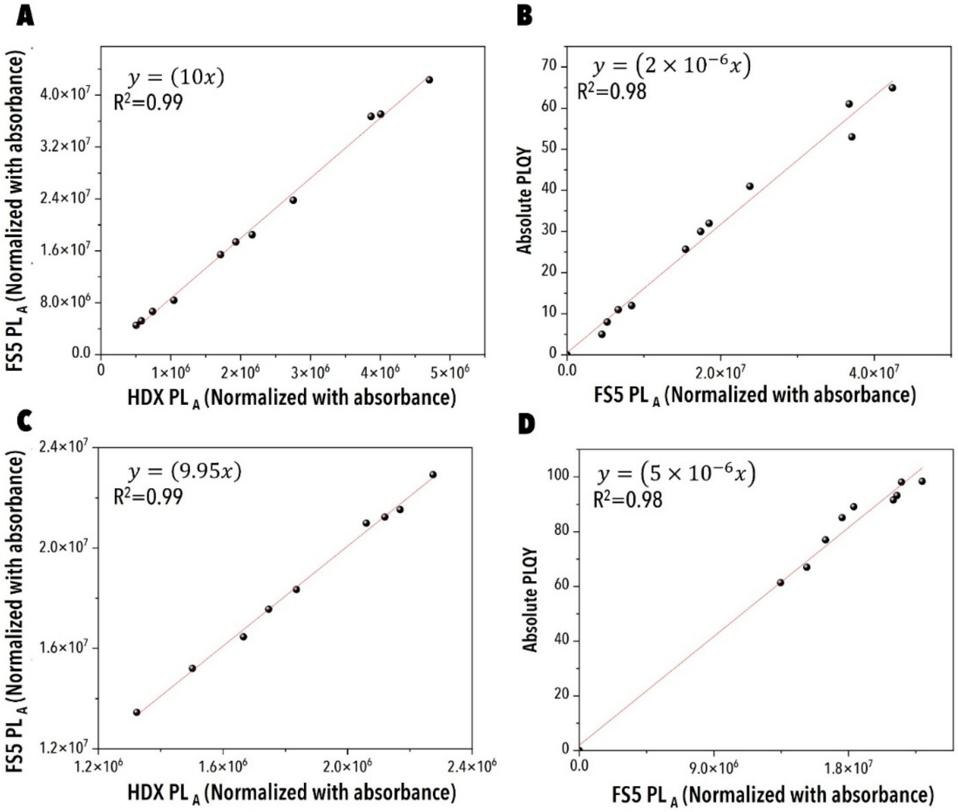

Figure S2. *Proxy PLQY calibration for two PIAER chemistries. (A–B) Br→Cl PIAER. (A) Cross-instrument calibration of the proxy signal. The in situ (HDX)- vs. ex situ (FS5)-measured normalized PL area by absorbance at the excitation wavelength for 11 different MHP NC samples. (B) The ex situ-measured proxy PLQY values (FS5) vs. absolute PLQY from an integrating sphere for 11 different MHP NC samples. (C–D) Br→I PIAER. (C) The in situ (HDX)- vs. ex situ (FS5)-measured normalized PL area by absorbance at the excitation wavelength for 9 different MHP NC samples. (D) The ex situ-measured proxy PLQY values (FS5) vs. absolute PLQY from an integrating sphere for 9 different MHP NC samples.*

S4. Photon Flux Measurements

Chemical actinometry within the developed microfluidic photoreactor was performed using *4,4'-dimethylazobenzene* (DMAB) as the actinometer. The incident photon flux, q_0, was determined from the absorbed photon flux by applying the Beer–Lambert law to the E–Z photoisomerization of DMAB in acetonitrile under 365 nm irradiation, following our previously reported methodology.[1,2] The photo-isomerization kinetics of DMAB are governed by the following relations

$$\frac{d[E]}{dt} = -\frac{d[Z]}{dt} = \frac{q_o(f_{avg})_{DMAB}}{V(\varepsilon_{E,365}[E] + \varepsilon_{Z,365}[Z])}(\Phi_{EZ}\varepsilon_{E,365}[E] - \Phi_{ZE}\varepsilon_{Z,365}[Z]) \quad \textbf{(Equation. S10)}$$

$$A = (\varepsilon_E[E] + \varepsilon_Z[Z])D \quad \textbf{(Equation. S11)}$$

$$(f_{avg})_{DMAB} = \frac{1}{2}\int_0^{\pi} 1 - 10^{-(\varepsilon_{E,365}[E] + \varepsilon_{Z,365}[Z]D sin\theta)}\, sin\theta\, d\theta \quad \textbf{(Equation. S12)}$$

where [*E*] and [*Z*] are the concentrations of the E- and Z-isomers of DMAB, *V* is the droplet volume, *D* is the microreactor diameter, $\varepsilon_{E,365}$ and $\varepsilon_{Z,365}$ are the molar extinction coefficients at 365 nm, and Φ_{EZ} and Φ_{ZE} are the quantum yields for *E–Z* and *Z–E* photoisomerization, respectively. The fraction of absorbed photons, $(f_{avg})_{DMAB}$, is calculated using Eq. S3.

Experimentally, the absorbance of a 0.7 mM DMAB droplet (10 μL) at 333 nm (A_{333}) was monitored *in situ* at different UV LED input currents. As shown in **Figure S3A**, the absorbance decay accelerates with increasing LED current, reflecting higher photon flux driving the E–Z isomerization. To establish a quantitative calibration, the LED output power was measured as a function of input current (**Figure S3B**), and the corresponding photon flux was determined by numerical integration of the isomerization kinetics and comparison with experimental absorbance profiles (**Figure S3C**). This approach provided a direct correlation between LED input current and incident photon flux across the operational range of the microfluidic photoreactor, enabling accurate determination of irradiation conditions for subsequent photochemical experiments.

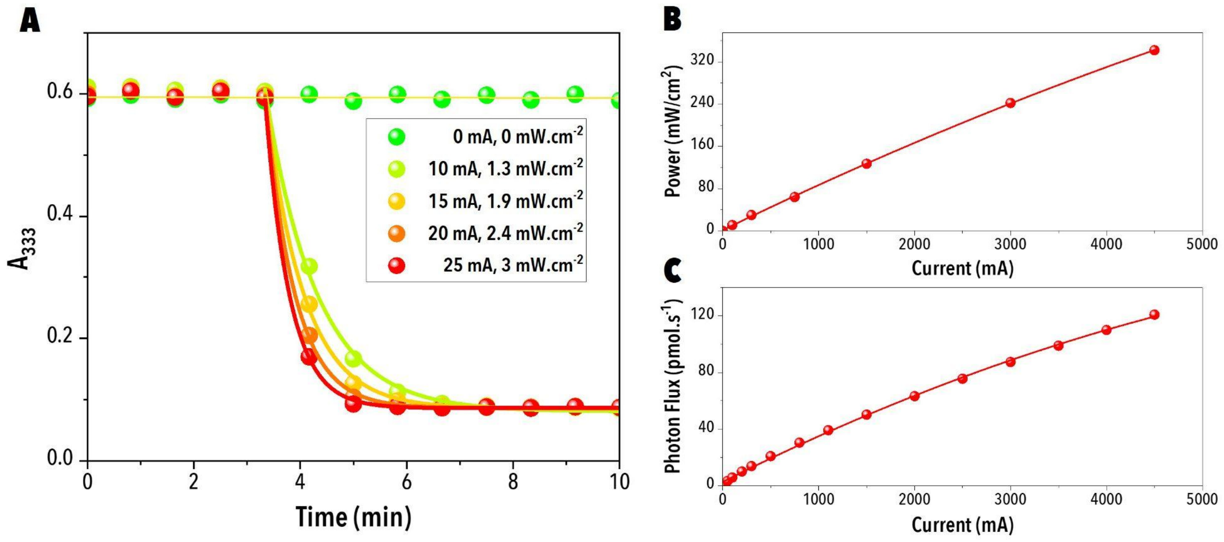

Figure S3. Chemical actinometry in the photo-flow microreactor. *(A)* *In situ monitoring of the absorbance at 333 nm for a 0.7 mM DMAB droplet (10 µL) under 365 nm irradiation at different UV LED input currents (0–25 mA). Faster absorbance decay is observed at higher LED currents due to increased photon flux. (B) Calibration of UV LED output power as a function of LED input current. (C) Incident photon flux determined from chemical actinometry as a function of LED input current, obtained by numerical integration of the isomerization kinetics and fitting to experimental absorbance decay. Experimental conditions: [DMAB] = 0.7 mM; droplet volume = 10 µL; T = 23 °C.*

S5. PIAER Visualization

Br → Cl Anion Exchange. To examine the role of NCs loading on the PIAER process, the data-driven digital twin was used to map emission peak wavelength, FWHM, and P_{PLQY} as functions of photon flux and reaction time. At lower NC concentrations (0.2), the emission peak shifts gradually to longer wavelengths with increasing photon flux and irradiation time. In this regime, FWHM decreases to a minimum at intermediate conditions, indicating improved spectral uniformity, while P_{PLQY} shows a transient maximum before decreasing at high flux or long reaction times due to defect formation.

At higher NC concentrations (0.5), the emission peak shifts occur more steeply, consistent with faster halide incorporation. However, the emission linewidth broadens and P_{PLQY} declines more rapidly under strong irradiation, showing that higher particle density accelerates exchange but also amplifies nonradiative losses. Together, these trends illustrate how photon flux, reaction time, and NC concentration jointly determine the balance between efficient bandgap tuning and preservation of optical quality.

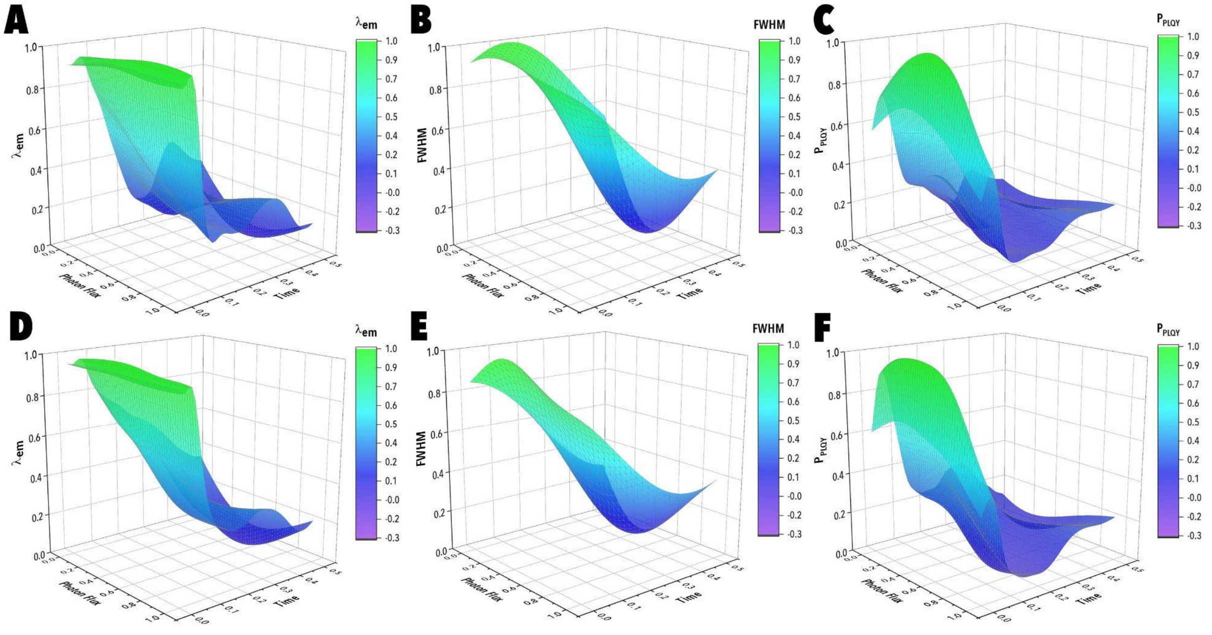

Figure S4. *Digital twin-derived surface plots for Br → Cl anion exchange. Predicted λ_{em}, FWHM, and normalized P_{PLQY} as functions of photon flux and reaction time at fixed normalized haloalkane of 0.5 concentrations. (A–C) Normalized [NC] = 0.2. (D–F) Normalized [NC] = 0.5.*

Br → I Anion Exchange. To investigate the effect of NC concentration on bromide-to-iodide anion exchange reaction, the data-driven digital twin was used to map the predicted emission peak wavelength, FWHM, and P_{PLQY} as functions of photon flux and reaction time. At a lower NC concentration (0.2), the emission peak shifts steadily to longer wavelengths with increasing photon flux and time, reflecting progressive iodide incorporation. In this regime, FWHM shows a broad maximum at intermediate conditions, indicating heterogeneous exchange pathways, while P_{PLQY} displays a non-monotonic trend with a rise followed by quenching under prolonged irradiation. At higher NC concentrations (0.5), the red-shift in emission is accelerated, consistent with faster exchange kinetics. However, this red-shit comes with broader FWHM features and a sharper P_{PLQY} drop at high flux and long reaction times, suggesting that higher particle density intensifies both iodide substitution and defect-related nonradiative losses. Together, these maps, shown in **Figure S6**, highlight the kinetic–optical trade-offs that emerge when tuning iodide incorporation in different loading regimes.

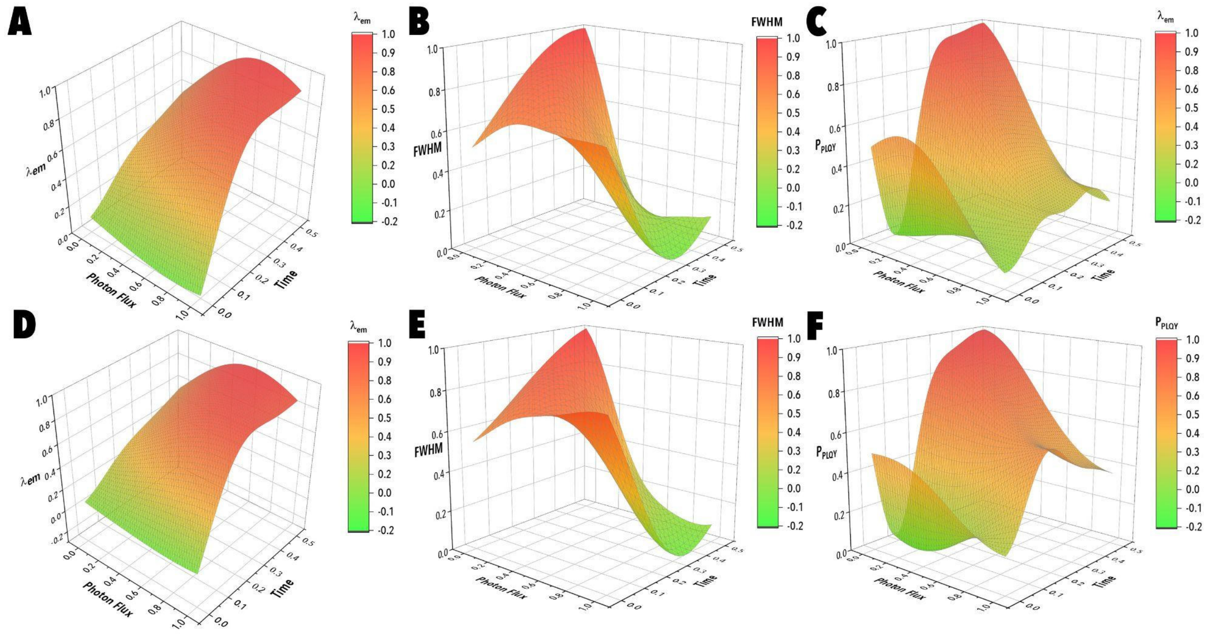

Figure S5. *Digital twin-derived surface plots for Br → I anion exchange. Predicted λ_{em}, FWHM, and normalized P_{PLQY} as functions of photon flux and reaction time at fixed normalized haloalkane of 0.5 concentrations. (A–C) Normalized [NC] = 0.2. (D–F) Normalized [NC] = 0.5.*

S6. Autonomous Decision-Making Algorithm

Decision Policy and Parameter Space. The autonomous decision-making algorithm of the developed fluidic self-driving laboratory (FSDL) operates as a constrained, multi-objective Bayesian optimization (BO). In each optimization iteration, the artificial intelligence (AI) agent proposes new synthesis conditions designed to drive the reaction toward a user-defined optical target. The input variables consist entirely of continuous parameters: (i) photon flux, (ii) haloalkane volume fraction, (iii) NCs concentration, and (iv) reaction time. The output variables are λ_{em}, P_{PLQY}, and emission linewidth (FWHM).

Prior to model training and prediction, all inputs and outputs are min–max normalized to the [0,1] interval using fixed lower and upper bounds established from instrument capabilities and chemically accessible regimes. This normalization step ensures consistent scaling across objectives and facilitates convergence in the multi-objective BO process.

Objective Formulation and Transformations. The optimization objective is to match the λ_{em} to a desired target value (EP_{tgt}) while simultaneously maximizing P_{PLQY} and minimizing FWHM. To achieve this goal

within a hypervolume-based optimization framework, the model outputs are transformed into three scalar objectives, which are then stacked into a single objective tensor:

Objective 1: Proximity to Target EP

$Obj_1 = \max \{ \text{Res} - | \text{EP}_{\text{pred}} - \text{EP}_{\text{tgt}} | , 0\}$ **(Equation. S13)**

Here, EP_pred is the surrogate model's predicted emission peak in nanometers, Res = 0.02 × EP_{tgt} defines the 2% permissible deviation from the target.

Objective 2: Maximize P_{PLQY}

The predicted P_{PLQY} is directly maximized as a normalized MHP NC quality metric.

$Obj_2 = P_{\text{PLQY}}$ **(Equation. S14)**

Objective 3: Minimize Broadening

Lower predicted FWHM values result in higher scores, encouraging narrower emission spectra.

$Obj_3 = 1 - \text{FWHM}$ **(Equation. S15)**

All transformed objectives are normalized to the [0,1] interval before hypervolume computation.

Surrogate Model Architecture. The surrogate model consists of independent Gaussian Process regression (GPR) models for λ_{em}, P_{PLQY}, and FWHM, aggregated into a ModelListGP. Each GPR employs Matérn 2.5 kernel with outcome standardization to account for scale differences between objectives. Hyperparameters (length scales, signal variance, and noise level) are optimized *via* maximization of the log marginal likelihood using BoTorch's fit gpytorch model, which balances model flexibility and generalization.

Acquisition Function and Candidate Selection. The AI agent employs noisy expected hypervolume improvement (qNEHVI) as the acquisition function, a one-step Bayes-optimal policy that maximizes the expected gain in Pareto-dominated hypervolume under noisy measurements. The reference point for hypervolume calculation is inferred dynamically from the current objective distribution, using slightly pessimistic per-objective quantiles to ensure strictly positive hypervolume contributions from all evaluated points.

Candidate generation proceeds as follows: (i) the acquisition function is evaluated over Sobol-sampled starting points in the normalized input space, (ii) each starting point is locally optimized *via* multistart

gradient-based optimization (10 restarts, 256 raw samples), (iii) the top-scoring candidate is selected and inverse-transformed to physical units using the fitted MinMaxScaler, and (iv) the candidate is appended to the active design space for execution in the next experimental iteration. A hard feasibility constraint (FWHM≤0.12eV) is imposed during both model training and candidate filtering to exclude spectrally poor conditions from consideration.

To quantitatively track optimization progress, we computed the hypervolume (HV) after each experiment using BoTorch's implementation of the hypervolume indicator. HV was evaluated in both two- and three-dimensional objective spaces. In the two-dimensional case, only experiments with emission peaks within ±4 nm of the target λ_{em} were considered, with objectives of maximizing normalized P_{PLQY} and minimizing FWHM (expressed as 1−FWHM). In the three-dimensional case, the objectives included (i) λ_{em}, (ii) P_{PLQY}, and (iii) 1−FWHM, directly mirroring the BO objective formulation. Reference points of [0,0][0,0][0,0] (2D) and [0,0,0][0,0,0][0,0,0] (3D) were chosen to ensure strictly positive HV contributions. The resulting cumulative HV and incremental HV improvements provided a sensitive and quantitative metric for assessing the rate at which the Pareto-front expanded throughout the optimization campaign.

Closed-Loop Orchestration via SSH. The optimization loop is executed in a distributed client–server configuration, linking the lab hardware controller ("PIAER PC") with the optimization server over a secure SSH protocol. The sequence is as follows: (i) *Data Ingress*: After each PIAER experiment, the PIAER PC generates two files: condition_table_server.csv (recording the input conditions) and extracted_features.csv. These files are transferred to the optimization server via SSH. (ii) *Model Update and Optimization:* The server loads the incoming data, applies the predefined normalization, updates the GPR surrogate models, computes the qNEHVI acquisition function, and proposes the next experimental condition in normalized form. (iii) *Inverse Transformation and Condition Table Update:* The proposed candidate is inverse-scaled to physical units and appended to Condition Table.csv on the server. (iv) *Data Egress:* The updated Condition Table.csv is transferred back to the PIAER PC via SSH. (v) *Status Signaling:* A signal.txt file is uploaded to the PIAER PC to indicate task completion ("Pass") or failure ("fail"), allowing the hardware controller to either proceed with the next run or pause for intervention. All file transfers are atomic and overwrite-safe to avoid race conditions, ensuring synchronization between server and laboratory hardware.

Initialization, Constraints, and Stopping Criteria. The optimization begins with an initial design generated *via* Sobol sampling within the predefined bounds for each input parameter. Physical and hardware safety constraints (*e.g.*, photon-flux limits, volumetric flow limits, and solvent compatibility) are enforced as hard bounds in the input space. Soft constraints, such as the spectral-quality gate on FWHM, are implemented through candidate filtering and objective shaping. The optimization continues until the allocated

experimental budget is exhausted or the incremental hypervolume improvement falls below a pre-set threshold, indicating convergence toward a locally optimal Pareto-front.

Computational Environment. All optimization routines are implemented in PyTorch and BoTorch using double-precision arithmetic. GPU acceleration is utilized when available, with an automatic fallback to CPU execution on unsupported hardware. Environment isolation is enforced *via* non-interactive Conda execution to ensure reproducibility across optimization runs. Normalization, inverse scaling, and file I/O operations are implemented using scikit-learn and pandas, with all SSH transfers handled *via* a custom Python wrapper for robust authentication and error handling.

S7. Continuous Microfluidic Photoreactor

We transferred single droplet-optimized recipes to a segmented-flow PIAER without retuning the surrogate model or additional BO iterations by holding constant the three parameters that control anion exchange kinetics at fixed NC loading: (i) NCs and halide volume fractions (DCM for Br→Cl, 2-iodopropane for Br→I), (ii) photon flux, and (iii) reaction time, $\tau = V/Q_{\text{total}}$, where V is the reactor volume, and Q_{total} is the total volumetric flowrate in a 250 μL UV-irradiated spiral photoreactor. Prediluted $CsPbBr_3$ NCs in toluene and the halide solvent merge at a T-junction, segmented with a nitrogen (N_2) stream before entering the microfluidic photoreactor (**Figure S6**). Each continuous flow PIAER was operated as shown in **Table S1**, after which the effluent was collected and its absolute PLQY measured**.** DMF rinses between conditions restore the fluidic lines to the solvent baseline and eliminate carryover. Since both the photonic dose (irradiance × τ) and the halide availability (set by the halide-solvent fraction) are conserved relative to the single-droplet platform, the target emissions are reproduced in steady-state flow without additional tuning. The continuous microfluidic photoreactor's MHP NC manufacturing throughput is 220–250 mL.day^{-1} at τ=1.0 min, ~45–52 mL.day^{-1} at τ=4.8 min. **Figure S7** present the stability of the MHP NCs produced through continuous flow PIAER *via* the manufacturing mode of the SDFL.

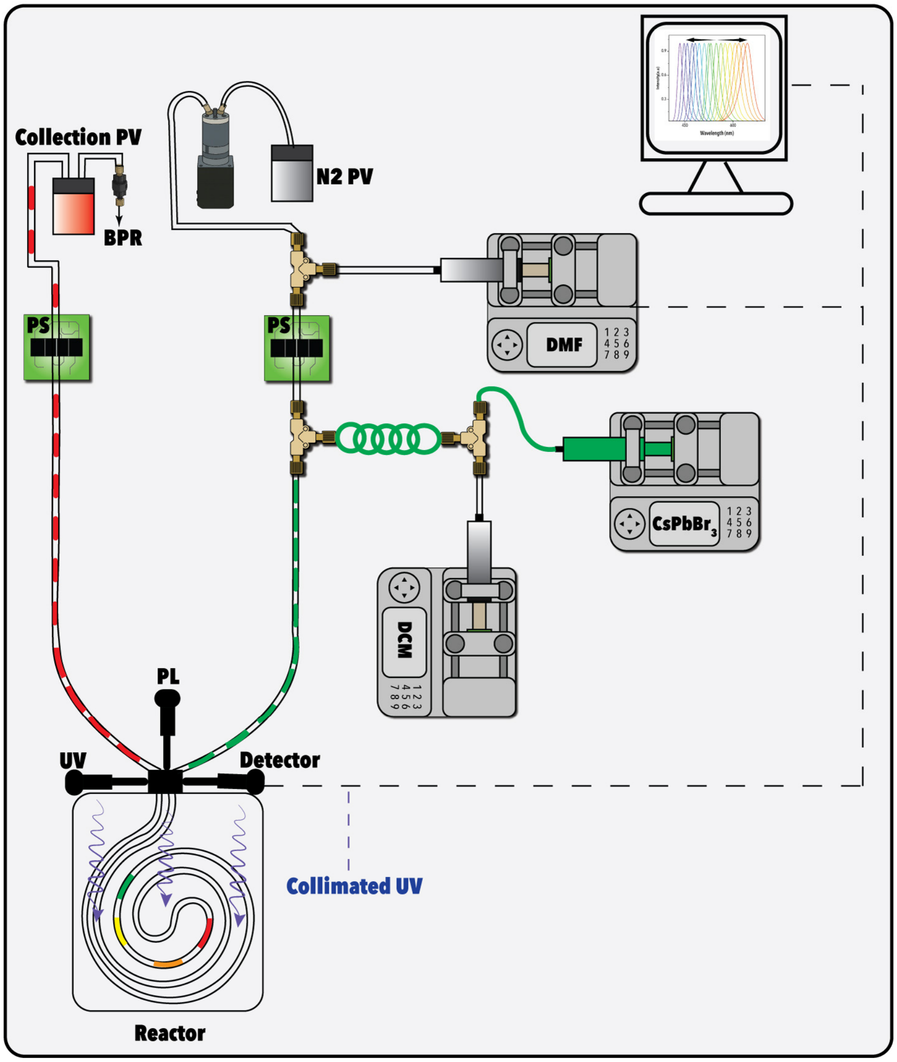

Figure S6. *Schematic of the continuous microfluidic photoreactor for knowledge transfer studies of the PIAER SDFL. Prediluted $CsPbBr_3$ NCs in toluene are delivered via syringe pump and merged at a T-junction with haloalkane (DCM)/ (Iodopropane) and DMF streams for cleaning between each continuous flow run, followed by mixing in a coiled braided mixer. The reaction stream is segmented by N_2 gas before entering the spiral photoreactor, where collimated UV irradiation induces anion exchange reaction. UV-Vis-NIR absorption and PL spectra are collected in situ during flow through the reactor. The product stream is directed to a back-pressure regulator (BPR) and collected in a pressurized vessel containing a 30 mL vial. All flow operations, reagent dosing, UV control, and spectral acquisitions are automated through a LabVIEW interface based on predefined input conditions.*

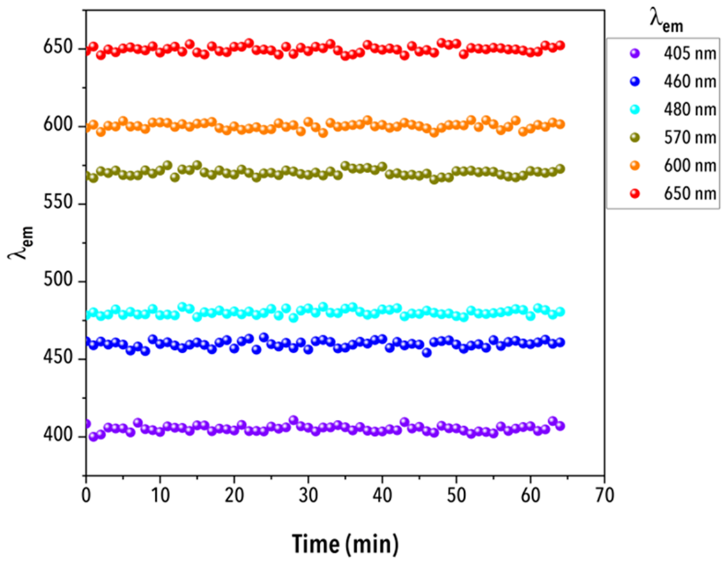

Figure S7. *Stability of peak emission wavelengths (λ_{em}) at 405, 460, 480, 570, 600, and 650 nm over 60 min of continuous operation. All target emissions remain constant within ±2 nm, confirming reproducibility of the process.*

Table S1. *Continuous-flow PIAER operating conditions for targeted emission wavelengths (250 µL photoreactor).*

Condition	Target λ_{em} (nm)	Photon (mA)	NCs (vol. frac)	Halide solvent	Halide vol. frac	Time (min)
1	405	1000	0.225	DCM	0.230	1.0
2	480	787	0.265	DCM	0.370	2.0
3	460	500	0.205	DCM	0.390	3.5
8	570	512	0.120	2-iodopropane	0.500	2.4
5	600	800	0.110	2-iodopropane	0.460	4.4
7	650	1420	0.300	2-iodopropane	0.600	4.8

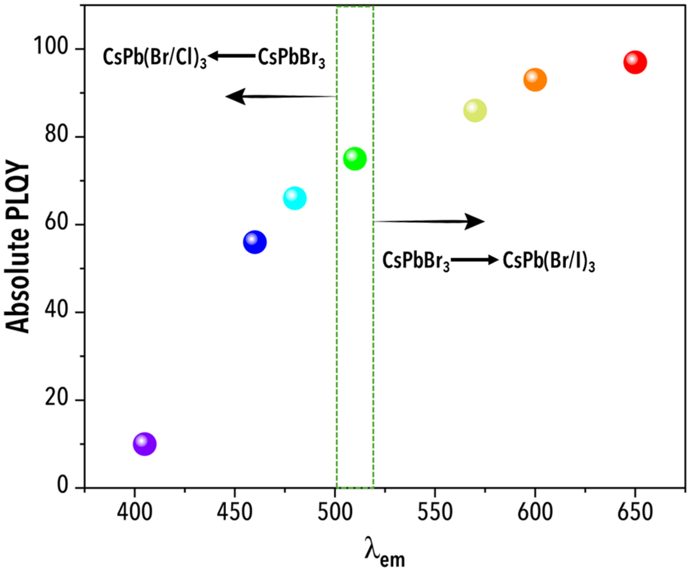

Figure S8. *Absolute PLQY of continuous-flow PIAER products at targeted emission wavelengths (measured immediately after collection)*.

S8. EDS Mapping

Table S2. *EDS analysis of $CsPb(Br/Cl)_3$ NCs with λ_{em} = 510 nm*

Z	Element	Family	Net intensity	Net backgrou nd	K-factor	Absorptio n correction	Atomic fraction	Mass fractio n	Fit error
-	-	-	counts	counts	-	-	%	%	%
35	Br	K	2.19E+03	9.35E+02	1.00E +00	1.00E+00	5.65E+0 1	3.76E+ 01	5.32E -01
55	Cs	L	2.21E+03	3.09E+03	5.79E- 01	1.00E+00	2.06E+0 1	2.29E+ 01	4.73E -01
82	Pb	L	2.18E+03	1.85E+03	9.84E- 01	1.00E+00	2.29E+0 1	3.96E+ 01	5.69E -01

Table S3. *EDS analysis of CsPb(Br/Cl)*$_3$ *NCs with* λ_{em} *= 480 nm*

Z	Element	Family	Net intensity	Net background	K-factor	Absorption correction	Atomic fraction	Mass fraction	Fit error
-	-	-	counts	counts	-	-	%	%	%
17	Cl	K	2.40E+02	1.57E+03	1.00E+00	1.00E+00	6.48E+00	1.97E+00	4.11E+00
35	Br	K	1.55E+03	8.72E+02	2.70E+00	1.00E+00	5.01E+01	3.43E+01	7.50E-01
55	Cs	L	1.79E+03	2.73E+03	1.56E+00	1.00E+00	2.10E+01	2.39E+01	6.03E-01
82	Pb	L	1.69E+03	1.85E+03	2.66E+00	1.00E+00	2.24E+01	3.97E+01	7.63E-01

Table S4. *EDS analysis of CsPb(Br/Cl)*$_3$ *NCs with* λ_{em} *= 460 nm*

Z	Element	Family	Net intensity	Net background	K-factor	Absorption correction	Atomic fraction	Mass fraction	Fit error
-	-	-	counts	counts	-	-	%	%	%
17	Cl	K	6.59E+02	1.28E+03	1.00E+00	1.00E+00	2.90E+01	9.36E+00	1.66E+00
35	Br	K	4.83E+02	5.79E+02	2.70E+00	1.00E+00	2.55E+01	1.85E+01	2.01E+00
55	Cs	L	1.05E+03	2.08E+03	1.56E+00	1.00E+00	2.00E+01	2.42E+01	1.04E+00
82	Pb	L	1.18E+03	1.33E+03	2.66E+00	1.00E+00	2.54E+01	4.79E+01	8.47E-01

Table S5. *EDS analysis of $CsPb(Br/Cl)_3$ NCs with λ_{em} = 405 nm*

Z	Element	Family	Net intensity	Net background	K-factor	Absorption correction	Atomic fraction	Mass fraction	Fit error
-	-	-	counts	counts	-	-	%	%	%
17	Cl	K	1.10E+03	6.57E+02	1.00E+00	1.00E+00	5.55E+01	2.19E+01	1.03E+00
35	Br	K	1.10E+02	3.42E+02	2.70E+00	1.00E+00	6.65E+00	5.91E+00	6.86E+00
55	Cs	L	8.30E+02	1.02E+03	1.56E+00	1.00E+00	1.81E+01	2.68E+01	9.92E-01
82	Pb	L	7.98E+02	6.72E+02	2.66E+00	1.00E+00	1.97E+01	4.54E+01	1.29E+00

Table S6. *EDS analysis of $CsPb(Br/I)_3$ NCs with λ_{em} = 570 nm*

Z	Element	Family	Net intensity	Net background	K-factor	Absorption correction	Atomic fraction	Mass fraction	Fit error
-	-	-	counts	counts	-	-	%	%	%
35	Br	K	1.05E+02	3.16E+02	1.00E+00	1.00E+00	3.97E+01	2.48E+01	6.22E+00
53	I	L	1.70E+02	1.55E+03	5.69E-01	1.00E+00	2.40E+01	2.39E+01	5.05E+00
55	Cs	L	9.24E+01	1.41E+03	5.79E-01	1.00E+00	1.27E+01	1.32E+01	9.18E+00
82	Pb	L	1.52E+02	5.69E+02	9.84E-01	1.00E+00	2.35E+01	3.81E+01	5.02E+00

Table S7. *EDS analysis of $CsPb(Br/I)_3$ NCs with λ_{em} = 600 nm*

Z	Element	Family	Net intensity	Net background	K-factor	Absorption correction	Atomic fraction	Mass fraction	Fit error
-	-	-	counts	counts	-	-	%	%	%
35	Br	K	1.59E+03	1.71E+03	1.00E+00	1.00E+00	1.79E+01	1.05E+01	6.00E-01
53	I	L	1.06E+04	9.54E+03	5.69E-01	1.00E+00	4.42E+01	4.12E+01	1.69E-01
55	Cs	L	4.31E+03	9.21E+03	5.79E-01	1.00E+00	1.75E+01	1.72E+01	3.71E-01
82	Pb	L	4.44E+03	3.55E+03	9.84E-01	1.00E+00	2.04E+01	3.11E+01	3.30E-01

Table S8. *EDS analysis of $CsPb(Br/I)_3$ NCs with λ_{em} = 650 nm*

Z	Element	Family	Net intensity	Net background	K-factor	Absorption correction	Atomic fraction	Mass fraction	Fit error
-	-	-	counts	counts	-	-	%	%	%
35	Br	K	2.85E+01	2.18E+02	1.00E+00	1.00E+00	8.86E+00	5.04E+00	2.04E+01
53	I	L	5.28E+02	1.23E+03	5.69E-01	1.00E+00	6.10E+01	5.52E+01	1.82E+00
55	Cs	L	7.80E+01	1.14E+03	5.79E-01	1.00E+00	8.77E+00	8.30E+00	1.05E+01
82	Pb	L	1.68E+02	3.87E+02	9.84E-01	1.00E+00	2.13E+01	3.15E+01	4.75E+00

S9. TEM and XRD Analysis

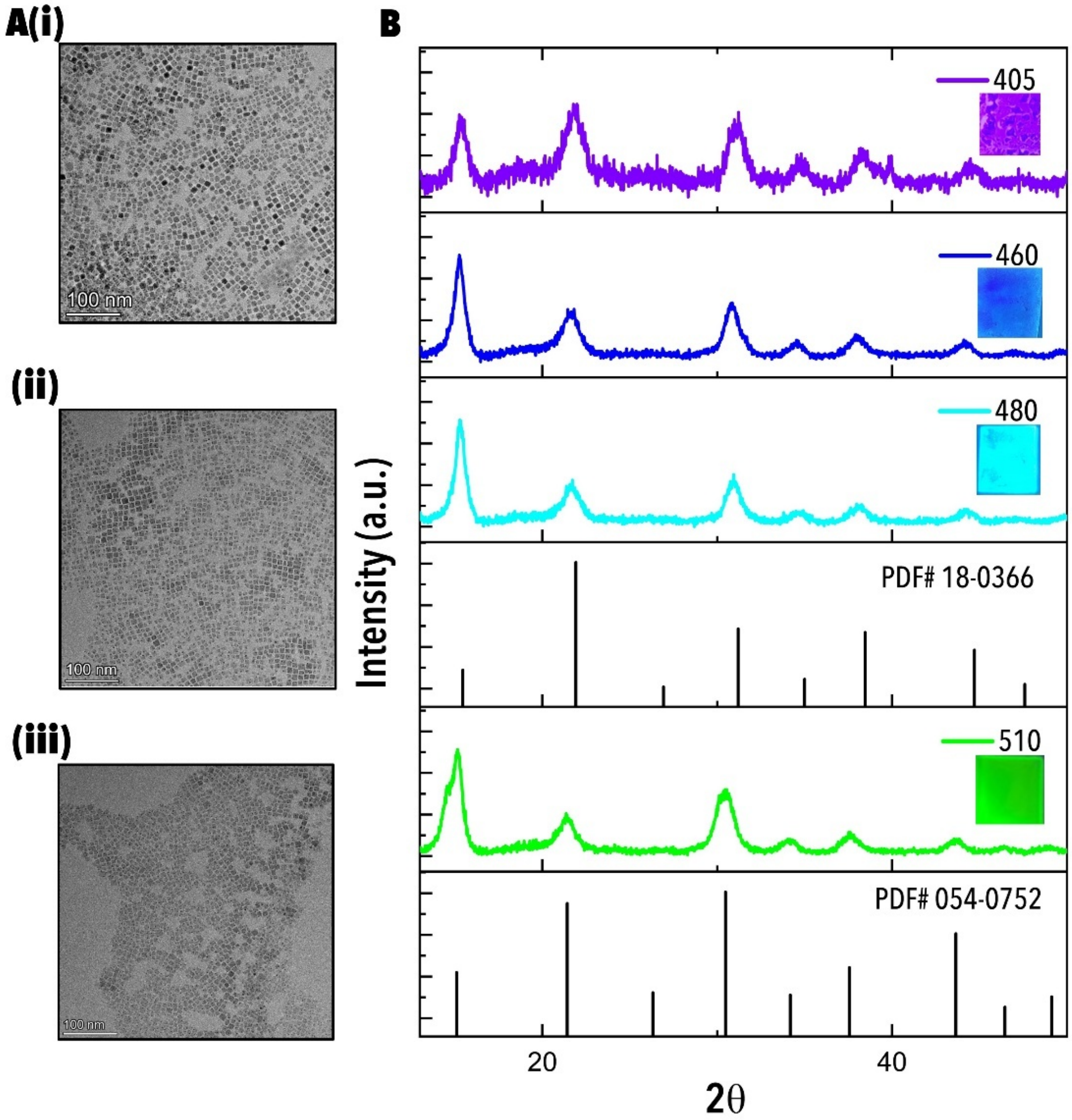

Figure S9. *Structural characterization of CsPb(Br/Cl)*$_3$ *NCs. (A) Representative TEM images of continuously manufactured MHP NCs via PIEAR FSDL at different targeted peak emission wavelengths: (i)* λ_{em} *= 405 nm, (ii)* λ_{em} *= 460 nm, and (iii)* λ_{em} *= 480 nm, showing uniform cubic morphologies. (B) XRD patterns of MHP NCs emitting at 405 nm, 460 nm, 480 nm, and 510 nm, compared with standard reference patterns (PDF# 18-0366 and PDF# 054-0752), confirming the retention of the perovskite crystal phase during halide exchange.*

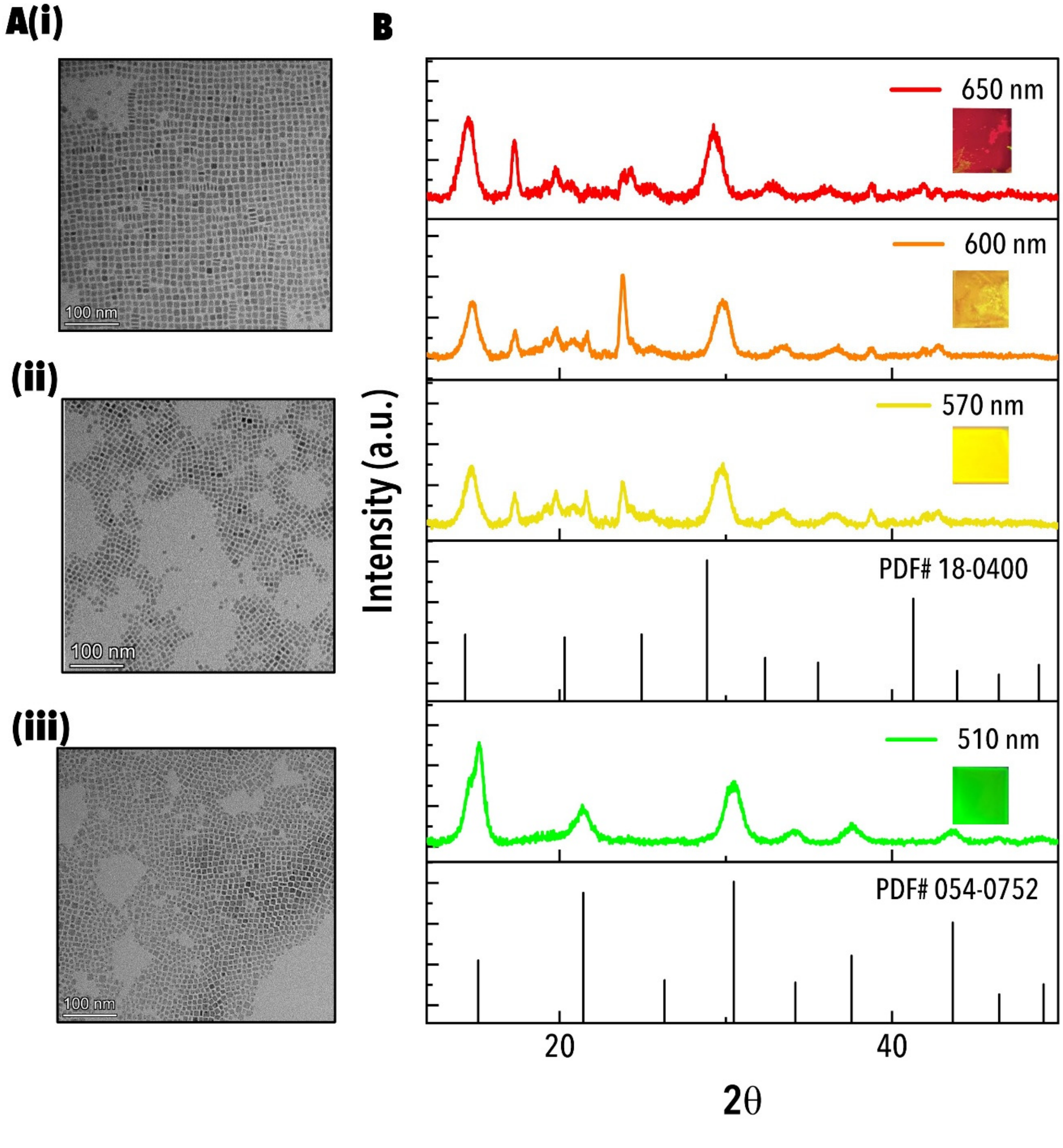

Figure S10. Structural characterization of $CsPb(Br/I)_3$ NCs. *(A) Representative TEM images of continuously manufactured MHP NCs via PIEAR FSDL at different targeted peak emission wavelengths: (i) λ_{em} =650 nm, (ii) λ_{em} =600 nm, and (iii) λ_{em} =570 nm, showing uniform cubic morphology. (B) XRD patterns of MHP NCs emitting at 510 nm, 570 nm, 600 nm, and 650 nm, compared with standard reference patterns (PDF# 054-0752 and PDF# 18-0400). The diffraction peaks confirm the perovskite phase and reveal systematic shifts consistent with progressive halide exchange from bromide to iodide.*

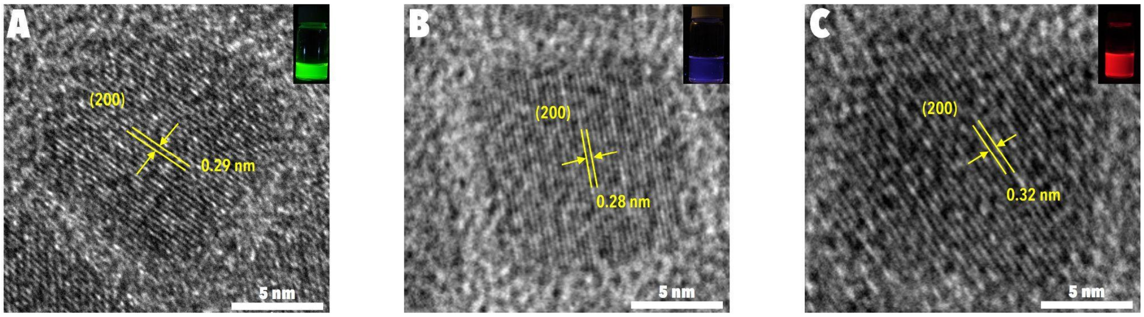

Figure S11. *High-resolution transmission electron microscopy (HRTEM) images of MHP NCs showing the (200) lattice planes. (A) Starting $CsPbBr_3$ NCs with a lattice spacing of 0.29 nm (λ_{em} = 510 nm). (B) Br → Cl exchange reduces the spacing to 0.28 nm (λ_{em} = 405 nm). (C) Br → I exchange expands the spacing to 0.32 nm (λ_{em} = 650 nm), consistent with incorporation of larger iodide ions and the corresponding red-shifted emission.*

S10. Starting $CsPbBr_3$ NCs

The starting $CsPbBr_3$ NC were synthesized using the hot-injection method described in the *Methods* section, producing colloidal quantum dots with well-defined optoelectronic properties. As shown in **Figure S12**, the UV-Vis absorption spectra displays a distinct excitonic feature in the visible region, while the corresponding PL emission is centered at ~510 nm with FWHM~20 nm, indicative of uniform NC size distribution and high crystalline quality.

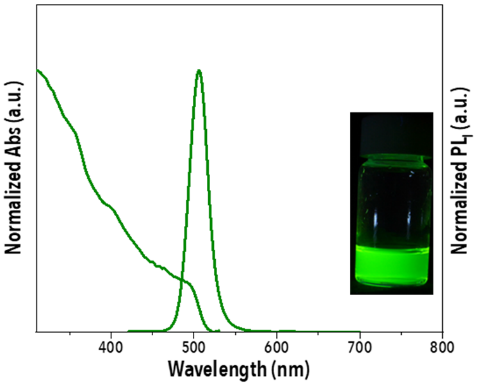

Figure S12. *Normalized UV-Vis absorption and PL spectra of as-synthesized $CsPbBr_3$ NCs prepared via hot-injection, recorded in situ in the microfluidic photoreactor. Inset: photograph of the colloidal suspension under UV illumination, showing the bright green emission.*

S11. LabVIEW Interface for Closed-loop PIAER

The custom-developed LabVIEW automation interface (**Figure S14-15**) integrates experimental setup, real-time spectral monitoring, and automated execution for the PIAER SDFL. The process control panel (**Figure S14A**) allows the user to enable or disable workflow steps such as fluidic line washing and priming with DCM/halide, manual UV LED control, and emergency stop functions. PIAER process status indicators (**Figure S14B**) provide real-time feedback on droplet detection, directional movement, spectrometer threshold triggers, UV LED activation, spectra acquisition, and experiment completion. Sample and iteration metadata panels (**Figure S14C–D**) track experimental identity and current progress, while the UV LED activation control (**Figure S14E**) defines the iteration at which illumination begins. The oscillation completion indicator (**Figure S14F**) signals SDFL's readiness for the next sequence step, and the droplet detection sensitivity setting (**Figure S14G**) enables adjustment of the spectrometer threshold to ensure reliable droplet confirmation and the trigger oscillation. Elapsed experiment time (**Figure S14H**) is displayed for monitoring progress, and wash/oscillation settings (**Figure S14I**) control line rinsing frequency and droplet mixing rates. The current condition display (**Figure S14J**) summarizes the active experimental parameters, and the UV–Vis absorption panel (**Figure S14K**) visualizes real-time optical signals from droplet monitoring, which are used to trigger droplet oscillation. File path selectors at the bottom of the control panel assign storage locations for all raw spectra, condition tables, and experimental metadata. Once configured, the SDFL autonomously executes droplet formation, oscillation, UV LED irradiation, optical data acquisition, and programmed washing/refill cycles with minimal operator intervention.

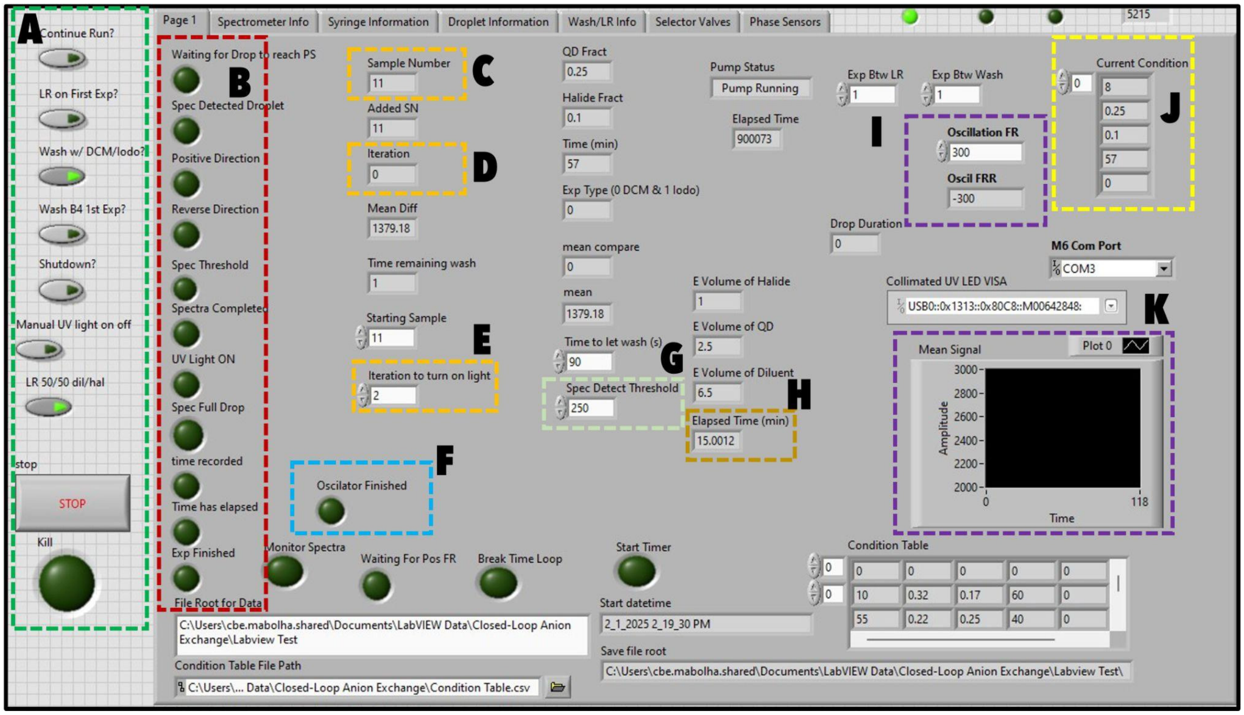

Figure S13. LabVIEW interface for SDFL's hardware automation. *Annotated PIAER process control interface with different functions: (A) global run controls for workflow toggling and emergency stop; (B) process status indicators for the droplet detection, UV LED, and experiment completion; (C–D) sample and iteration metadata; (E) UV LED activation settings; (F) oscillation completion indicator; (G) droplet detection threshold adjustment; (H) elapsed experiment time; (I) wash and oscillation rate controls; (J) active condition display; and (K) live optical signal plotting for droplet oscillation triggering and monitoring. File paths at the bottom define storage locations for spectral data, condition tables, and logs. Once parameters are set, the system autonomously executes droplet formation, oscillation, UV LED irradiation, real-time spectral acquisition, and programmed washing/refill cycles.*

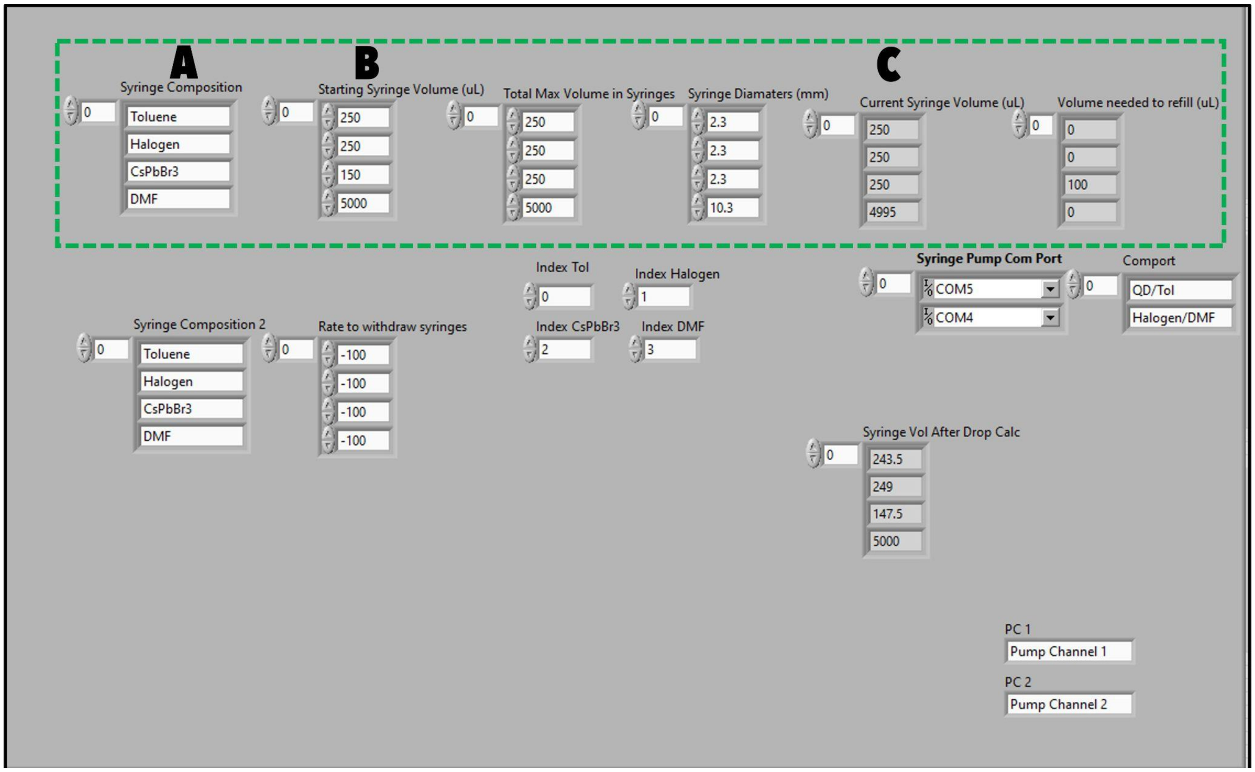

Figure S14. LabVIEW fluid delivery control interface. *Interface for configuring and monitoring syringe-based precursor delivery. (A) Precursor composition loaded in syringe 1, defining solvent, halide, $CsPbBr_3$ NC dispersion, and DMF assignments. (B) Initial syringe parameters of precursor 1 including starting volume, maximum capacity, and syringe diameter. (C) Real-time precursor delivery monitoring, showing current volume and calculated refill requirement, with automatic refill triggered below threshold. Additional controls set withdrawal rates, syringe index assignments, and pump COM port connections for NCs/toluene and halide/DMF delivery.*

S12. Reproducibility and Cross-Scale Consistency of $CsPbX_3$ Nanocrystals

Table S9. *Percent Error between Single-Droplet and Continuous-Flow $CsPbX_3$ Nanocrystals*

Target λ_{em} (nm)	$\Delta\lambda_{em}$ (%)	ΔFWHM (%)	ΔP_{PLQY} (%)
405	0.25	5.0	3.8
460	0.22	4.5	2.4
480	0.21	4.2	3.5
570	0.18	3.7	3.4
600	0.17	3.6	4.4
650	0.15	3.3	4.3

All deviations are expressed as % error between continuous-flow and single-droplet measurements. Δ% = 100 × (Continuous Flow – single Droplet)/single Droplet for each metric. All values confirm <5% deviation, demonstrating reproducibility and direct transferability of optical properties across scales.

Table S10. *Coefficient of Variation (CV%) in Continuous-Flow for 30 minutes*

Target λ_{em} (nm)	CV (λ_{em}) (%)	CV (FWHM) (%)	CV (PLQY) (%)
405	0.22	2.8	4.8
460	0.19	2.6	4.6
480	0.18	2.5	4.7
570	0.16	2.3	5.1
600	0.15	2.4	5.4
650	0.14	2.2	5.6

The coefficient of variation (CV%) was calculated as (σ / μ) × 100 across four independent continuous-flow replicates. All λ_{em} and PLQY CV values were below 0.3% and 6%, respectively, indicating excellent optical stability and reproducibility under steady-state operation of the fluidic self-driving laboratory platform.

S13. In silico digital-twin convergence of Bayesian optimization.

Hypervolume progression as a function of experiment number for emission targets at λ_{em} = 405 nm (purple) and 570 nm (orange) conducted *in silico* using a ground-truth GPR model (i.e., data-driven digital twin) trained on the in house generated experimental data. Each curve represents the mean of three independent BO runs initialized with five Latin Hypercube Sampling (LHS) points, with shaded regions indicating the corresponding standard deviations. The vertical dashed line separates the initialization (LHS) phase from the Bayesian optimization (BO) phase. Each campaign was iterated for 35 cycles using the same digital-twin-based model and *q*LogNEHVI acquisition function. The rapid early increase followed by a plateau in hypervolume reflects efficient exploration and convergence toward the Pareto-optimal front, validating the robustness and consistency of the digital-twin-guided optimization framework across multiple emission regimes.

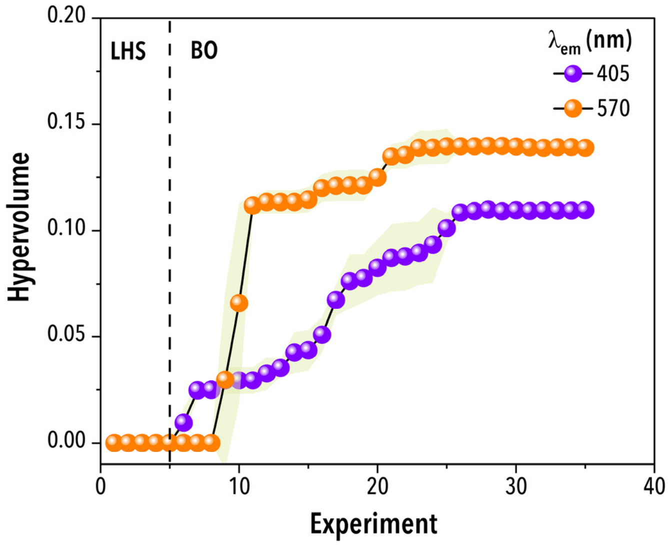

Figure S15. *BO convergence study conducted in silico using the data-driven digital twin trained on the in house generated experimental PIAER data for two target peak emission wavelengths, 405 nm and 570 nm."*

S14. Kinetics and Control Experiment

To quantify the light-driven kinetics of the photo-induced anion exchange reactions, intrinsic rate constants ($|k|$) were extracted from the UV-on segments of the spectral trajectories and correlated with the actinometry-derived photon flux. The resulting linear dependence (**Figure S16A**) confirms that both Br→Cl and Br→I exchanges proceed under photon-limited conditions. The steeper slope for Br→Cl indicates faster intrinsic kinetics and a lower activation barrier relative to Br→I substitution.[3] Control experiments performed under identical flow and chemical conditions but without UV illumination (**Figure S16B–C**) show no measurable shift in emission or absorbance over the entire residence time, verifying that the exchange is inactive in the dark and proceeds exclusively under irradiation.

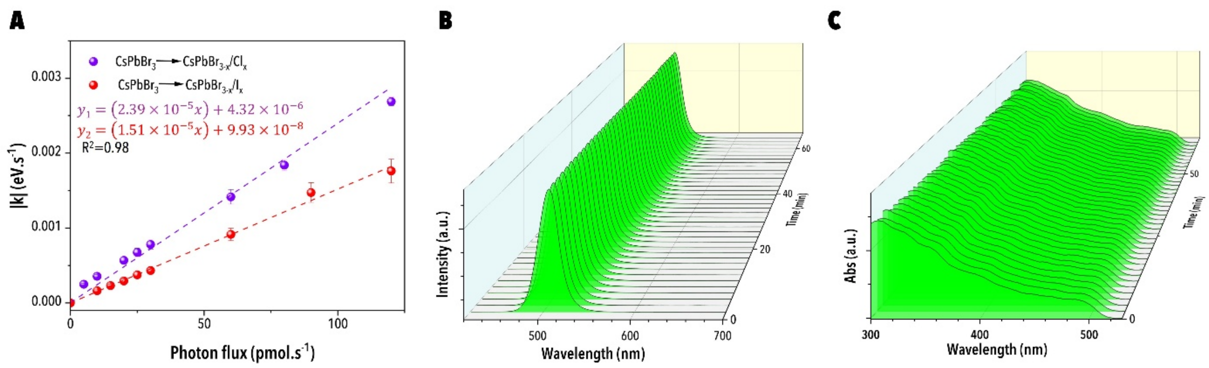

Figure S16. *Intrinsic kinetics and dark-control validation for photo-induced anion exchange reactions. (A) Dependence of the intrinsic kinetic rate constant (|k|, eV·s⁻¹) on the applied photon flux (pmol·s⁻¹) for the Br→Cl (purple) and Br→I (red) exchanges in $CsPbBr_3$ nanocrystals. Both reactions display a linear flux dependence, confirming a photon-limited regime, with the Br→Cl exchange proceeding faster due to its lower activation barrier and enhanced halide mobility. (B, C) Time-resolved photoluminescence (PL) and absorbance (Abs) spectra for a dark-control experiment* **(**[NC] = 4.2 µM**,** [1-iodopropane] = 0.13 M**,** UV LED off**,** residence time = 65 min**,** T = 23 °C**).** *No measurable spectral evolution is observed over time, confirming that halide exchange is inactive in the absence of illumination. Together, these data demonstrate that the reaction is strictly light-activated***,** *thermally stable, and governed by photon-flux-dependent intrinsic kinetics***.**

S15. TEM Size Distribution

Size-distribution analysis of $CsPbX_3$ nanocrystals before and after photo-induced anion exchange.

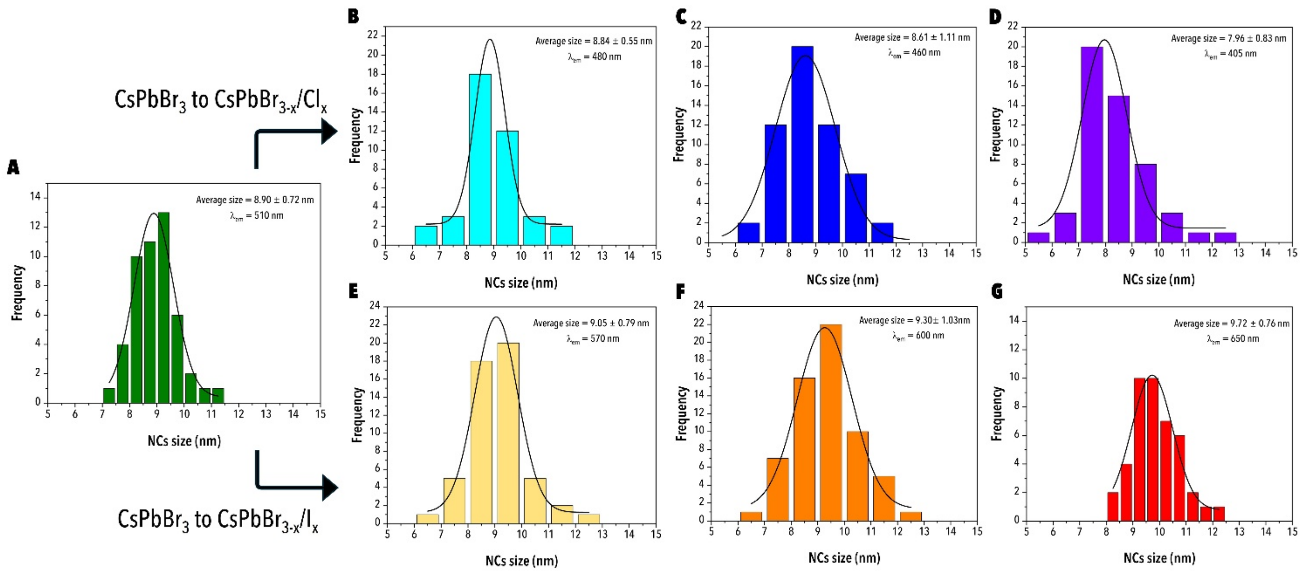

Figure S17. *(A–D) Br→Cl exchange series: representative size histograms for* $CsPbBr_3$ *(*λ_{em} *= 510 nm),* $CsPb(Br/Cl)_3$ *(*λ_{em} *= 480 nm, 460 nm, and 405 nm) NCs. (E–G) Br→I exchange series: representative size histograms for* $CsPb(Br/I)_3$ *(*λ_{em} *= 570 nm, 600 nm, and 650 nm) NCs.*

S16. Yield and Energy Consumption

Summary of experimentally validated continuous-flow operating conditions corresponding to scaled-up autonomous PIAER campaigns at different emission targets. The table lists the steady-state residence time (τ), daily volumetric throughput, isolated nanocrystal yield, haloalkane composition (volumetric fraction), normalized reagent consumption (combined haloalkane + solvent use per milligram of isolated product), and energy demand per gram of nanocrystals. These values were derived from the photon-flux-calibrated microreactor data (**Figure S3B**) and the material balance analysis presented in **Section S7**.

Table S11. *Throughput and Resource Metrics of the Continuous-Flow Photoreactor*

Target λ_{em} (nm)	τ (min)	Throughput (mL day^{-1})	Yield (mg day^{-1})	Haloalkane (vol frac)	Reagent Use (µL mg^{-1} product)	Energy (Wh g^{-1})
405	1.0	50	18–22	DCM (0.41)	40 ± 5	30–38
460	1.5	100	20–24	DCM (0.39)	41 ± 4	32
480	2.0	150	22–25	DCM (0.37)	40 ± 3	35
570	3.5	200	28–30	2-I-Prop (0.45)	44 ± 4	40
600	4.0	220	30–31	2-I-Prop (0.47)	47 ± 3	43
650	4.8	250	31–32	2-I-Prop (0.50)	49 ± 3	45

S17. Post-Synthesis Band-gap Tuning Comparison of MHP NCs.

Table S12. *Comparison of post-synthetic bandgap-tuning strategies for $CsPbX_3$ nanocrystals*

Approach	Mechanistic Driver	Tunability ($\Delta\lambda_{em}$, nm)	PLQY Retention (%)	FWHM (Linewidth, nm)	Scalability / Throughput	Sustainability / Green-Chemistry Aspect
**Salt-driven halide exchange**[4,5]	Ion diffusion via excess halide salts in polar/nonpolar media	420–640 nm	60–85 % (drops after multiple cycles)	30–45 nm	Batch-limited; poor reproducibility due to mixing heterogeneity	Generates salt-containing effluent; requires purification steps
**Precursor engineering**[6,7]	Controlled halide ratio during nucleation or growth	400–630 nm	80–95 %	25–35 nm	Moderate; dependent on synthesis throughput	Moderate waste; multiple precursor syntheses required
**Thermal diffusion post-treatment**[8]	Thermally driven halide migration	430–600 nm	40–70 %	40–60 nm	Scalable but high energy input; possible lattice degradation	High energy use; thermal decomposition risk
**Photo-induced anion exchange**[1] **(this work)**	Photon-flux-driven halide substitution under flow confinement	405–650 nm (continuous tunability)	90–98 %	20–28 nm	Fully automated; scalable from 10 µL droplets → 250 mL day^{-1} continuous flow	Minimal reagent use; sealed microfluidic system; negligible waste; no purification required

S18. Thermal Stability of the Microfluidic Photoreactor

The aluminum reactor body served as a passive heat sink, maintaining the reactor temperature constant at 23 ± 0.02 °C throughout prolonged (3 h) UV irradiation at a photon flux of 120 pmol s^{-1}, as verified by *in situ* temperature monitoring.

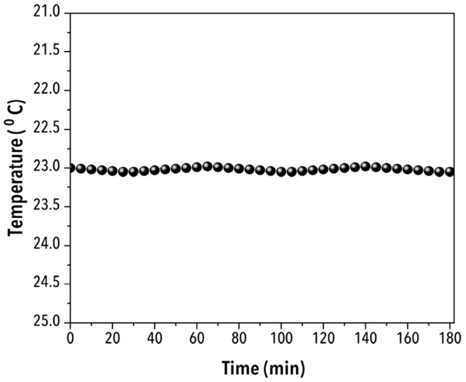

Figure S18. *Thermal stability of the microfluidic photoreactor under prolonged UV irradiation. Microfluidic reactor temperature as a function of time during a 3 h PIAER experiment conducted at a photon flux of 120 pmol s^{-1} (25 mA LED input). The temperature remained stable at 23 ± 0.02 °C, confirming negligible photothermal heating. The aluminum reactor body acts as a passive heat sink, dissipating the UV-induced thermal load during continuous operation.*

Supplementary References

(1) Jha, P.; Mukhin, N.; Ghorai, A.; Morshedian, H.; Canty, R. B.; Delgado-Licona, F.; Brown, E. E.; Pyrch, A. J.; Castellano, F. N.; Abolhasani, M. Photo-Induced Bandgap Engineering of Metal Halide Perovskite Quantum Dots In Flow. *Advanced Materials* **2025**, *37* (16), 2419668. https://doi.org/10.1002/adma.202419668.

(2) Morshedian, H.; Abolhasani, M. Accelerated Photostability Studies of Colloidal Quantum Dots. *Solar RRL* **2023**, *7* (10), 2201119. https://doi.org/10.1002/solr.202201119.

(3) Jha, P.; Mukhin, N.; Ghorai, A.; Morshedian, H.; Canty, R. B.; Delgado-Licona, F.; Brown, E. E.; Pyrch, A. J.; Castellano, F. N.; Abolhasani, M. Photo-Induced Bandgap Engineering of Metal Halide Perovskite Quantum Dots In Flow. *Advanced Materials* *n/a* (n/a), 2419668. https://doi.org/10.1002/adma.202419668.

(4) Akkerman, Q. A.; D'Innocenzo, V.; Accornero, S.; Scarpellini, A.; Petrozza, A.; Prato, M.; Manna, L. Tuning the Optical Properties of Cesium Lead Halide Perovskite Nanocrystals by Anion Exchange Reactions. *J. Am. Chem. Soc.* **2015**, *137* (32), 10276–10281. https://doi.org/10.1021/jacs.5b05602.

(5) Abdel-Latif, K.; Epps, R. W.; Kerr, C. B.; Papa, C. M.; Castellano, F. N.; Abolhasani, M. Facile Room-Temperature Anion Exchange Reactions of Inorganic Perovskite Quantum Dots Enabled by a Modular Microfluidic Platform. *Advanced Functional Materials* **2019**, *29* (23), 1900712. https://doi.org/10.1002/adfm.201900712.

(6) Prasanna, R.; Gold-Parker, A.; Leijtens, T.; Conings, B.; Babayigit, A.; Boyen, H.-G.; Toney, M. F.; McGehee, M. D. Band Gap Tuning via Lattice Contraction and Octahedral Tilting in Perovskite Materials for Photovoltaics. *J. Am. Chem. Soc.* **2017**, *139* (32), 11117–11124. https://doi.org/10.1021/jacs.7b04981.

(7) Ramos-Terrón, S.; Martín, C.; Miguel, G. de; Solano, E.; Hermida-Merino, D.; Vondel, J. V. de; Hofkens, J.; Keshavarz, M. A-Site Cation Engineering and Halide Tuning via Precursor Engineering to Tune the Optical Properties of 2D Perovskites. *RSC Adv.* **2025**, *15* (34), 28181–28190. https://doi.org/10.1039/D5RA03422A.

(8) Yamauchi, M.; Fujiwara, Y.; Masuo, S. Slow Anion-Exchange Reaction of Cesium Lead Halide Perovskite Nanocrystals in Supramolecular Gel Networks. *ACS Omega* **2020**, *5* (24), 14370–14375. https://doi.org/10.1021/acsomega.0c00880.
