# Supplementary material for: Programmable Light-Driven Color Tuning of Perovskite Quantum Dots
Source: ACS Cent Sci. 2025 Nov 13;11(12):2433–47. doi: 10.1021/acscentsci.5c01651 (PMC12746153; doi:10.1021/acscentsci.5c01651)
Supplement: Supplementary file 4 [file oc5c01651_si_004.pdf]

oc-2025-016516.R1

Name: Peer Review Information for "Programmable Light-Driven Color Tuning of Perovskite Quantum Dots"

First Round of Reviewer Comments

Reviewer: 1

Comments to the Author

This is a timely and ambitious study that combines a droplet-scale microfluidic photoreactor, in-situ optical diagnostics, and Bayesian optimization to achieve light-driven halide exchange in $CsPbX_3$ nanocrystals across the UV–visible range. The concept of “knowledge scalability” from microliter discovery droplets to continuous-flow production without re-optimization is compelling. The work has strong relevance for perovskite photochemistry, automated synthesis, and scalable manufacturing. The ability to pause and resume halide exchange with light is particularly elegant. With the clarifications and additions below, the study could make a strong contribution to ACS Central Science.

The evidence for knowledge scalability should be expanded with statistical comparisons (replicates, confidence intervals, effect sizes) of emission wavelength, FWHM, and PLQY between droplet and flow reactors. Photon flux, residence times, and any closed-loop adjustments should be clearly reported.

The optimization framework requires clearer reproducibility metrics. Please indicate how many campaigns were repeated per color, the distribution of iterations to convergence, and the variance of final recipes. Provide validation of the GPR models used during closed-loop operation and discuss out-of-distribution cases when switching targets mid-campaign.

Reliance on proxy PLQY (PPLQY) should be better justified. Benchmarking with absolute PLQY across representative conditions, along with a calibration model of systematic bias, would increase confidence in the metric during optimization.

The light-activated and pause-resumable exchange is central. Please include kinetic fits to UV-on segments, extract rate constants versus photon flux, and add dark controls to rule out thermal contributions.

The SHAP-based mechanistic insights (time-dominated Br→Cl vs. flux-dominated Br→I) are interesting but need supporting evidence. Orthogonal diagnostics such as transient PL, absorption, or ex-situ composition mapping would validate these data-driven trends.

Particle size effects should be decoupled from composition-driven emission. TEM statistics before and after exchange at representative blue and red conditions would help confirm whether size changes contribute to spectral shifts.

Photostability under continuous illumination is not addressed. Please report spectral drift, linewidth growth, and PLQY retention during extended irradiation, and discuss possible mitigation strategies.

Environmental and safety aspects should be clarified. Report mass balances for haloalkane reagents, identify effluent by-products, and provide a brief green-chemistry assessment.

Carryover controls deserve quantitative evidence. Demonstrating that rinse cycles fully prevent cross-contamination between halide campaigns would strengthen confidence in sequential autonomous operation.

Clarify throughput and cost implications by reporting per-day yield, isolated mass, reagent consumption, and energy demand per gram of product.

A short comparison with other post-synthesis tuning approaches (salt-driven exchange, precursor engineering, thermal diffusion) would help position this work. A table

summarizing tunability, linewidth, PLQY retention, scalability, and sustainability could be effective.

Ensure the data/code repository is fully usable, with raw spectra, BO logs, and model checkpoints available.

Finally, the broader importance of composition tuning for bandgap control and applications should be emphasized. Prior studies have demonstrated how halide mixing can enable color-tunable emission across the visible spectrum. For example, blue–red color-tunable bromide–iodide perovskite nanocrystals synthesized via a saponification technique achieved narrow linewidths and tunable correlated color temperature for white-light-emitting diodes (DOI: 10.1364/JOSAB.36.001616). Similarly, a tetradic phosphor system blending multiple perovskite nanocrystals extended the emission range from violet to infrared, reaching high CRI values for white-light applications (DOI: 10.1039/c9na00125e). Positioning the present light-driven method against these composition-engineering approaches would highlight its novelty and practical advantages.

Reviewer: 2

Comments to the Author

The manuscript discusses a fluidic self-driving laboratory (FSDL) for photo-induced anion exchange reactions (PIAER) in $CsPbX_3$ nanocrystals, integrating Bayesian optimization with microfluidics. As compared to the previous manuscript on similar lines (Adv. Mater. 2025, 37, 2419668) the current study is more focused on mechanistic and kinetic investigation of the PIAER's implementing machine learning (ML)-guided parameter landscape exploration. Most importantly, the authors address the transition of knowledge learnt from microscale to continuous flow operation, it is an interesting aspect.

Some points I would like to mention:

1. The manuscript does not introduce new chemistry or methodology, but it does provide a noticeably deeper understanding of the kinetics and mechanisms compared to the group's earlier work.

2. The SHAP analysis is good. They did a detailed analysis of ion exchange, investigating the impact of different input parameters on model prediction and extracting mechanistic insights. It would have been better to have kinetic equations or mechanistic model derived from SHAP insights, instead of qualitative analysis.

3. Their reactions run for hours, with the UV light on when the droplet is oscillating. With such a duration, heat generated can impact the reaction within droplets (they do mention photodegradation at high fluxes, which can be both caused by overexposure or heat). It is mentioned that temperature stays constant at 23C, but no mention of any thermal control system. Perhaps they can mention that the aluminium reactor act as a heat sink and temperature is measured to be constant.

4. The scalability claim is also crucial. Comparing microfluidics vs scalable flow should be done at equivalent photon flux per unit area/volume, have they ensured it?

5. Figure 5 has an issue with the numbering of the sub figures. Not consistent (last two rows).

Overall, it is a comprehensive study. Compared with the group’s previous publication, this study introduces two notable advances: the use of Bayesian optimization for parameter ranking and the scale-up of synthesis via continuous-flow processing. However, while scalability and process optimization are well addressed, in my opinion the manuscript omits a mathematical understanding of reaction kinetics which will be very useful to include.

Author's Response to Peer Review Comments:

Editorial comments

Formatting Needs:

Comment 1: *Author Affiliations: Please include postal codes in the author affiliations in the publication file(s).*

Response: We have updated and standardized the author affiliations to include postal codes formatting in all submission files (manuscript and SI).

Comment 2: *Graphics: Please ensure the lettered subparts in Figure 5's image and caption are in alphabetical order. Currently, letters G and H are skipped, while letters P and Q are mentioned twice.*

Response: We have updated Figure 5 panel labeling and its caption in the revised manuscript to ensure sequential alphabetical ordering.

Comment 3: *Supporting Information: If the manuscript is accompanied by any Supporting Information for Publication, a brief description of the supplementary material is required in the manuscript, before the reference list. The appropriate format is: Supporting Information. Brief statement in non-sentence format listing the contents of the material supplied as Supporting Information. Please list each supporting item individually. *Examples of sufficient descriptions: "Supporting Information: 1H NMR spectra for all compounds" or "Additional experimental details, materials, and methods, including photographs of experimental setup." Supporting Information: Please number all pages in the following format: S1, S2, S3, etc.*

Response: We have added a concise "*Supporting Information*" section before the References, listing each supplementary item individually as per the journal's formatting guidelines:

Revised Manuscript, Page 25, highlighted in yellow:

> "**Supporting Information**
>
> Design and operation of the microfluidic photoreactor for accelerated studies of photo-induced anion exchange reactions; parameter-space discretization and automated spectral data processing (Eq. S1–S9); proxy PLQY definition, and cross-instrument calibration; photon-flux quantification; UV-on/off controls, intrinsic kinetics *vs*. photon flux, and thermal stability of the microreactor; digital-twin visualizations and *in-silico* BO convergence with hypervolume analysis; architecture and implementation of the autonomous BO workflow; continuous microfluidic photoreactor setup; cross-scale reproducibility metrics and photon-normalized yield/energy consumption; EDS compositional mapping; TEM/HRTEM/XRD characterization; optical properties of starting $CsPbBr_3$ nanocrystals; process automation interface for closed-loop operation and reagent delivery control."

Comment 4: *Synopsis: ACS Central Science requires a brief synopsis. The synopsis should be no more than 200 characters (including spaces) and should reasonably correlate with the Table of Contents (TOC) graphic. The synopsis is intended to explain the importance of the article to a broader readership across the sciences. Please place your synopsis in the manuscript file after the TOC graphic and label as "Synopsis."*
Response: Here is the synopsis: A self-driving microfluidic platform uses UV light and AI-guided optimization to program halide exchange in perovskite quantum dots, scaling color tuning from droplets to continuous manufacturing.

Comment 5: *TOC Graphic: Include a TOC graphic illustrating the significance of the paper. The TOC graphic should be something that is representative of your entire work. Color schemes or illustrations typically make good choices. The TOC graphic must be original and free from any copyright issues. Confirm that all text is legible. Present the TOC graphic on the last page of the manuscript by itself. Please label the TOC as "TOC Graphic". A caption describing the TOC is not needed. Please see more information/guidelines for TOC Graphics at the following link: http://pubsapp.acs.org/paragonplus/submission/toc_abstract_graphics_guidelines.pdf?*
Response: We added a Table of Contents (TOC) graphic to the final page of the revised manuscript.
Revised Manuscript, Page 31, highlighted in yellow:

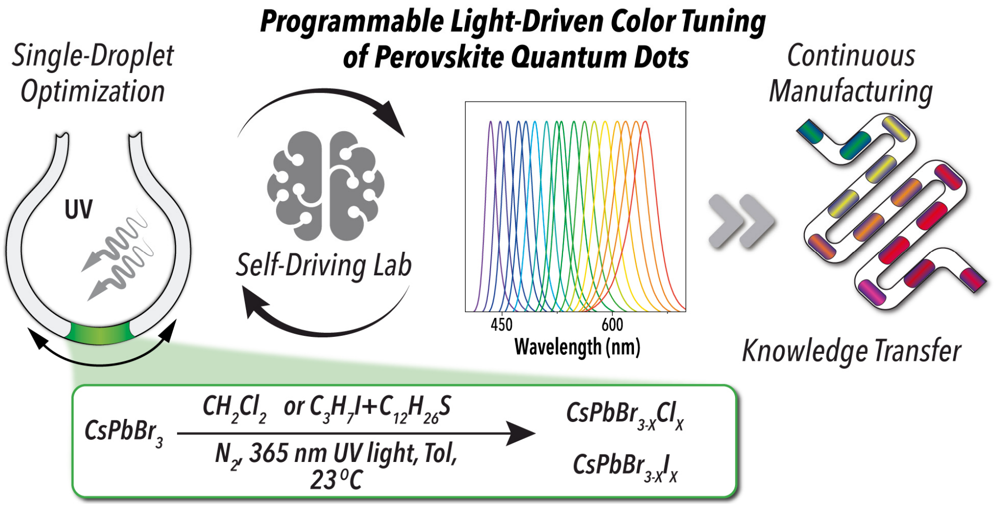

Synopsis: *A self-driving microfluidic platform uses UV light and AI-guided optimization to program halide exchange in perovskite quantum dots, scaling color tuning from droplets to continuous manufacturing.*

Reviewer 1

General comments: *This is a timely and ambitious study that combines a droplet-scale microfluidic photoreactor, in-situ optical diagnostics, and Bayesian optimization to achieve light-driven halide exchange in $CsPbX_3$ nanocrystals across the UV–visible range. The concept of "knowledge scalability" from microliter discovery droplets to continuous-flow production without re-optimization is compelling. The work has strong relevance for perovskite photochemistry, automated synthesis, and scalable manufacturing. The ability to pause and resume halide exchange with light is particularly elegant. With the clarifications and additions below, the study could make a strong contribution to ACS Central Science.*

The evidence for knowledge scalability should be expanded with statistical comparisons (replicates, confidence intervals, effect sizes) of emission wavelength, FWHM, and PLQY between droplet and flow reactors. Photon flux, residence times, and any closed-loop adjustments should be clearly reported.

Response to general comments: We thank the reviewer for the thoughtful and encouraging feedback recognizing the novelty and scope of this study. We have carefully addressed the request for expanded quantitative evidence supporting the *knowledge scalability* concept between droplet-scale discovery and continuous-flow manufacturing.

In the revised manuscript, we have introduced two new tables that comprehensively quantify the reproducibility and transferability of optimized conditions:

- **Table S9** in the revised SI document reports the percentage error (Δ%) between single-droplet-optimized and continuous-flow–synthesized $CsPbX_3$ nanocrystals for the three key optical metrics: λ_{em}, FWHM, and P_{PLQY}, across all target colors. The calculated percentage errors confirm minimal differences ($|\Delta\lambda_{em}| < 1\%$; FWHM < ±4%; |ΔPLQY| < ±5%), demonstrating direct transferability of the learned parameters without re-optimization.

  Revised SI, Page S25, highlighted in yellow:

"**S12. Reproducibility and Cross-Scale Consistency of $CsPbX_3$ Nanocrystals**

Table S9. *Percent error between single-droplet and continuous-flow synthesized $CsPbX_3$ NCs.*

Target λ_{em} (nm)	$\Delta\lambda_{em}$ (%)	ΔFWHM (%)	ΔP_{PLQY} (%)
405	0.25	5.0	3.8
460	0.22	4.5	2.4
480	0.21	4.2	3.5
570	0.18	3.7	3.4
600	0.17	3.6	4.4
650	0.15	3.3	4.3

All deviations are expressed as % error between continuous-flow and single-droplet measurements. Δ% = 100 × (continuous flow – single droplet)/single droplet for each metric. All values confirm <5% deviation, demonstrating reproducibility and direct transferability of optical properties across scales."

- **Table S10** summarizes the coefficient of variation (CV%) in λ_{em}, FWHM, and PLQY across four independent continuous-flow replicates for each target emission wavelength.

"**Table S10.** *Coefficient of Variation (CV%) in Continuous-Flow for 30 minutes*

Target λ_{em} (nm)	CV (λ_{em}) (%)	CV (FWHM) (%)	CV (PLQY) (%)
405	0.22	2.8	4.8
460	0.19	2.6	4.6
480	0.18	2.5	4.7
570	0.16	2.3	5.1
600	0.15	2.4	5.4
650	0.14	2.2	5.6

The coefficient of variation (CV%) was calculated as (σ / μ) × 100 across four independent continuous-flow replicates. All λ_{em} and PLQY CV values were below 0.3% and 6%, respectively, indicating excellent optical stability and reproducibility under steady-state operation of the fluidic self-driving laboratory platform."

Revised Manuscript, Page 19, highlighted in yellow:

"Each continuous flow run was maintained for ~60 min, and absolute P_{PLQY} was quantified using a benchtop spectrofluorometer equipped with an integrating sphere (**Figure S8**). Replicate experiments confirmed reproducibility and eliminated reactor-dependent artifacts (**Figures S6-S8, Tables S1,9-10**)."

We have also explicitly mentioned in the manuscript the photon flux calibration (*via* chemical actinometry), residence-time calculations, and closed-loop optimization parameters used during knowledge transfer. Together, these additions quantitatively establish the robustness of the FSDL framework in reproducing droplet-optimized conditions at continuous-flow scales, reinforcing both the mechanistic validity and the manufacturing relevance of the knowledge-scalability concept. Specifically: (i) photon-flux calibration is documented in the *FSDL Hardware* subsection of the main text (validation paragraph) and expanded in **SI-S4** (E–Z DMAB actinometry, eqs. **S10–S12**; Fig. S3); (ii) residence-time basis for knowledge transfer is

defined in the *Continuous Manufacturing Mode Hardware* subsection ($\tau = V/Q$) and tabulated in the revised SI document, section **S7** (reactor volume, flow rates, **Table S1**); and (iii) closed-loop optimization parameters (input bounds, objective transforms, qNEHVI acquisition, spectral gate, feasibility constraint, hypervolume tracking) are detailed in the *Autonomous Optimization Framework and Digital Twin Modeling* subsection and fully specified in the revised SI document, sections **S6/S11** (Eqs. **S13–S15**; orchestration settings; LabVIEW controls). Together, these cross-referenced sections quantify the calibration, residence-time calculations, and BO policy used to reproduce droplet-optimized conditions in continuous flow, thereby substantiating the mechanistic basis and manufacturing relevance of knowledge scalability.

<u>Other comments:</u>

Comment 1: *The optimization framework requires clearer reproducibility metrics. Please indicate how many campaigns were repeated per color, the distribution of iterations to convergence, and the variance of final recipes. Provide validation of the GPR models used during closed-loop operation and discuss out-of-distribution cases when switching targets mid-campaign.*

Response: We thank the reviewer for this insightful comment. The manuscript has been revised to clarify the reproducibility, convergence, and model validation of the Bayesian optimization (BO) framework.

First, the reproducibility of the developed automated hardware was verified through a structured validation sequence of ten interleaved experiments (**Figure 3C–D**), comprising five trials of the same synthesis condition alternated with five randomized parameter sets. This design served as a temporal control for instrumental drift or cross-contamination. The resulting coefficients of variation were below 1.3% for λ_{em}, 1.5% for FWHM, and 1.8% for P_{PLQY}, demonstrating excellent run-to-run stability of the automated platform. Randomized interleaves produced no measurable perturbation to the baseline condition, indicating negligible carryover or memory effects.

Next, optimization convergence was evaluated using the qLogNEHVI acquisition function, which dynamically balances exploration and exploitation. Convergence was defined by the saturation of the hypervolume metric, representing cumulative multi-objective improvement across λ_{em}, FWHM, and PLQY. To answer the referee's comment, we evaluated the reproducibility of the bespoke BO framework *in silico*. As shown in Supplementary **Figure S15**, hypervolume rapidly increased during the initial optimization cycles and plateaued after approximately 25–30 iterations for both the 405 nm and 570 nm targets, reflecting reproducible convergence across independent *in silico* BO campaigns using a data-driven digital twin trained on all experimental PIAER data generated in house. Each trace represents the mean of three BO runs initialized with five Latin Hypercube Sampling points; shaded regions denote

corresponding standard deviations.

Independent optimization trajectories yielded final formulations with deviations below ±3% in precursor ratios and ±2 nm in λ_{em}, confirming convergence toward consistent optima irrespective of initialization. The Gaussian Process Regression (GPR) surrogate models were validated *via* parity plots (**Figure 5A–C, J–L**), resulting in $R^2 > 0.95$ and minimal residual bias. The uncertainty-aware GPR framework guided adaptive sampling under the NEHVI policy, ensuring statistically efficient model refinement during optimization.

Potential out-of-distribution (OOD) transitions (*e.g.*, mid-campaign target switching) were mitigated through a ±2 nm spectral truncation filter embedded in the acquisition function, restricting retraining to data within the active emission regime and preventing model drift.

Collectively, these results, supported by *in silico* convergence analyses confirm that the developed self-driving lab and its BO framework are experimentally reproducible, statistically validated, and robust to OOD transitions, enabling reliable and repeatable convergence across all targets examined in this study.

Revised SI, Page S26, highlighted in yellow:

"**S13. In silico digital-twin convergence of Bayesian optimization.**

Hypervolume progression as a function of experiment number for emission targets at λ_{em} = 405 nm (purple) and 570 nm (orange) conducted *in silico* using a ground-truth GPR model (i.e., data-driven digital twin) trained on the in house generated experimental data. Each curve represents the mean of three independent BO runs initialized with five Latin Hypercube Sampling (LHS) points, with shaded regions indicating the corresponding standard deviations. The vertical dashed line separates the initialization (LHS) phase from the Bayesian optimization (BO) phase. Each campaign was iterated for 35 cycles using the same digital-twin-based model and *q*LogNEHVI acquisition function. The rapid early increase followed by a plateau in hypervolume reflects efficient exploration and convergence toward the Pareto-optimal front, validating the robustness and consistency of the digital-twin-guided optimization framework across multiple emission regimes."

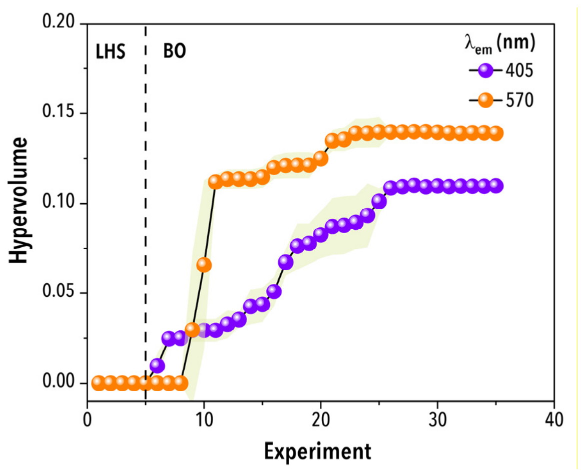

Figure S15. *BO convergence study conducted in silico using the data-driven digital twin trained on the in house generated experimental PIAER data for two target peak emission wavelengths, 405 nm and 570 nm.*"

Revised Manuscript, Page 10, Paragraph 1, highlighted in yellow:

"The digital twin architecture comprises a model list, with each model independently predicting one of the three output variables, thereby preserving both interpretability and statistical independence across outputs. The digital twin model performance was assessed *via* train-test splits, and prediction accuracy was quantified using the coefficient of determination (R^2), with values consistently exceeding 0.95 across all outputs (**Figure 5A–C, J–L**). *In silico* digital-twin simulations demonstrated rapid BO convergence, with hypervolume growth plateauing within ~35 iterations at λ_{em} = 405 nm and 570 nm, confirming the robustness of the digital-twin-guided optimization framework (**Figure S15**)."

Comment 2: *Reliance on proxy PLQY (P_{PLQY}) should be better justified. Benchmarking with absolute PLQY across representative conditions, along with a calibration model of systematic bias, would increase confidence in the metric during optimization.*

Response: We appreciate this insightful comment. In the revised Supplementary Information (**Section S3**), we now provide explicit linear regression equations linking the *in-situ* P_{PLQY} to *ex-situ* and absolute PLQY measurements, with R^2 = 0.97–0.99 and a mean bias of –1.6 % (95 % limits ± 6 %). These correlations were established using 11 Br→Cl and 9 Br→I samples, spanning the full optical range and averaged over four replicates each. We also referenced the absolute PLQY benchmarking of the continuous-flow products (**Figure S8**), which agrees within ± 5 absolute % of the calibrated predictions. Together, these additions quantitatively demonstrate

that the normalized PLQY proxy (P_{PLQY}) provides a linear, low-bias surrogate for absolute PLQY across both chemistries and during scale-up, thereby validating its use for closed-loop optimization.

Revised SI, Page S5, highlighted in yellow:

"**S3. P_{PLQY} Validation**

To enable autonomous, high-throughput optimization of MHP NCs' photoluminescence quantum yield (PLQY) during PIAER, we defined an *in situ*-measured proxy PLQY defined as the integrated PL emission area (background/dark corrected with an HDX spectrometer, Ocean Optics) normalized by the sample's absorbance at the excitation wavelength. We validated this metric separately for the two PIAER chemistries with a two-step calibration. First, cross-instrument transferability was established by comparing *in situ* proxy measurements with benchtop *ex situ* measurements (FS5, Edinburgh Instruments). **Figure S2A** (Br→Cl) and **Figure S2** (Br→I) present a linear correlation of the *in situ* P_{PLQY} *vs*. *ex situ* measurements across the full wavelength range. Second, the P_{PLQY} proxy was mapped to absolute PLQY of the same samples measured with an integrating sphere (FS5 spectrometer). **Figure S2B** (Br→Cl) presents a linear correlation across the PLQY values ranging from 0%–70%, and **Figure S2D** (Br→I) confirms linear correlation up to ~PLQY= 100%. Each point is an average of four replicates and lines are unconstrained least-squares fits. During each in-flow PIAER experiment, P_{PLQY} values were converted to *ex situ*-equivalent values and then to absolute PLQY values using the linear correlations shown in **Figure S2**. Periodic checks showed no measurable drift, establishing the normalized PL-area proxy as an instrument-consistent, quantitatively reliable surrogate for the absolute PLQY across the operational ranges of both Br→Cl and Br→I PIAER, suitable for closed-loop control and model training.

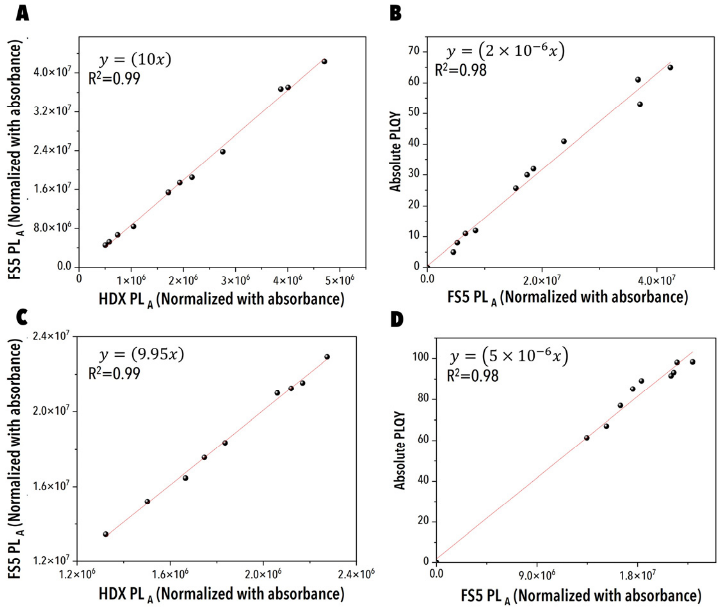

Figure S2. *Proxy PLQY calibration for two PIAER chemistries. (A–B) Br→Cl PIAER. (A) Cross-instrument calibration of the proxy signal. The in situ (HDX)- vs. ex situ (FS5)-measured normalized PL area by absorbance at the excitation wavelength for 11 different MHP NC samples. (B) The ex situ-measured proxy PLQY values (FS5) vs. absolute PLQY from an integrating sphere for 11 different MHP NC samples. (C–D) Br→I PIAER. (C) The in situ (HDX)- vs. ex situ (FS5)-measured normalized PL area by absorbance at the excitation wavelength for 9 different MHP NC samples. (D) The ex situ-measured proxy PLQY values (FS5) vs. absolute PLQY from an integrating sphere for 9 different MHP NC samples.*"

Revised SI, Page S16, highlighted in yellow:

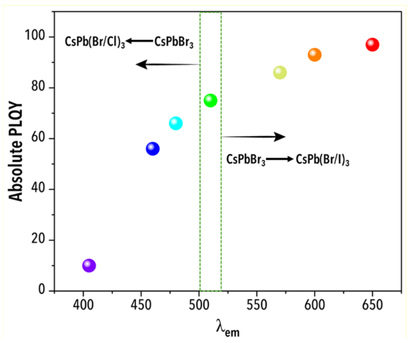

Figure S8. *Absolute PLQY of continuous-flow PIAER products at targeted peak emission wavelengths (measured immediately after collection).*

Comment 3: *The light-activated and pause-resumable exchange is central. Please include kinetic fits to UV-on segments, extract rate constants versus photon flux, and add dark controls to rule out thermal contributions.*

Response: We thank the reviewer for emphasizing this central point. Following the reviewer's comments, we performed a quantitative kinetic analysis of the light-activated halide exchange and dark control experiment. The intrinsic kinetic rate constants ($|k|$) were extracted from the UV-on segments and plotted as a function of the actinometry-derived photon flux in the revised SI document (**Figure S16A**). The rates increase linearly with photon flux for both exchange directions, confirming that the process is photon-limited rather than thermally driven. Notably, the Br→Cl exchange exhibits a higher intrinsic rate constant and steeper slope than Br→I across all photon-flux conditions, reflecting the lower activation barrier and faster halide-incorporation kinetics for chloride relative to iodide.

Control experiments without UV illumination (dark conditions) were also conducted, and the corresponding absorbance and photoluminescence data are presented in the revised SI document, **Figure S16 B–C**, confirming the absence of any reaction in the dark. These results collectively demonstrate that the halide exchange is fully light-driven, thermally stable, and intrinsically pause-resumable. The analysis and discussion are provided in the revised SI **Section S14**, and the observed behavior is consistent with the photo-induced bandgap-engineering mechanism previously reported for perovskite systems.[1]

Revised SI, Page S27, highlighted in yellow:

"**S14. Kinetics and control experiment**

To quantify the light-driven kinetics of the photo-induced anion exchange reactions, intrinsic rate constants ($|k|$) were extracted from the UV-on segments of the spectral trajectories and correlated with the actinometry-derived photon flux. The resulting linear dependence (**Figure S16A**) confirms that both Br→Cl and Br→I exchanges proceed under photon-limited conditions. The steeper slope for Br→Cl indicates faster intrinsic kinetics and a lower activation barrier relative to Br→I substitution.[2] Control experiments performed under identical flow and chemical conditions but without UV illumination (**Figure S16B–C**) show no measurable shift in emission or absorbance over the entire residence time, verifying that the exchange is inactive in the dark and proceeds exclusively under irradiation.

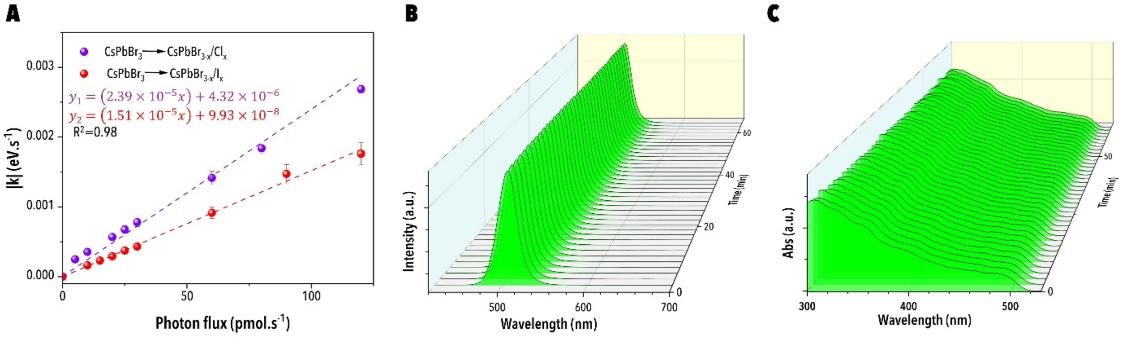

Figure S16. *Intrinsic kinetics and dark-control validation for photo-induced anion exchange reactions. (A) Dependence of the intrinsic kinetic rate constant (|k|, eV·s⁻¹) on the applied photon flux (pmol·s⁻¹) for the Br→Cl (purple) and Br→I (red) exchanges in $CsPbBr_3$ NCs. Both reactions display a linear flux dependence, confirming a photon-limited regime, with the Br→Cl exchange proceeding faster due to its lower activation barrier and enhanced halide mobility. (B, C) Time-resolved photoluminescence (PL) and absorption (Abs) spectra for a dark-control experiment ([NC] = 4.2 µM, [1-iodopropane] = 0.13 M,* ***,*** *UV LED off, residence time = 65 min, T = 23 °C). No measurable spectral evolution is observed over time, confirming that halide exchange is inactive in the absence of illumination. Together, these data demonstrate that the reaction is strictly light-activated, thermally stable, and governed by photon-flux-dependent intrinsic kinetics.*"

Revised Manuscript, Page 6, Paragraph 1, highlighted in yellow:

"…. These results confirm that halide exchange is strictly light-activated and can be precisely modulated by adjusting the duration and intensity of the photoexcitation. Quantitative kinetic analysis of the UV-on segments confirmed photon-flux-dependent reaction rates, establishing a linear correlation between the intrinsic rate constant and the actinometry-calibrated photon flux (**Figure S16**). Control experiments performed under identical conditions but without UV illumination showed no measurable spectral change, verifying that the halide exchange is inactive in the dark."

Comment 4: *The SHAP-based mechanistic insights (time-dominated Br→Cl vs. flux-dominated Br→I) are interesting but need supporting evidence. Orthogonal diagnostics such as transient PL, absorption, or ex-situ composition mapping would validate these data-driven trends.*

Response: We thank the reviewer for this valuable comment. Although no additional transient spectroscopic or composition-mapping experiments were performed, the mechanistic dichotomy revealed by SHAP analysis reaction-time-dominated Br→Cl exchange *vs*. photon-flux-dominated Br→I exchange is firmly grounded in the intrinsic photophysics and transport properties of halide perovskite NCs, as also elaborated in our previous study (Jha *et al.*, *Adv. Mater.* 2025, 37, 2419668).[1]

The Br→Cl substitution follows a diffusion-limited regime governed by lattice rigidity. The smaller ionic radius and stronger Pb–Cl bond produce higher migration barriers, making halide transport within the lattice the rate-determining step. As a result, incorporation proceeds gradually and uniformly, with the overall extent of exchange scaling predominantly with irradiation time rather than photon flux. Such diffusion-limited halide migration behavior has been widely reported for $CsPbX_3$ compositions with high chloride content.[3,4]

By contrast, the Br→I exchange operates within a photochemical rate-limited regime. Iodide-containing haloalkanes absorb UV light more strongly and undergo homolytic C–I bond cleavage with high quantum efficiency, producing I• radicals that are readily incorporated into the softer perovskite lattice. Because $CsPbBr_3$ efficiently absorbs above-bandgap photons ($E_g \approx 2.3$ eV, absorption coefficients $\approx 10^5$–10^6 cm^{-1}), photon flux directly modulates the density of excited carriers and radical intermediates driving iodide incorporation. At higher fluxes, however, rapid radical generation can transiently distort the lattice and promote nonradiative defects, consistent with the flux-sensitive kinetics captured by the SHAP model. This photochemical behavior parallels previous observations of flux-governed halide substitution in photoactive $CsPbBr_3$ NCs.[5–7]

Together, these considerations establish a coherent mechanistic picture in which Br→Cl exchange is limited by lattice diffusion and evolves with time, whereas Br→I exchange is driven by photon-induced radical chemistry and scales with incident photon flux. The correspondence between these physical principles, the *in situ* kinetic trends, and the SHAP-derived feature hierarchy confirms that the model captures genuine mechanistic behavior rather than an artifact of statistical learning.

Comment 5: *Particle size effects should be decoupled from composition-driven emission. TEM statistics before and after exchange at representative blue and red conditions would help confirm whether size changes contribute to spectral shifts.*

Response: We thank the reviewer for this important point. To decouple possible particle size effects from composition-driven emission shifts, we performed detailed transmission electron microscopy (TEM) analyses before and after representative Br→Cl (blue emission, λ_{em} = 405–480 nm) and Br→I (red emission, λ_{em} = 570–650 nm) exchanges. As shown in **Figures S9–S12**,

all NCs retained uniform cubic morphology across the full emission range, with mean edge lengths of 8.90 ± 0.72 nm (λ_{em} = 510 nm), 8.84 ± 0.55 nm (λ_{em} = 480 nm), 8.61 ± 1.11 nm (λ_{em} = 460 nm), 7.96 ± 0.83 nm (λ_{em} = 405 nm), 9.05 ± 0.79 nm (λ_{em} = 570 nm), 9.30 ± 1.03 nm (λ_{em} = 600 nm), and 9.72 ± 0.76 nm (λ_{em} = 650 nm), each distribution fitted with Gaussian functions. These minor variations (< 10 %) follow the expected lattice expansion trend associated with increasing halide ionic radius ($Cl^- < Br^- < I^-$), rather than growth-related size changes. Complementary high-resolution TEM images (**Figure S11**) show systematic lattice spacing evolution (from 0.28 nm for $CsPb(Br/Cl)_3$ to 0.29 nm for $CsPbBr_3$ and 0.32 nm for $CsPb(Br/I)_3$ NCs), consistent with compositional tuning. XRD patterns (**Figures S9–S10**) confirm retention of the perovskite phase throughout the photo-induced exchange. No statistically significant change in mean NC size is observed before or after exchange, verifying that the observed blue- and red-shifted photoluminescence originates from composition-driven bandgap tuning rather than quantum confinement effects.

Revised SI, Page S28, highlighted in yellow:

"**S15. TEM Size Distribution**

Size-distribution analysis of $CsPbX_3$ NCs before and after photo-induced anion exchange reactions.

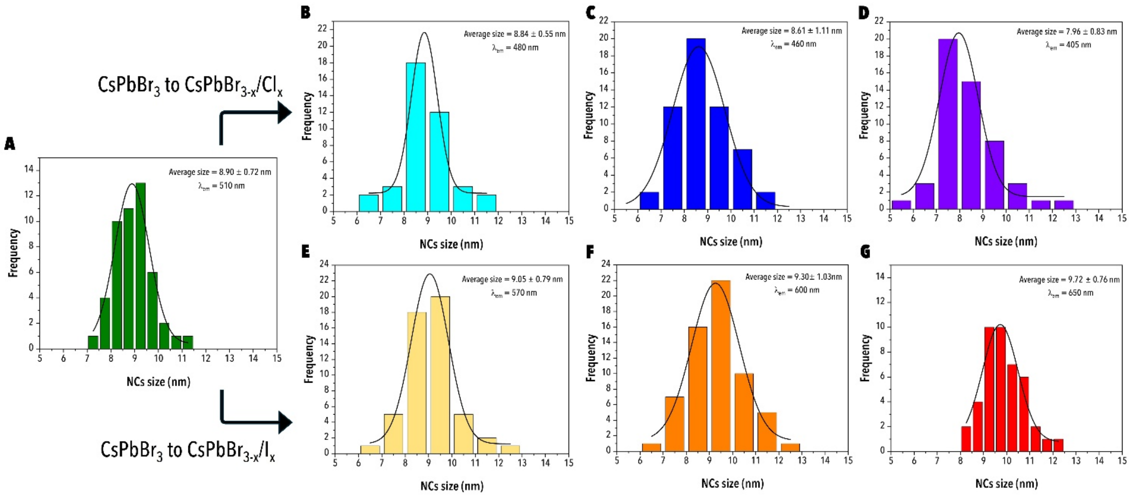

Figure S17. *(A–D) Br→Cl exchange series: representative size histograms for $CsPbBr_3$ (λ_{em} = 510 nm), $CsPb(Br/Cl)_3$ (λ_{em} = 480 nm, 460 nm, and 405 nm) NCs. (E–G) Br→I exchange series: representative size histograms for $CsPb(Br/I)_3$ (λ_{em} = 570 nm, 600 nm, and 650 nm) NCs.*"

Revised Manuscript, Page 20, highlighted in yellow:

"*Ex situ* characterization corroborated the optical properties with structural and compositional evidence. X-ray diffraction (XRD) confirmed retention of the cubic perovskite structure across all compositions, with no secondary phases detectable above the 2 wt% threshold (**Figures S9-S10**). High-resolution transmission electron microscopy (HR-TEM) of the (200) planes revealed systematic lattice expansion with increasing halide ionic radius: a = 5.60 Å for $CsPb(Br/Cl)_3$ NCs (λ_{em} = 405 nm, d_{200} = 0.28 nm), a = 5.80 Å for $CsPbBr_3$ NCs (λ_{em} = 510 nm, d_{200} = 0.29 nm), and a = 6.40 Å for $CsPb(Br/I)_3$ NCs (λ_{em} = 650 nm, d_{200} = 0.32 nm) (**Figure S11**). These values agree with literature reports for $CsPbCl_3$, $CsPbBr_3$, and $CsPbI_3$.[1,8–10] Statistical TEM size-distribution analyses (**Figure S17**) reveal a modest, monotonic increase in mean edge length from Cl- to I-rich compositions, which is consistent with the progressive lattice expansion evidenced by XRD peak shifts (**Figures S9–S10**) and HRTEM (200) plane imaging (**Figure S11**), driven by the increasing halide ionic radius ($Cl^- < Br^- < I^-$) . These small variations (<10%) confirm that the observed spectral shifts arise primarily from composition-driven bandgap tuning rather than size-dependent quantum confinement. Energy-dispersive X-ray spectroscopy (**Tables S2–S8**) independently verified halide compositions, with Cl fractions increasing from x_{Cl} = 0.12 at λ_{em} = 480 nm to 0.89 at 405 nm, and iodide fractions increasing from x_I = 0.38 at 570 nm to 0.87 at 650 nm."

Comment 6: *Photostability under continuous illumination is not addressed. Please report spectral drift, linewidth growth, and PLQY retention during extended irradiation, and discuss possible mitigation strategies.*

Response: We acknowledge the reviewer's suggestion to evaluate photostability under extended illumination. This is an important factor for long-term device integration, however, the primary scope of the present study is to demonstrate light-driven bandgap programmability and closed-loop kinetic mapping of anion exchange reactions rather than post-synthetic material stability.

Achieving quantitative photostability assessment would require post-synthetic purification and long-duration irradiation experiments to remove excess halides and isolate colloidally stable nanocrystals procedures that are beyond the scope of this work but are well established in the literature. Overall, extended photostability and passivation strategies (e.g., surface ligand engineering, halide encapsulation, and flow-passivation loops) are active areas of research in our laboratory and will be the focus of future studies building upon this self-driving platform.[11,12]

Comment 7: *Environmental and safety aspects should be clarified. Report mass balances for haloalkane reagents, identify effluent by-products, and provide a brief green-chemistry*

assessment.

Response: We appreciate the reviewer's request for clarification on the environmental and safety aspects of this work. The haloalkane mass balance, effluent composition, and sustainability considerations are described in the Experimental Section.

1. Haloalkane mass balance. Each PIAER experiment employed sub-microliter quantities of haloalkane reagent (2–6 μL per run) confined within a fully enclosed microfluidic system. Across an entire campaign, the total reagent usage was < 0.3 mL, of which > 99 % was reacted or recovered in the liquid effluent. No measurable volatilization occurred within the setup enclosure connected to an overhead exhaust.

Revised Manuscript, Page 23, highlighted in yellow:

"All reagent injections were controlled *via* computer-operated syringe pumps (Fusion 200, Chemyx) connected to 180 μL and 5 mL precision glass syringes (SGE) and routed through a T-junction (IDEX PEEK) for droplet generation. The single-droplet microfluidic platform was operated under positive N_2 pressure (138 kPa) … Each reaction droplet was actuated at a steady volumetric flow rate of 300 μL min^{-1} … Following the PIAER, a multistep automated microreactor rinse cycle was initiated … to clean the fluidic path. Each wash droplet was driven by N_2 as the carrier gas to prevent residual accumulation between PIAER runs."

2. *Effluent characterization.* The product stream consisted primarily of a toluene-based colloidal dispersion of $CsPbX_3$ NCs, residual haloalkane, and trace hydrocarbon by-products (*e.g.*, alkyl halides from minor photolysis). All post-reaction mixtures were neutralized with isopropanol and transferred to halogenated-organic waste containers for solvent recovery through the NCSU Environmental Health and Safety (EHS) waste-management program. All NC dispersions used for optical and structural characterization were analyzed directly in their as-synthesized form without any purification or solvent exchange, eliminating secondary waste generation and minimizing material loss.
3. *Occupational exposure monitoring.* NCSU EHS performed an air-monitoring assessment for methylene chloride (the representative haloalkane) in the operating laboratory (EB1-1034). The recorded 15-min concentration was < 0.79 ppm, which is well below the EPA short-term exposure limit (16 ppm) and the 8-h TWA (2 ppm). The EHS report confirmed that no additional mitigation was required and recommended continued operation under a fume hood with standard PPE (gloves, lab coat, safety glasses, closed-toe shoes).

Overall, both droplet-scale and continuous-flow experiments minimize reagent consumption and emissions by confining reactions within closed microfluidic channels. This architecture enables precise dosing, complete photon utilization, and efficient halide exchange with negligible waste.

Comment 8: *Carryover controls deserve quantitative evidence. Demonstrating that rinse cycles fully prevent cross-contamination between halide campaigns would strengthen confidence in sequential autonomous operation.*

Response: We appreciate the reviewer's comment. Quantitative evidence confirming the absence of cross-contamination between halide campaigns is already provided in the main text. Specifically, on **page 5, paragraph 2**, the *Results and Discussion* section describes a structured reproducibility study consisting of interleaved baseline and randomized experiments. Following each experiment, the microfluidic platform underwent a standardized rinse cycle using sequential DMF, toluene, and haloalkane flushing to eliminate residual reagents and ensure a contamination-free baseline before subsequent runs. The corresponding **Figure 3C–D** quantitatively presents the reproducibility data, where the coefficients of variation remained below 1.3 % for λ_{em}, 1.8 % for PPLQY, and 1.5 % for FWHM, confirming excellent run-to-run consistency and no evidence of carryover.

Furthermore, the cleaning and rinse protocol is detailed again in the *Methods – FSDL Hardware* section on **page 23, paragraph 2**, where it is stated that each droplet sequence is followed by "*a multistep automated microreactor rinse cycle alternating between DMF, toluene, and DCM to clean the fluidic path*," ensuring full removal of residual materials between PIAER runs. Setup characterization and cleaning validation were performed before initiating all closed-loop campaigns, confirming that every autonomous sequence started from a reproducible and contamination-free baseline.

Comment 9: *Clarify throughput and cost implications by reporting per-day yield, isolated mass, reagent consumption, and energy demand per gram of product.*

Response: We appreciate the reviewer's comment emphasizing the need to clarify throughput and cost implications. In the revised SI document (**Section S16**), we have provided quantitative throughput and reagent balance estimates derived directly from the experimentally validated continuous-flow operating conditions listed in **Tables S1, S11**.

- **Throughput and yield:** At the optimized steady-state residence times (1–4.8 min), the photoreactor processed between 50 mL day^{-1} and 250 mL day^{-1} of NC dispersion depending on the target emission wavelength. Based on the initial $CsPbBr_3$ NC concentration (4.2 µM, measured spectroscopically), and assuming complete conversion to the corresponding mixed-halide composition, the estimated isolated NC yield ranged from 18–25 mg day^{-1} for blue-emitting samples to 28–32 mg day^{-1} for red-emitting samples.
- **Material efficiency and reagent consumption:** For each emission target, we report the total consumption of $CsPbBr_3$ NC feedstock, haloalkane reagent (DCM or 2-iodopropane), and carrier solvents (toluene and DMF) normalized to both throughput and

product mass. For example, the blue-emitting regime (λ_{em} = 480 nm) operated with 0.37 volume fraction DCM at τ = 2 min, corresponding to ~11 µL of haloalkane and 29 µL of total solvent per mg of isolated product.

- **Energy demand:** Electrical energy was determined from the LED current–power calibration (**Figure S3B**)

Under typical 365 nm illumination (0.5–1.4 A drive current), the system consumed 0.9–1.3 Wh mL^{-1} of processed dispersion, corresponding to 30–45 Wh g^{-1} product when normalized by isolated mass, which is significantly lower than conventional batch photoreactors, owing to the short optical pathlength and efficient photon utilization in the microreactor.

These quantitative analyses are now summarized in **Table S11**, covering per-day yield, normalized reagent use, and energy intensity.

It is important to note that scale-up beyond the continuous-flow regime would require post-synthetic purification, solvent recycling, and solid-phase recovery steps to obtain dry NC powders and perform full life-cycle cost accounting. These engineering considerations are beyond the present scope, which focuses on the reaction kinetics, optical optimization, and knowledge-scalability validation of the photoinduced anion-exchange process.

Revised SI, Page S29, highlighted in yellow:

"**S16. Yield and Energy consumption**

Summary of experimentally validated continuous-flow operating conditions corresponding to scaled-up autonomous PIAER campaigns at different emission targets. The table lists the steady-state residence time (τ), daily volumetric throughput, isolated NC yield, haloalkane composition (volumetric fraction), normalized reagent consumption (combined haloalkane + solvent use per milligram of isolated product), and energy demand per gram of NCs. These values were derived from the photon-flux-calibrated microreactor data (**Figure S3B**), and the material balance analysis presented in **Section S7**.

Table S11. *Throughput and Resource Metrics of the Continuous-Flow Photoreactor*

Target λ_{em} (nm)	τ (min)	Throughput (mL day^{-1})	Yield (mg day^{-1})	Haloalkane (vol frac)	Reagent Use (µL mg^{-1} product)	Energy (Wh g^{-1})
405	1.0	50	18–22	DCM (0.41)	40 ± 5	30–38
460	1.5	100	20–24	DCM (0.39)	41 ± 4	32

480	2.0	150	22–25	DCM (0.37)	40 ± 3	35
570	3.5	200	28–30	2-I-Prop (0.45)	44 ± 4	40
600	4.0	220	30–31	2-I-Prop (0.47)	47 ± 3	43
650	4.8	250	31–32	2-I-Prop (0.50)	49 ± 3	45

Revised Manuscript, Page 1, highlighted in yellow:

> "Critically, synthesis protocols discovered at the droplet scale (~10 μL) were directly translated to continuous-flow operation (~50–250 mL·day^{-1}) without reoptimization, maintaining optical performance and establishing knowledge scalability across four orders of magnitude in throughput, with low energy demand."

Revised Manuscript, Page 24, paragraph 1:

> "The developed continuous flow format yields in ~50-250 mL/day of high-performing $CsPbX_3$ NC dispersion with a targeted peak emission wavelength, demonstrating seamless scale-up from single-droplet discovery to continuous manufacturing without altering the fundamental reaction conditions. Quantitative throughput, yield, reagent consumption, and energy metrics derived from these experiments are reported in **Table S11.**"

Comment 10: *A short comparison with other post-synthesis tuning approaches (salt-driven exchange, precursor engineering, thermal diffusion) would help position this work. A table summarizing tunability, linewidth, PLQY retention, scalability, and sustainability could be effective.*

Response: We thank the reviewer for this constructive suggestion. In the revised SI document, we have added a concise comparison summarizing key parameters, including tunability range, emission linewidth, PLQY retention, scalability, and sustainability, across representative post-synthetic bandgap tuning methods, including salt-driven halide exchange, precursor engineering, and thermal diffusion. These conventional approaches typically suffer from diffusion-limited kinetics, broad linewidths, and scalability constraints due to multi-step purification and batch variability. In contrast, our photo-induced anion exchange method offers light-gated control, solvent compatibility, and direct scalability from 10 μL droplets to 250 mL day^{-1} continuous-flow production without reoptimization, establishing a sustainable and programmable route for

precise post-synthesis bandgap tuning of MHP NCs.

Revised SI, Page S30, highlighted in yellow:

"**S17. Post-Synthesis Band-gap Tuning Comparison of MHP NCs.**

Table S12. *Comparison of post-synthetic bandgap-tuning strategies for $CsPbX_3$ nanocrystals*

Approach	Mechanistic Driver	Tunability ($\Delta\lambda_{em}$, nm)	PLQY Retention (%)	FWHM (Linewidth, nm)	Scalability / Throughput	Sustainability / Green-Chemistry Aspect
**Salt-driven halide exchange**[13,14]	Ion diffusion via excess halide salts in polar/nonpolar media	420–640 nm	60–85 % (drops after multiple cycles)	30–45 nm	Batch-limited; poor reproducibility due to mixing heterogeneity	Generates salt-containing effluent; requires purification steps
**Precursor engineering**[15,16]	Controlled halide ratio during nucleation or growth	400–630 nm	80–95 %	25–35 nm	Moderate; dependent on synthesis throughput	Moderate waste; multiple precursor syntheses required
**Thermal diffusion post-treatment**[17]	Thermally driven halide migration	430–600 nm	40–70 %	40–60 nm	Scalable but high energy input; possible lattice degradation	High energy use; thermal decomposition risk
**Photo-induced anion exchange**[1] **(this work)**	Photon-flux-driven halide substitution under flow confinement	405–650 nm (continuous tunability)	90–98 %	20–28 nm	Fully automated; scalable from 10 µL droplets → 250 mL day^{-1} continuous flow	Minimal reagent use; sealed microfluidic system; negligible waste; no purification required

Revised Manuscript, Page 24, Paragraph 1, highlighted in yellow:

"…..Compared to other post-synthetic band-gap-tuning methods, the present photon-driven microfluidic strategy offers broader tunability, higher PLQY retention, and greener continuous-flow scalability (**Table S12**)."

Comment 11: *Ensure the data/code repository is fully usable, with raw spectra, BO logs, and model checkpoints available.*
Response: We thank the reviewer for the comment. The data and code repository have been provided in the original manuscript under the *Data and Code Availability* section.

Comment 12: *Finally, the broader importance of composition tuning for bandgap control and applications should be emphasized. Prior studies have demonstrated how halide mixing can enable color-tunable emission across the visible spectrum. For example, blue–red color-tunable bromide–iodide perovskite nanocrystals synthesized via a saponification technique achieved narrow linewidths and tunable correlated color temperature for white-light-emitting diodes (DOI: 10.1364/JOSAB.36.001616). Similarly, a tetradic phosphor system blending multiple perovskite nanocrystals extended the emission range from violet to infrared, reaching high CRI values for white-light applications (DOI: 10.1039/c9na00125e). Positioning the present light-driven method against these composition-engineering approaches would highlight its novelty and practical advantages.*
Response: We thank the reviewer for this thoughtful suggestion. A clarifying sentence has been added in the *Introduction* to emphasize the broader role of composition-driven bandgap control and to contextualize our light-driven method relative to previous composition-engineering strategies.

Revised Manuscript, Page 2, highlighted in yellow:

> "…composition-tunable bandgap enable efficient charge generation and transport at wavelengths spanning the entire solar spectrum, positioning MHP NCs as promising candidates for low-temperature processed photovoltaics, displays, light-emitting diodes, and photoredox catalysis.[18,19] "

This addition acknowledges prior reports of halide-composition-driven emission control while underscoring the distinguishing features of the present PIAER strategy, its dynamic, reversible tuning, narrow linewidths (20–28 nm), and high PLQY retention (>90 %) within a closed, light-programmable flow system.

Reviewer 2

General comments: *The manuscript discusses a fluidic self-driving laboratory (FSDL) for photo-induced anion exchange reactions (PIAER) in $CsPbX_3$ nanocrystals, integrating Bayesian optimization with microfluidics. As compared to the previous manuscript on similar lines (Adv. Mater. 2025, 37, 2419668) the current study is more focused on mechanistic and kinetic investigation of the PIAER's implementing machine learning (ML)-guided parameter landscape exploration. Most importantly, the authors address the transition of knowledge learnt from microscale to continuous flow operation, it is an interesting aspect.*

Response: We thank the reviewer for their thoughtful summary and positive assessment of our study. The present manuscript indeed builds off of our previous study (*Adv. Mater.* 2025, 37, 2419668) by focusing on the mechanistic and kinetic aspects of the photo-induced anion exchange reaction (PIAER) in $CsPbX_3$ NCs. In this study, we leverage machine learning–guided parameter-space exploration within a fluidic self-driving laboratory (FSDL) to elucidate the interplay between photon flux, halide composition, and NC concentration. Furthermore, we demonstrate direct knowledge transfer from droplet-scale optimization to continuous-flow synthesis without re-optimization, highlighting the FSDL's capability for scalable, data-driven manufacturing of compositionally tuned MHP NCs.

Other comments:

Comment 1: *The manuscript does not introduce new chemistry or methodology, but it does provide a noticeably deeper understanding of the kinetics and mechanisms compared to the group's earlier work.*

Response: We thank the reviewer for the constructive evaluation. We agree that while the present study builds upon our previous study (*Adv. Mater.* 2025, 37, 2419668), its primary contribution lies in advancing the mechanistic and kinetic understanding of photo-induced anion exchange reactions (PIAER) in $CsPbX_3$ NCs. Specifically, this work (i) elucidates the coupled influence of photon flux, NC concentration, and halide precursor fraction on the exchange rate and photoluminescence quantum yield, (ii) integrates Bayesian optimization–guided parameter sensitivity analysis to rank the relative impact of each factor, and (iii) establishes a quantitative kinetic framework validated across both Br→Cl and Br→I systems.

These additions enable a deeper, model-informed interpretation of the PIAER process and demonstrate that the FSDL can serve not only as an optimization tool but also as a platform for unveiling fundamental kinetic mechanisms governing light-driven halide exchange reactions.

Comment 2: *The SHAP analysis is good. They did a detailed analysis of ion exchange, investigating the impact of different input parameters on model prediction and extracting mechanistic insights. It would have been better to have kinetic equations or mechanistic model derived from SHAP insights, instead of qualitative analysis.*

Response: We thank the reviewer for this insightful comment and appreciation of the SHAP-based mechanistic analysis. The goal of this work was to demonstrate how interpretable machine-learning modeling and feature analysis (SHAP) can extract quantitative mechanistic relationships directly from experimental data in an autonomous setting, without imposing predefined kinetic equations that may oversimplify coupled reaction dynamics.

While explicit rate equations were not derived, the SHAP-derived dependencies were quantitatively validated against kinetic fits of the photoinduced anion exchange reactions (**Supplementary Figure S16A**). These analyses reveal two distinct mechanistic regimes: a time-dominated Br→Cl exchange, characteristic of diffusion-limited lattice substitution, and a photon-flux-dominated Br→I exchange, reflecting photo-activated halide incorporation through radical intermediates. The SHAP trends reproduce the experimentally observed rate behaviors ($|k|$ *vs*. flux; $|k|$ vs. time) and agree with prior mechanistic reports on halide migration energetics in $CsPbX_3$ NCs. [5,20]

We view this approach as complementary to kinetic modeling, where SHAP analysis captures nonlinear, multi-parameter coupling that is often inaccessible to traditional fitting. Future work will integrate these data-driven dependencies into physics-informed surrogate kinetic models, enabling automated mechanism discovery within an SDL framework.

Revised SI, Page S27, highlighted in yellow:

"**S14. Kinetics and control experiment**

To quantify the light-driven kinetics of the photo-induced anion exchange reactions, intrinsic rate constants ($|k|$) were extracted from the UV-on segments of the spectral trajectories and correlated with the actinometry-derived photon flux. The resulting linear dependence (**Figure S16A**) confirms that both Br→Cl and Br→I exchanges proceed under photon-limited conditions. The steeper slope for Br→Cl indicates faster intrinsic kinetics and a lower activation barrier relative to Br→I substitution.[2] Control experiments performed under identical flow and chemical conditions but without UV illumination (**Figure S16B–C**) show no measurable shift in emission or absorbance over the entire residence time, verifying that the exchange is inactive in the dark and proceeds exclusively under irradiation.

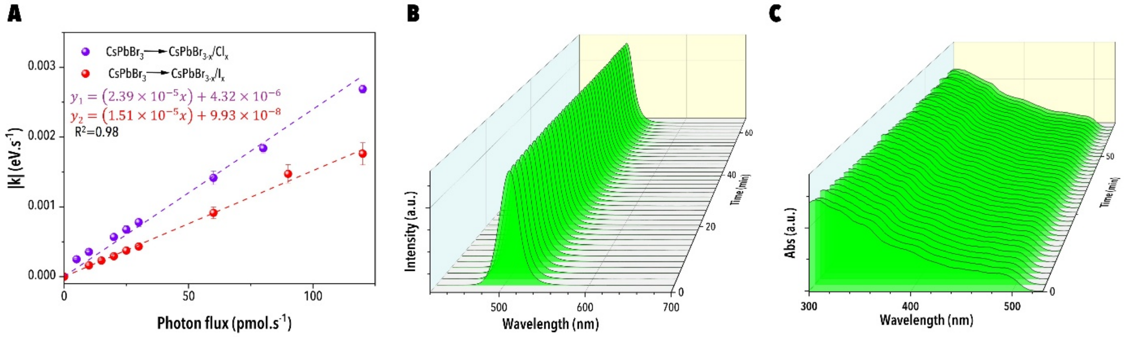

Figure S16. *Intrinsic kinetics and dark-control validation for photo-induced anion exchange reactions. (A) Dependence of the intrinsic kinetic rate constant (|k|, eV·s^{-1}) on the applied photon flux (pmol·s^{-1}) for the Br→Cl (purple) and Br→I (red) exchanges in $CsPbBr_3$ NCs. Both reactions display a linear flux dependence, confirming a photon-limited regime, with the Br→Cl exchange proceeding faster due to its lower activation barrier and enhanced halide mobility. (B, C) Time-resolved photoluminescence (PL) and absorption (Abs) spectra for a dark-control experiment ([NC] = 4.2 µM, [1-iodopropane] = 0.13 M, UV LED off, residence time = 65 min, T = 23 °C). No measurable spectral evolution is observed over time, confirming that halide exchange is inactive in the absence of illumination. Together, these data demonstrate that the reaction is strictly light-activated, thermally stable, and governed by photon-flux-dependent intrinsic kinetics.*"

Comment 3: *Their reactions run for hours, with the UV light on when the droplet is oscillating. With such a duration, heat generated can impact the reaction within droplets (they do mention photodegradation at high fluxes, which can be both caused by overexposure or heat). It is mentioned that temperature stays constant at 23C, but no mention of any thermal control system. Perhaps they can mention that the aluminium reactor act as a heat sink and temperature is measured to be constant.*

Response: We thank the reviewer for this valuable observation. The microfluidic photoreactor body is CNC-machined from aluminum, which acts as an efficient passive heat sink, dissipating any thermal load from the UV source during extended operation. Reactor temperature was continuously monitored using a Traceable® thermocouple thermometer positioned adjacent to the flow cell throughout prolonged PIAER experiments (3 h) conducted at a photon flux of 120 pmol s^{-1} (25 mA LED input). The temperature remained constant at 23 ± 0.02 °C, confirming negligible photothermal heating. This clarification has been added to the revised SI document, *Section S1 (Microfluidic Photoreactor)* and referenced in the main text to explicitly note the reactor's passive thermal regulation and continuous temperature monitoring.

Revised SI, Page S31, highlighted in yellow:

"**S18. Thermal Stability of the Microfluidic Photoreactor**

The aluminum reactor body served as a passive heat sink, maintaining the reactor temperature constant at 23 ± 0.02 °C throughout prolonged (3 h) UV irradiation at a photon flux of 120 pmol s^{-1}, as verified by *in situ* temperature monitoring.

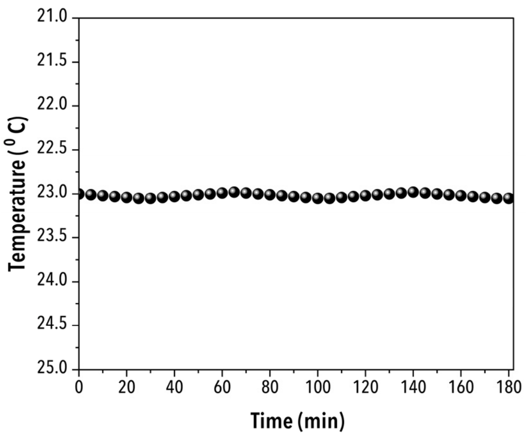

Figure S18. *Thermal stability of the microfluidic photoreactor under prolonged UV irradiation. Microfluidic reactor temperature as a function of time during a 3 h PIAER experiment conducted at a photon flux of 120 pmol s^{-1} (25 mA LED input). The temperature remained stable at 23 ± 0.02 °C, confirming negligible photothermal heating. The aluminum reactor body acts as a passive heat sink, dissipating the UV-induced thermal load during continuous operation*."

Revised Manuscript, Page 6, highlighted in yellow:

"Continuous UV irradiation tests further confirmed negligible photothermal heating of the microfluidic reactor, with temperature maintained at 23 ± 0.02 °C during 3 h of operation at the highest photon flux (120 pmol s^{-1}), indicating that PIAER is governed purely by photochemical processes rather than thermal effects (**Figure S18**)."

Comment 4: *The scalability claim is also crucial. Comparing microfluidics vs scalable flow should be done at equivalent photon flux per unit area/volume, have they ensured it?*

Response: We thank the reviewer for this important comment. Both the single-droplet and continuous-flow photoreactors were operated under photon-flux-normalized conditions, ensuring equivalent optical energy input per unit illuminated volume. The photon flux in each reactor geometry was quantified by 4,4′-dimethylazobenzene actinometry (**Figure S3**), which provided a calibration between LED current and photon flux (pmol s^{-1}). During scale-up, the LED power density and illuminated reactor cross-section were adjusted such that the photon flux

per unit illuminated volume (Φ/V) matched that of the single-droplet discovery mode within ±5%.

We have clarified this in the revised *Knowledge Scalability* section and included supporting quantitative evidence in **Tables S9–S11**, which summarize (i) cross-scale optical reproducibility (< 5% deviation in λ_{em}, FWHM, and PLQY), (ii) steady-state reproducibility (CV < 6%), and (iii) equivalent photon-normalized energy demand (30–45 Wh g^{-1} product). These results confirm that the observed "knowledge scalability" arises from intrinsic process reproducibility rather than differences in illumination or photon transport.

Comment 5: *Figure 5 has an issue with the numbering of the sub figures. Not consistent (last two rows).*

Response: We thank the reviewer for catching this oversight. The subfigure lettering in the revised Figure 5 and its caption has been corrected to ensure complete and sequential alphabetical ordering.

Comment 6: *Overall, it is a comprehensive study. Compared with the group's previous publication, this study introduces two notable advances: the use of Bayesian optimization for parameter ranking and the scale-up of synthesis via continuous-flow processing. However, while scalability and process optimization are well addressed, in my opinion the manuscript omits a mathematical understanding of reaction kinetics which will be very useful to include.*

Response: We thank the reviewer for this constructive suggestion and for recognizing the advances introduced in this work. The present study was designed to establish a data-driven, experimentally validated framework capable of extracting kinetic insight directly from autonomous experimentation, rather than prescribing rate equations a priori.

Within this framework, Bayesian optimization identifies the most influential experimental variables, while SHAP-based interpretability quantifies their mechanistic contributions. Together, these analyses revealed two distinct regimes: a time-dominated Br→Cl exchange characteristic of diffusion-limited lattice substitution, and a photon-flux-dominated Br→I exchange arising from photo-activated halide incorporation. These trends are quantitatively consistent with the kinetic fits presented in the revised SI document ($|k|$ *vs*. flux and $|k|$ *vs*. time) and align with established halide-migration energetics in $CsPbX_3$ NCs.

Although a complete analytical rate model was beyond the scope of the current work, the SHAP-derived relationships coupled with experimental rate constants together form a semi-quantitative kinetic description of the process. Building upon this foundation, our ongoing efforts focus on integrating these data-driven dependencies into physics-informed surrogate models, enabling automated, quantitative mechanism discovery within a self-driving laboratory framework.

Revised SI, Page S27, highlighted in yellow:

"**S14. Kinetics and control experiment**

To quantify the light-driven kinetics of the photo-induced anion exchange reactions, intrinsic rate constants ($|k|$) were extracted from the UV-on segments of the spectral trajectories and correlated with the actinometry-derived photon flux. The resulting linear dependence (**Figure S16A**) confirms that both Br→Cl and Br→I exchanges proceed under photon-limited conditions. The steeper slope for Br→Cl indicates faster intrinsic kinetics and a lower activation barrier relative to Br→I substitution.[2] Control experiments performed under identical flow and chemical conditions but without UV illumination (**Figure S16B–C**) show no measurable shift in emission or absorbance over the entire residence time, verifying that the exchange is inactive in the dark and proceeds exclusively under irradiation.

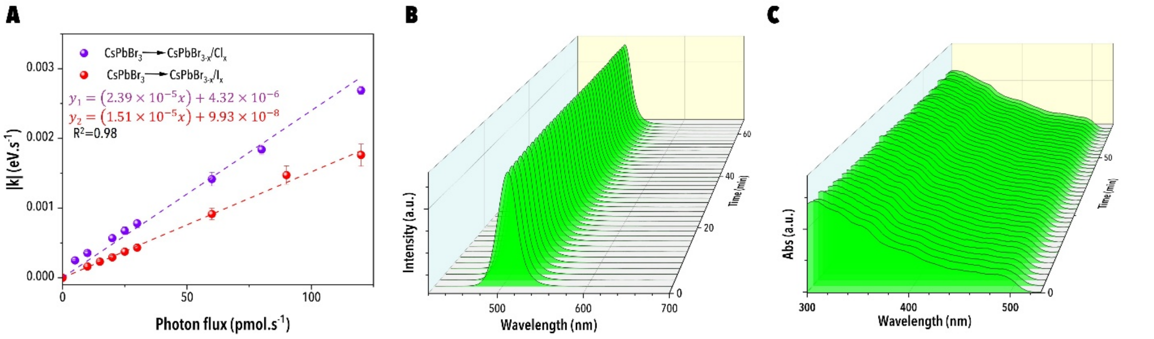

Figure S16. *Intrinsic kinetics and dark-control validation for photo-induced anion exchange reactions. (A) Dependence of the intrinsic kinetic rate constant ($|k|$, $eV{\cdot}s^{-1}$) on the applied photon flux ($pmol{\cdot}s^{-1}$) for the Br→Cl (purple) and Br→I (red) exchanges in $CsPbBr_3$ NCs. Both reactions display a linear flux dependence, confirming a photon-limited regime, with the Br→Cl exchange proceeding faster due to its lower activation barrier and enhanced halide mobility. (B, C) Time-resolved photoluminescence (PL) and absorption (Abs) spectra for a dark-control experiment ([NC] = 4.2 μM, [1-iodopropane] = 0.13 M,* ***,*** *UV LED off, residence time = 65 min, T = 23 °C). No measurable spectral evolution is observed over time, confirming that halide exchange is inactive in the absence of illumination. Together, these data demonstrate that the reaction is strictly light-activated, thermally stable, and governed by photon-flux-dependent intrinsic kinetics.*"

Revised Manuscript, Page 6, Paragraph 1, highlighted in yellow:

> "…. These results confirm that halide exchange is strictly light-activated and can be precisely modulated by adjusting the duration and intensity of the photoexcitation. Quantitative kinetic analysis of the UV-on segments confirmed photon-flux-dependent reaction rates, establishing a linear correlation between the intrinsic rate constant and the actinometry-calibrated photon flux (**Figure S16**). Control experiments performed under identical conditions but without UV illumination showed no measurable spectral change, verifying that the halide exchange is inactive in the dark."

References:

(1) Jha, P.; Mukhin, N.; Ghorai, A.; Morshedian, H.; Canty, R. B.; Delgado-Licona, F.; Brown, E. E.; Pyrch, A. J.; Castellano, F. N.; Abolhasani, M. Photo-Induced Bandgap Engineering of Metal Halide Perovskite Quantum Dots In Flow. *Advanced Materials* **2025**, *37* (16), 2419668. https://doi.org/10.1002/adma.202419668.

(2) Jha, P.; Mukhin, N.; Ghorai, A.; Morshedian, H.; Canty, R. B.; Delgado-Licona, F.; Brown, E. E.; Pyrch, A. J.; Castellano, F. N.; Abolhasani, M. Photo-Induced Bandgap Engineering of Metal Halide Perovskite Quantum Dots In Flow. *Advanced Materials n/a* (n/a), 2419668. https://doi.org/10.1002/adma.202419668.

(3) Livakas, N.; Toso, S.; Ivanov, Y. P.; Das, T.; Chakraborty, S.; Divitini, G.; Manna, L. CsPbCl3 → CsPbI3 Exchange in Perovskite Nanocrystals Proceeds through a Jump-the-Gap Reaction Mechanism. *J. Am. Chem. Soc.* **2023**, *145* (37), 20442–20450. https://doi.org/10.1021/jacs.3c06214.

(4) Lai, M.; Obliger, A.; Lu, D.; Kley, C. S.; Bischak, C. G.; Kong, Q.; Lei, T.; Dou, L.; Ginsberg, N. S.; Limmer, D. T.; Yang, P. Intrinsic Anion Diffusivity in Lead Halide Perovskites Is Facilitated by a Soft Lattice. *Proceedings of the National Academy of Sciences* **2018**, *115* (47), 11929–11934. https://doi.org/10.1073/pnas.1812718115.

(5) Parobek, D.; Dong, Y.; Qiao, T.; Rossi, D.; Son, D. H. Photoinduced Anion Exchange in Cesium Lead Halide Perovskite Nanocrystals. *J. Am. Chem. Soc.* **2017**, *139* (12), 4358–4361. https://doi.org/10.1021/jacs.7b01480.

(6) Wong, Y.-C.; Wu, W.-B.; Wang, T.; Ng, J. D. A.; Khoo, K. H.; Wu, J.; Tan, Z.-K. Color Patterning of Luminescent Perovskites via Light-Mediated Halide Exchange with Haloalkanes. *Advanced Materials* **2019**, *31* (24), 1901247. https://doi.org/10.1002/adma.201901247.

(7) Hu, L.; Guan, X.; Huang, H.; Ye, T.; Ding, J.; Aarti, A.; Venkatesan, K.; Wang, W.; Chen, F.; Lin, C.-H.; Wan, T.; Li, M.; Yi, J.; Zheng, R.; Chu, D.; Cai, S.; Chen, J.; Cazorla, C.; Yuan, J.; Bai, Y.; Wu, T.; Huang, S. Assessing the Optoelectronic Performance of Halide Perovskite Quantum Dots

with Identical Bandgaps: Composition Tuning Versus Quantum Confinement. *ACS Energy Lett.* **2024**, *9* (8), 3970–3981. https://doi.org/10.1021/acsenergylett.4c01180.

(8) Wang, S.; Wang, Y.; Zhang, Y.; Zhang, X.; Shen, X.; Zhuang, X.; Lu, P.; Yu, W. W.; Kershaw, S. V.; Rogach, A. L. Cesium Lead Chloride/Bromide Perovskite Quantum Dots with Strong Blue Emission Realized via a Nitrate-Induced Selective Surface Defect Elimination Process. *J. Phys. Chem. Lett.* **2019**, *10* (1), 90–96. https://doi.org/10.1021/acs.jpclett.8b03750.

(9) Ghorai, A.; Mahato, S.; Srivastava, S. K.; Ray, S. K. Atomic Insights of Stable, Monodispersed CsPbI3−xBrx (x = 0, 1, 2, 3) Nanocrystals Synthesized by Modified Ligand Cell. *Advanced Functional Materials* **2022**, *32* (32), 2202087. https://doi.org/10.1002/adfm.202202087.

(10) López, C. A.; Abia, C.; Alvarez-Galván, M. C.; Hong, B.-K.; Martínez-Huerta, M. V.; Serrano-Sánchez, F.; Carrascoso, F.; Castellanos-Gómez, A.; Fernández-Díaz, M. T.; Alonso, J. A. Crystal Structure Features of CsPbBr3 Perovskite Prepared by Mechanochemical Synthesis. *ACS Omega* **2020**, *5* (11), 5931–5938. https://doi.org/10.1021/acsomega.9b04248.

(11) Berezovska, Y.; Sabisch, S.; Bernasconi, C.; Sahin, Y.; Bertolotti, F.; Guagliardi, A.; Bodnarchuk, M. I.; Dirin, D. N.; Kovalenko, M. V. Tightly yet Dynamically Bound Aliphatic Guanidinium Ligands for Lead Halide Perovskite Nanocrystals. *J. Am. Chem. Soc.* **2025**, *147* (39), 35446–35455. https://doi.org/10.1021/jacs.5c09354.

(12) He, Y.; Zhang, L.; Chen, G.; Liu, Y.; Shi, S.; Jiang, P.; Ding, J.; Xu, S.; Geng, C. ZnO/SiO2 Encapsulation of Perovskite Nanocrystals for Efficient and Stable Light-Emitting Diodes. *Applied Surface Science* **2023**, *611*, 155724. https://doi.org/10.1016/j.apsusc.2022.155724.

(13) Akkerman, Q. A.; D'Innocenzo, V.; Accornero, S.; Scarpellini, A.; Petrozza, A.; Prato, M.; Manna, L. Tuning the Optical Properties of Cesium Lead Halide Perovskite Nanocrystals by Anion Exchange Reactions. *J. Am. Chem. Soc.* **2015**, *137* (32), 10276–10281. https://doi.org/10.1021/jacs.5b05602.

(14) Abdel-Latif, K.; Epps, R. W.; Kerr, C. B.; Papa, C. M.; Castellano, F. N.; Abolhasani, M. Facile Room-Temperature Anion Exchange Reactions of Inorganic Perovskite Quantum Dots Enabled by a Modular Microfluidic Platform. *Advanced Functional Materials* **2019**, *29* (23), 1900712. https://doi.org/10.1002/adfm.201900712.

(15) Prasanna, R.; Gold-Parker, A.; Leijtens, T.; Conings, B.; Babayigit, A.; Boyen, H.-G.; Toney, M. F.; McGehee, M. D. Band Gap Tuning via Lattice Contraction and Octahedral Tilting in Perovskite Materials for Photovoltaics. *J. Am. Chem. Soc.* **2017**, *139* (32), 11117–11124. https://doi.org/10.1021/jacs.7b04981.

(16) Ramos-Terrón, S.; Martín, C.; Miguel, G. de; Solano, E.; Hermida-Merino, D.; Vondel, J. V. de; Hofkens, J.; Keshavarz, M. A-Site Cation Engineering and Halide Tuning via Precursor Engineering to Tune the Optical Properties of 2D Perovskites. *RSC Adv.* **2025**, *15* (34), 28181–28190. https://doi.org/10.1039/D5RA03422A.

(17) Yamauchi, M.; Fujiwara, Y.; Masuo, S. Slow Anion-Exchange Reaction of Cesium Lead Halide Perovskite Nanocrystals in Supramolecular Gel Networks. *ACS Omega* **2020**, *5* (24), 14370–14375. https://doi.org/10.1021/acsomega.0c00880.

(18) Thapa, S.; Adhikari, G. C.; Zhu, H.; Zhu, P. Blue-Red Color-Tunable All-Inorganic Bromide–Iodide Mixed-Halide Perovskite Nanocrystals Using the Saponification Technique for White-Light-Emitting Diodes. *J. Opt. Soc. Am. B, JOSAB* **2019**, *36* (6), 1616–1622. https://doi.org/10.1364/JOSAB.36.001616.

(19) Adhikari, G. C.; Vargas, P. A.; Zhu, H.; Grigoriev, A.; Zhu, P. Tetradic Phosphor White Light with Variable CCT and Superlative CRI through Organolead Halide Perovskite Nanocrystals. *Nanoscale Adv.* **2019**, *1* (5), 1791–1798. https://doi.org/10.1039/C9NA00125E.

(20) Gopika, K. Y.; Aparna, S. K.; Akshaya, K.; Sandeep, K. Unraveling the Mechanism of Light-Induced Solvent-Mediated Anion Exchange Reactions in CsPbBr3 Perovskite Nanocrystals. *J. Phys. Chem. C* **2025**, *129* (28), 13123–13130. https://doi.org/10.1021/acs.jpcc.5c03580.
